# Supplementary material for: Multimodal surveillance of SARS-CoV-2 at a university enables development of a robust outbreak response framework
Source: Med. 2022 Dec 9;3(12):883–900.e13. doi: 10.1016/j.medj.2022.09.003 (PMC9482833; doi:10.1016/j.medj.2022.09.003)
Supplement: Document S1. Figures S1–S12 [file mmc1.pdf]

Med, Volume *3*

Supplemental information

Multimodal surveillance of SARS-CoV-2 at a university enables development of a robust outbreak response framework

Brittany A. Petros, Jillian S. Paull, Christopher H. Tomkins-Tinch, Bryn C. Loftness, Katherine C. DeRuff, Parvathy Nair, Gabrielle L. Gionet, Aaron Benz, Taylor Brock-Fisher, Michael Hughes, Leonid Yurkovetskiy, Shandukani Mulaudzi, Emma Leenerman, Thomas Nyalile, Gage K. Moreno, Ivan Specht, Kian Sani, Gordon Adams, Simone V. Babet, Emily Baron, Jesse T. Blank, Chloe Boehm, Yolanda Botti-Lodovico, Jeremy Brown, Adam R. Buisker, Timothy Burcham, Lily Chylek, Paul Cronan, Ann Dauphin, Valentine Desreumaux, Megan Doss, Belinda Flynn, Adrianne Gladden-Young, Olivia Glennon, Hunter D. Harmon, Thomas V. Hook, Anton Kary, Clay King, Christine Loreth, Libby Marrs, Kyle J. McQuade, Thorsen T. Milton, Jada M. Mulford, Kyle Oba, Leah Pearlman, Mark Schifferli, Madelyn J. Schmidt, Grace M. Tandus, Andy Tyler, Megan E. Vodzak, Kelly Krohn Bevill, Andres Colubri, Bronwyn L. MacInnis, A. Zeynep Ozsoy, Eric Parrie, Kari Sholtes, Katherine J. Siddle, Ben Fry, Jeremy Luban, Daniel J. Park, John Marshall, Amy Bronson, Stephen F. Schaffner, and Pardis C. Sabeti

Supplemental Items

<u>Figure S1.</u> Incidence rates at Colorado Mesa University (CMU), with surrounding areas for context, and epidemiological risk factors for SARS-CoV-2 positivity at CMU. Related to Figure 2.

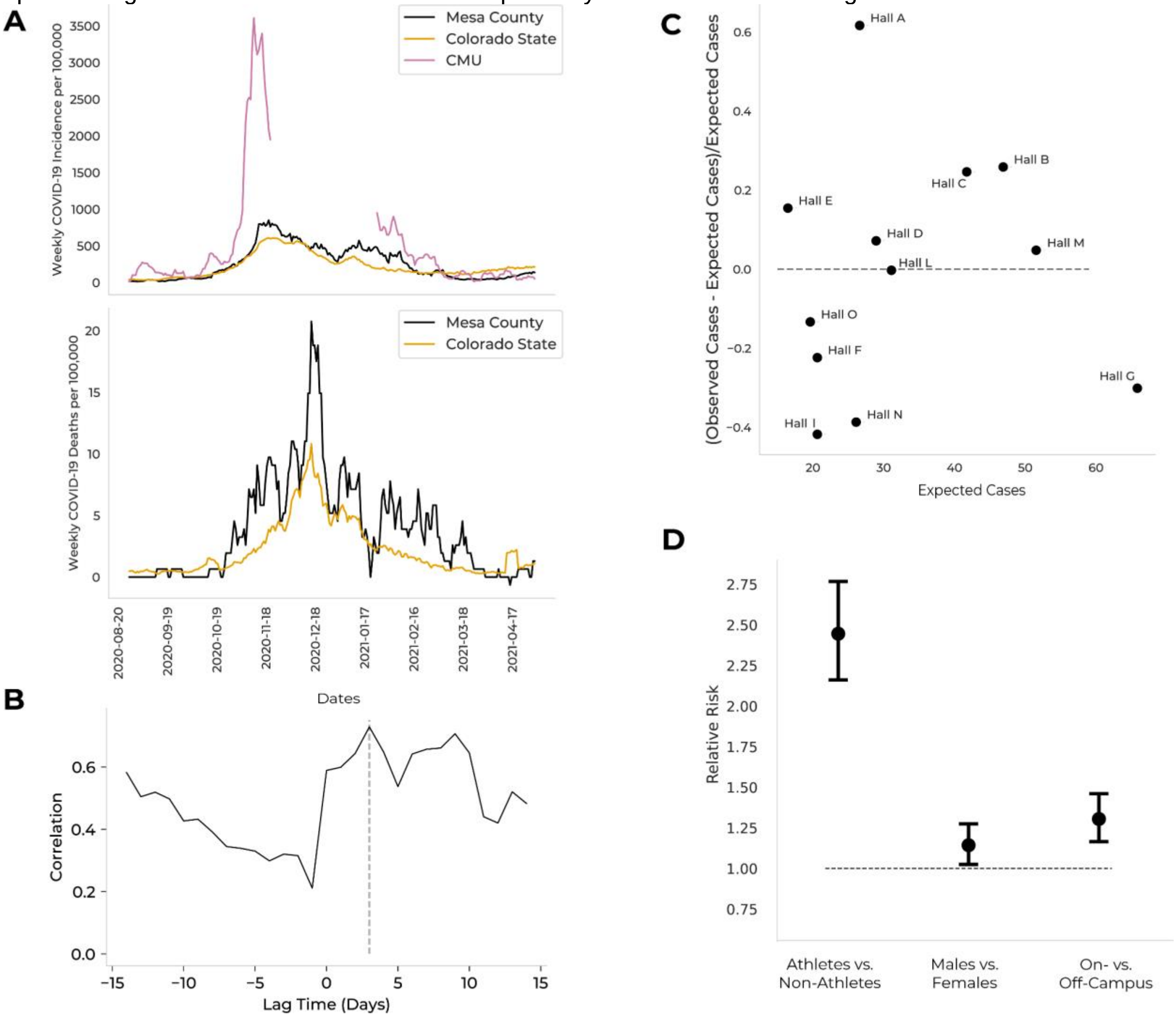

A. Weekly COVID-19 incidence at Colorado Mesa University (CMU; pink), in Mesa County (black), and in Colorado (orange) over the 2020–2021 academic year (upper). Weekly COVID-19 death rate in Mesa County (black) and in Colorado (orange) over the 2020–2021 academic year (lower). Data from CMU were not analyzed from November 21–January 18 due to winter recess. The correlations between CMU incidence rates and Mesa County incidence rates (correlation = 0.73, lag = 3 days) and Mesa County death rates (correlation = 0.48, lag = 8 days) were tested with lag times between -14 days (0 days for deaths) and 14 days, and the maximum correlations and corresponding lag times are reported. **B.** The correlation between CMU incidence rates and Mesa County incidence rates *vs.* the lag time. The dashed line at $y = 3$ indicates the lag time that maximizes the cross-correlation. **C.** The difference between the number of cases observed and the number of cases expected (given residence hall population size and scaled by number of cases expected) *vs.* the number of cases expected, per residence hall. The dashed line at $y = 0$ separates halls with more (above) or fewer (below) cases observed than expected. **D.** Relative risk (RR) of testing positive for COVID-19 given sports team membership (athletes *vs.* non-athletes), sex (males *vs.* females), or residential status (on- *vs.* off-campus). Circles represent the relative risk, with whiskers extending to the upper and lower bounds of the 95% confidence interval. The dashed line at RR = 1 represents the null

hypothesis (*i.e.*, that there is no association between the risk factor and test positivity). An RR greater than 1 implies that the first group listed (*i.e.*, athletes, males, or on-campus students) had a greater risk of testing positive than the second group listed (*i.e.*, non-athletes, females, or off-campus students). Athletes were tested 1.55 times as often as non-athletes and on-campus students were tested 1.80 times as often as off-campus students; thus, sports participation was associated with increased risk of SARS-CoV-2 positivity, while residential living was not.

<u>Figure S2.</u> Detection of outbreak groups using viral genomic sequencing of cases. Related to Figure 4.

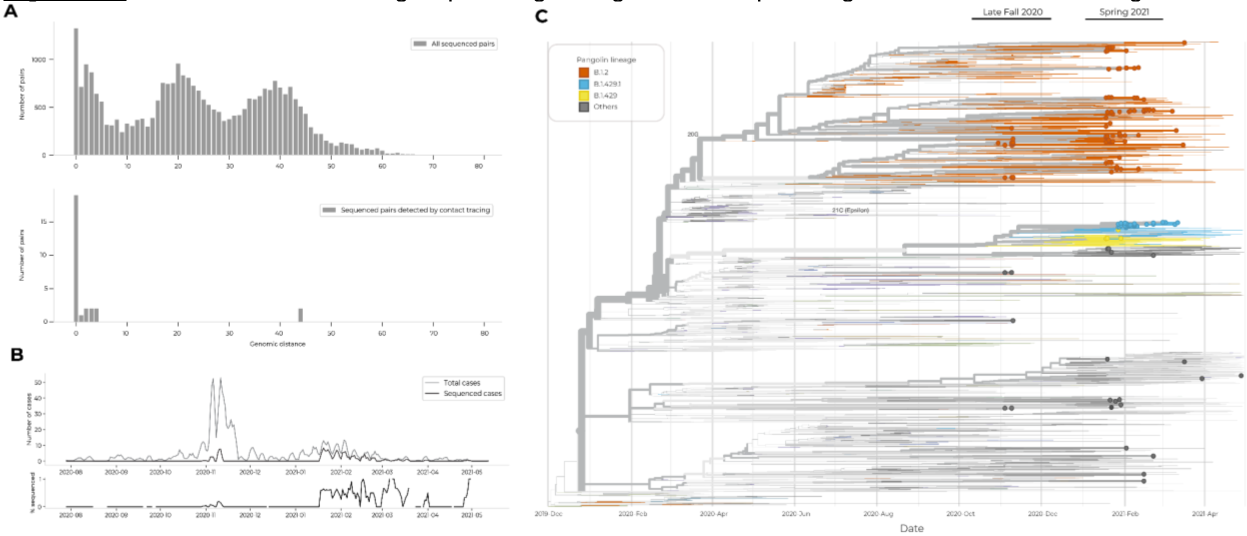

A. Distributions of genetic distance for all pairs of viral genomes (upper) and for pairs of viral genomes from positive individuals sharing interactions reported during contact tracing (lower). Two outlier pairs (lower) had a genetic distance of 44 SNVs. In each of these pairs, an individual testing positive at the end of January 2021 had virus of the B.1.2 lineage and indicated close contact with an individual who tested positive in early February (5-6 days later) with virus of the B.1.429.1 lineage. **B.** Sequencing of positive cases via genomic surveillance across both semesters. Sequencing began October 2020, capturing cases associated with a Halloween-related outbreak. Shown are the total number of cases (top, thin line), total number of sequenced cases (top, bolded line), and percent of positive cases that were sequenced (bottom). **C.** Phylogenetic placement and temporal sampling of viral genomes sequenced from CMU clinical diagnostic tests. CMU nodes are depicted as dots among a global tree of contextual sequences, weighted toward sequences collected from Colorado and surrounding states and genomes close in genetic distance to CMU sequences. Interactive version available at https://auspice.broadinstitute.org.

Figure S3. Summary of wastewater surveillance implemented at Colorado Mesa University. Related to Figure 5.

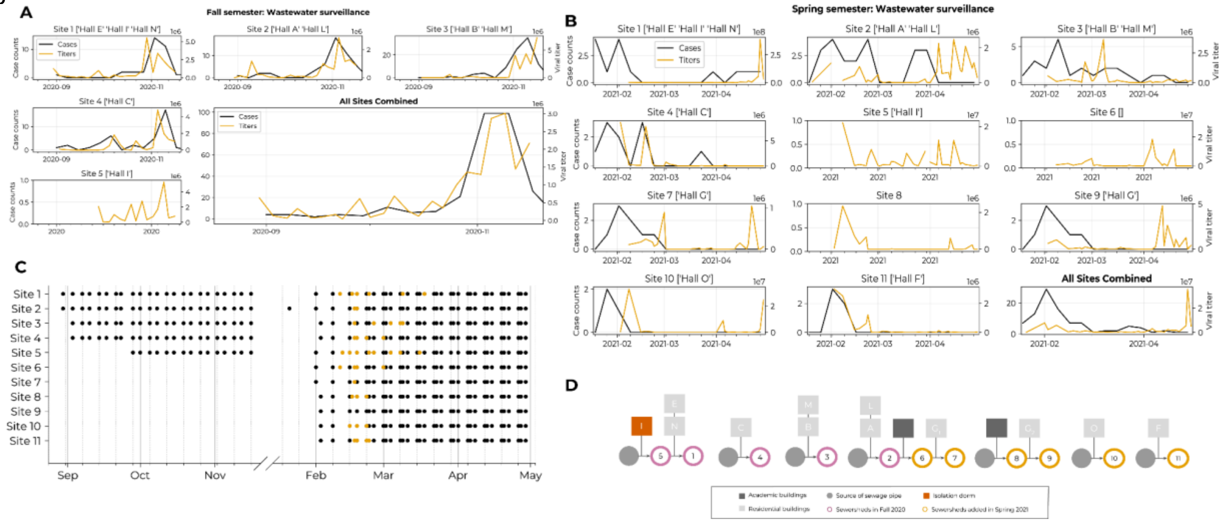

A-B. Comparison of wastewater viral titer (orange) and weekly case count (black) for each wastewater site in the Fall semester (**A**) and Spring semester (**B**). **C.** Sample collection frequency for wastewater surveillance in the 2020–2021 academic year. Wastewater samples were collected twice weekly in the Fall across 5 sites, and three times weekly in the Spring across 11 sites. Each dot represents an independent collection event, with dots shown in orange representing sequenced wastewater samples. **D.** Schematic showing the residential or academic buildings (squares) that contributed effluent to each monitored wastewater site (circles). Residence hall I (red square) housed a small number of students who had tested positive and were in isolation. Wastewater sites are represented by circles, with filled-in gray circles representing sites used to collect baseline measurements (*i.e.*, with no upstream contributors to the particular sewage system). Sites shown in pink were added in Fall 2020 and remained in use into Spring 2021, while sites shown in orange were added in Spring 2021.

Figure S4. Predictors of COVID-19 incidence rates in Colorado Mesa University residence halls *via* a linear regression model. Related to Figure 2.

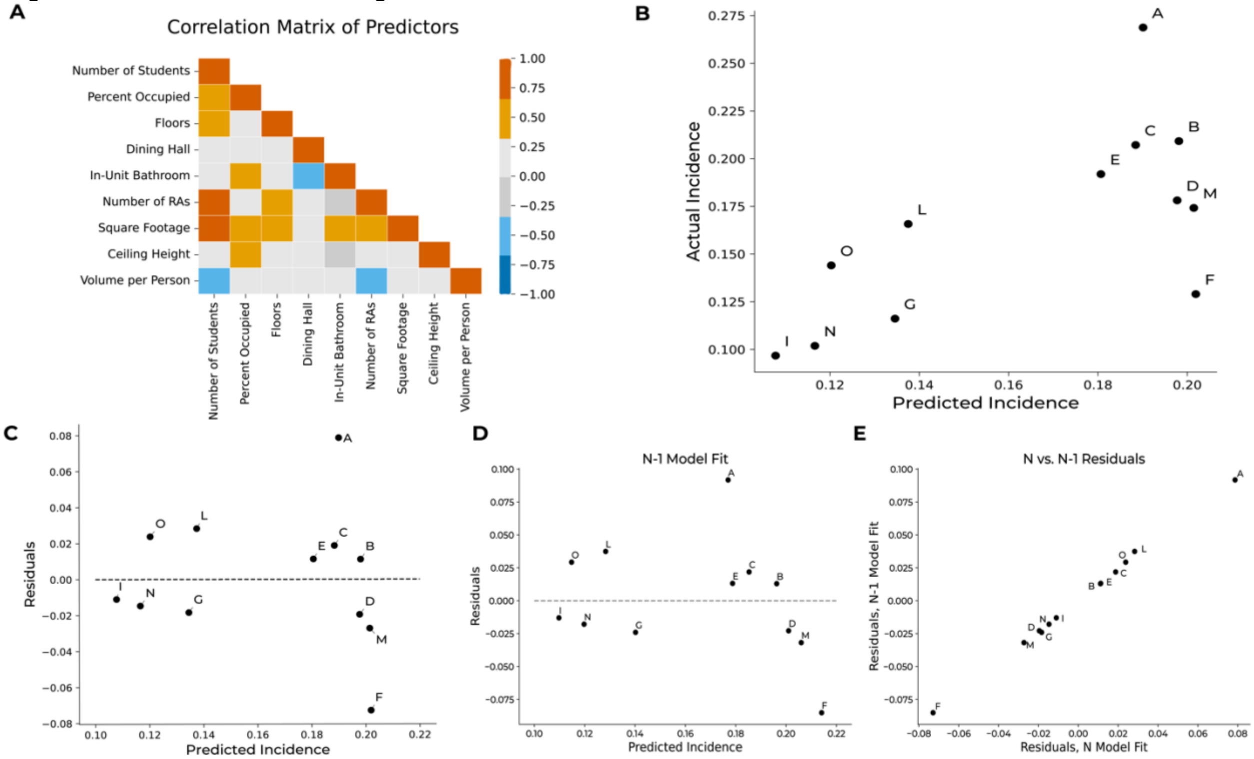

A. Descriptive attributes of residence halls, including number of students, percent occupied (number of available beds / number of students), number of floors, the presence of a meal plan requirement (“dining hall”), the presence of an in-unit bathroom (“private bath”), the number of resident advisors (RAs), square footage, ceiling height, and volume per person, were used as predictors in the regression model. Non-zero correlation coefficients between pairs of predictive variables indicate multicollinearity. **B.** Observed *vs.* predicted incidence rates, by residence hall. Predictions come from the model that minimized the Akaike and Bayesian Information Criteria. **C.** Residuals *vs.* predicted incidence rates, by residence hall. Root-mean-square-error = 3.6%. Halls A and F contribute to heteroscedasticity. **D.** Residuals *vs.* predicted incidence rates, from leave-one-out cross-validation, by residence hall. Leave-one-out cross-validation (*i.e.*, *N*-1 model fit, where *N* = number of residence halls) was conducted such that each hall’s incidence rate was predicted *via* a linear model whose coefficients were determined with training data from all other halls. Root-mean-square-error = 4.2%. **E.** Residuals from the leave-one-out cross-validation (*N*-1 model fit) *vs.* residuals from the full model (*N* model fit).

Figure S5. On-campus presence, as inferred by WiFi proximity data, by semester and by user category. Related to Figure 3.

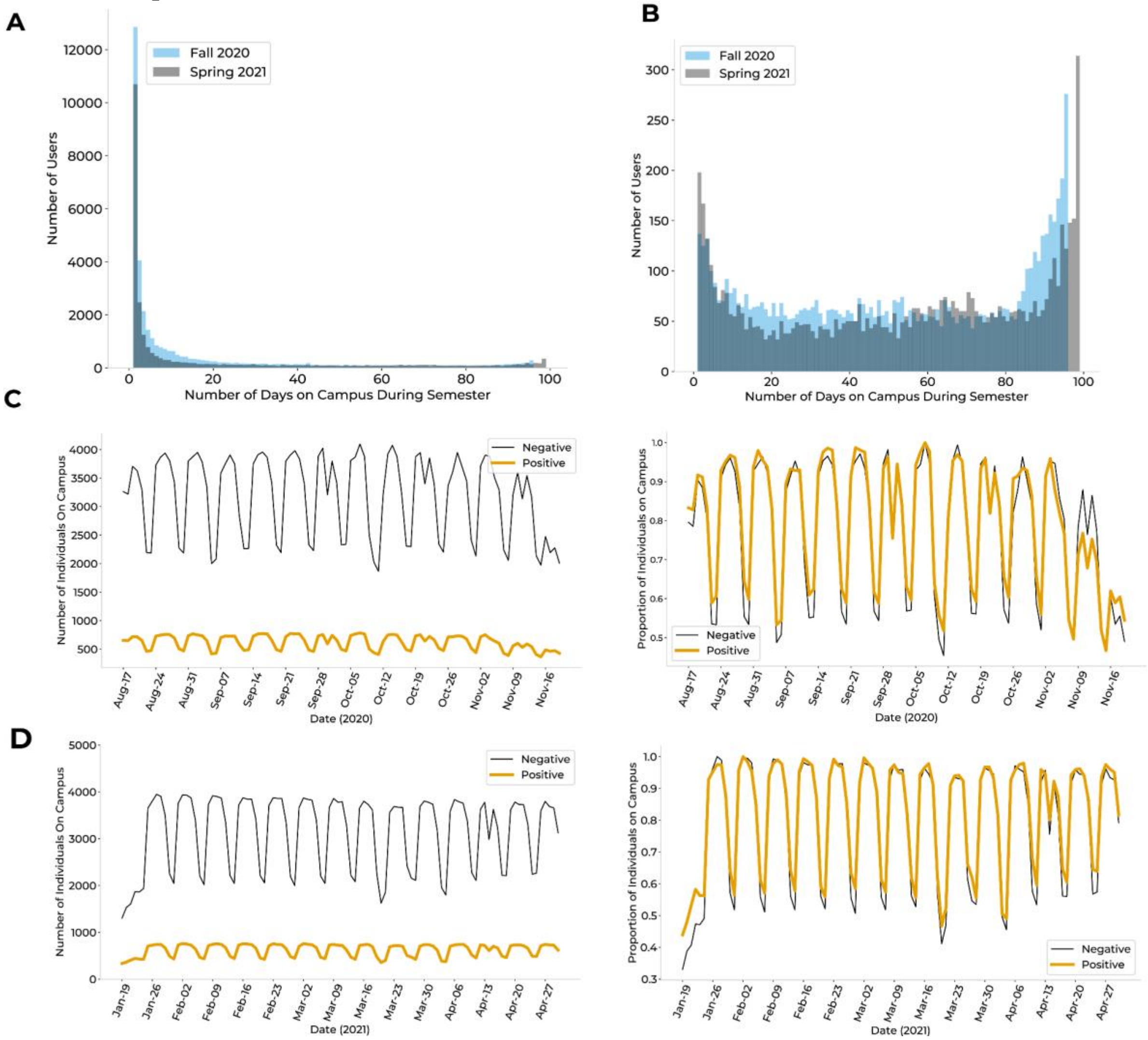

A. The number of days present on campus for all users found in the WiFi network, colored by semester. **B.** The number of days present on campus for all users found in the WiFi network after removal of unauthenticated users, non-students, and infrequent users, colored by semester. This cleaned WiFi proximity network was used for all future analyses. **C.** Number (left) and proportion (right) of positive students (orange) and negative students (black) on campus by day for Fall 2020. There was no significant difference in the distributions ($p = 0.282$), which correlated with one another (Pearson correlation: 0.976, $p < 0.001$). **D.** Number (left) and proportion (right) of positive students (orange) and negative students (black) on campus by day for Spring. There was no significant difference in the distributions ($p = 0.248$), which correlated with one another (Pearson correlation: 0.99, $p < 0.001$).

Figure S6. WiFi network access patterns by building and by semester. Related to Figure 3.

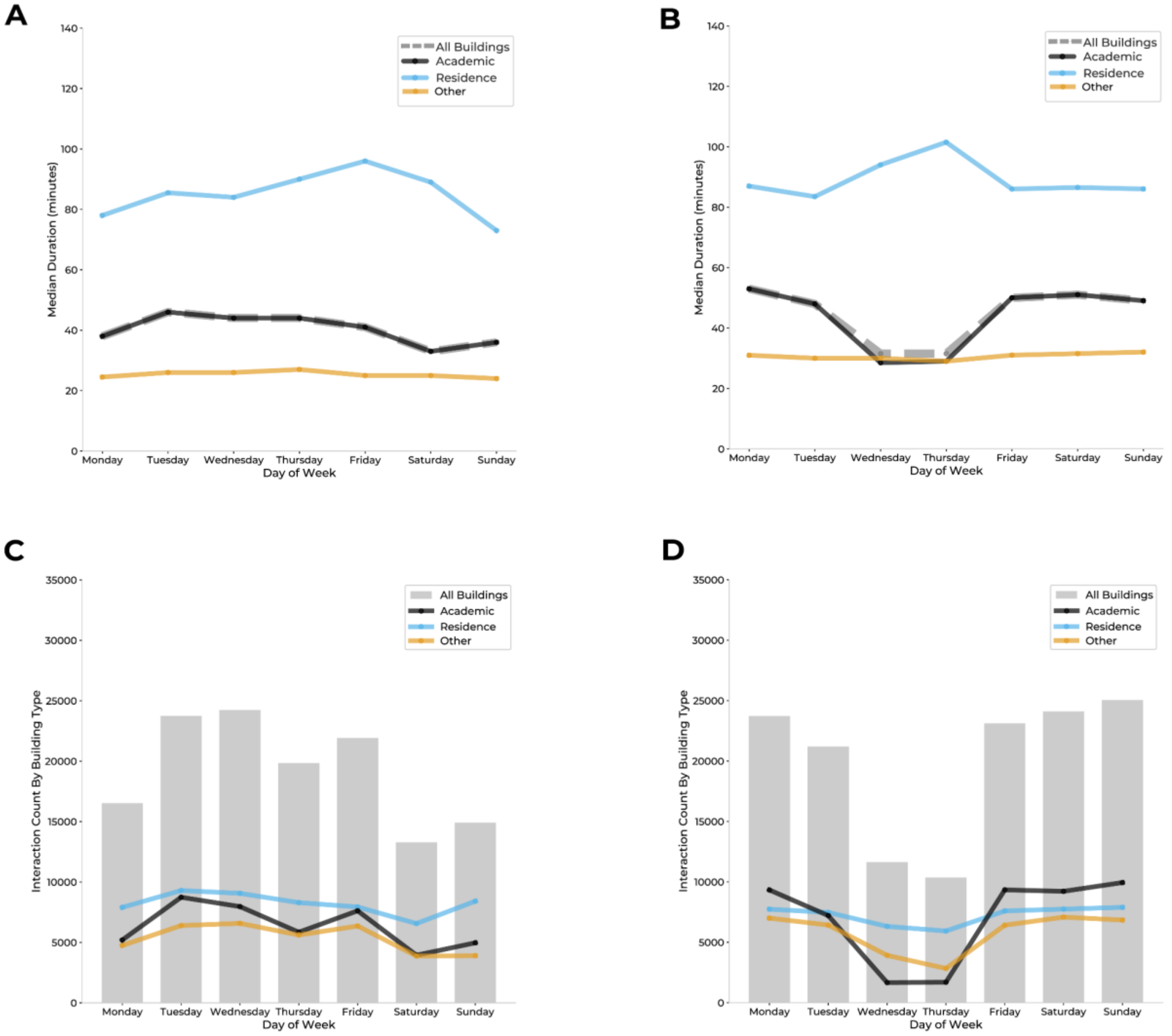

A-B. Median duration of access point (AP) connections in minutes, per day of the week and by building type (academic, residential, other, or all buildings) for Fall 2020 (**A**) and Spring 2021 (**B**). **C-D.** Daily number of AP connections, per day of the week and by building type (academic, residential, other, or all buildings) for Fall 2020 (**C**) and Spring 2021 (**D**).

Figure S7. Interaction patterns of individuals and pairs of individuals by testing status. Related to Figure 3.

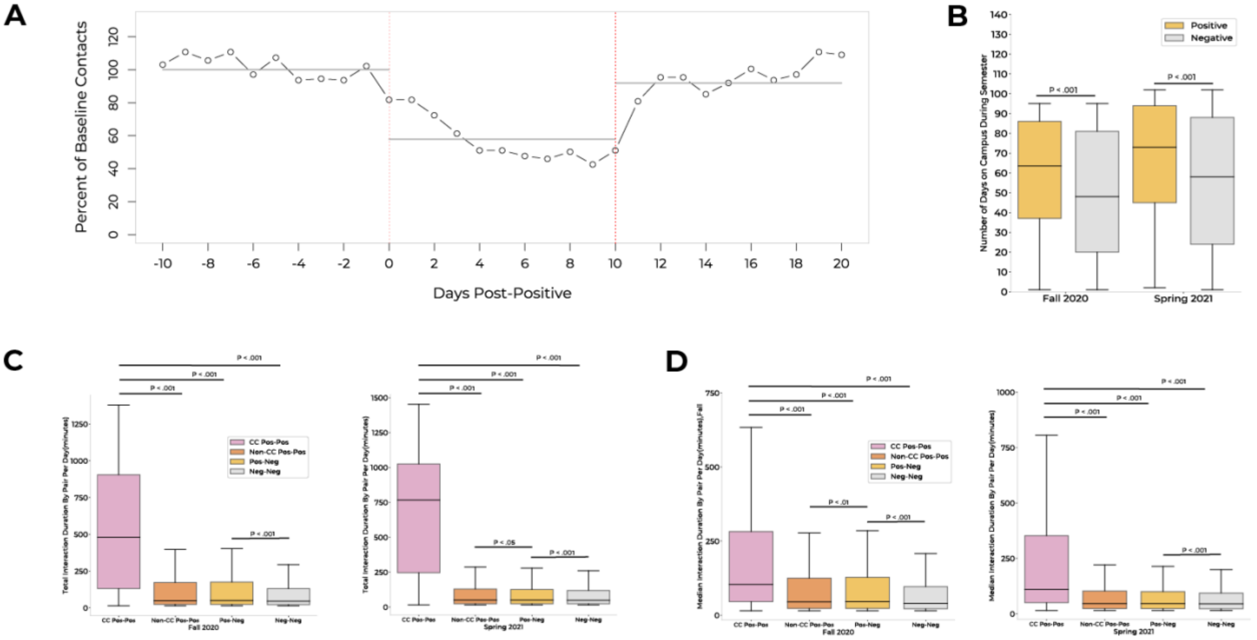

A. Percent of baseline WiFi contacts per day for the 30-day period surrounding the isolation period of positive cases. Day 0 was calculated as the earliest of symptom onset date and test date. Black lines indicate the median percent of baseline contacts (*y*) per individual for every day across the period (*x*). Gray bars indicate averages for three 10-day periods: pre-isolation (*i.e.*, baseline), isolation, and post-isolation. **B.** The number of days on campus for positive (orange) *vs.* negative (gray) individuals. **C.** Distributions of total pairwise daily interaction duration, for pairings categorized by testing status: CC Pos-Pos, pairs of pre-positive individuals with an association reported in manual contact tracing; Non-CC Pos-Pos, pairs of pre-positive individuals without an association reported in manual contact tracing; Pos-Neg, pairs of one pre-positive and one negative individual; Neg-Neg, pairs of two negative individuals. Pre-positive individuals were defined as students in the 10-day window prior to testing positive. **D.** Distributions of median interaction duration by pair per day, for pairings categorized by test status.

Figure S8. Attribute assortativity within the WiFi proximity network predicts daily case counts. Related to Figure 3.

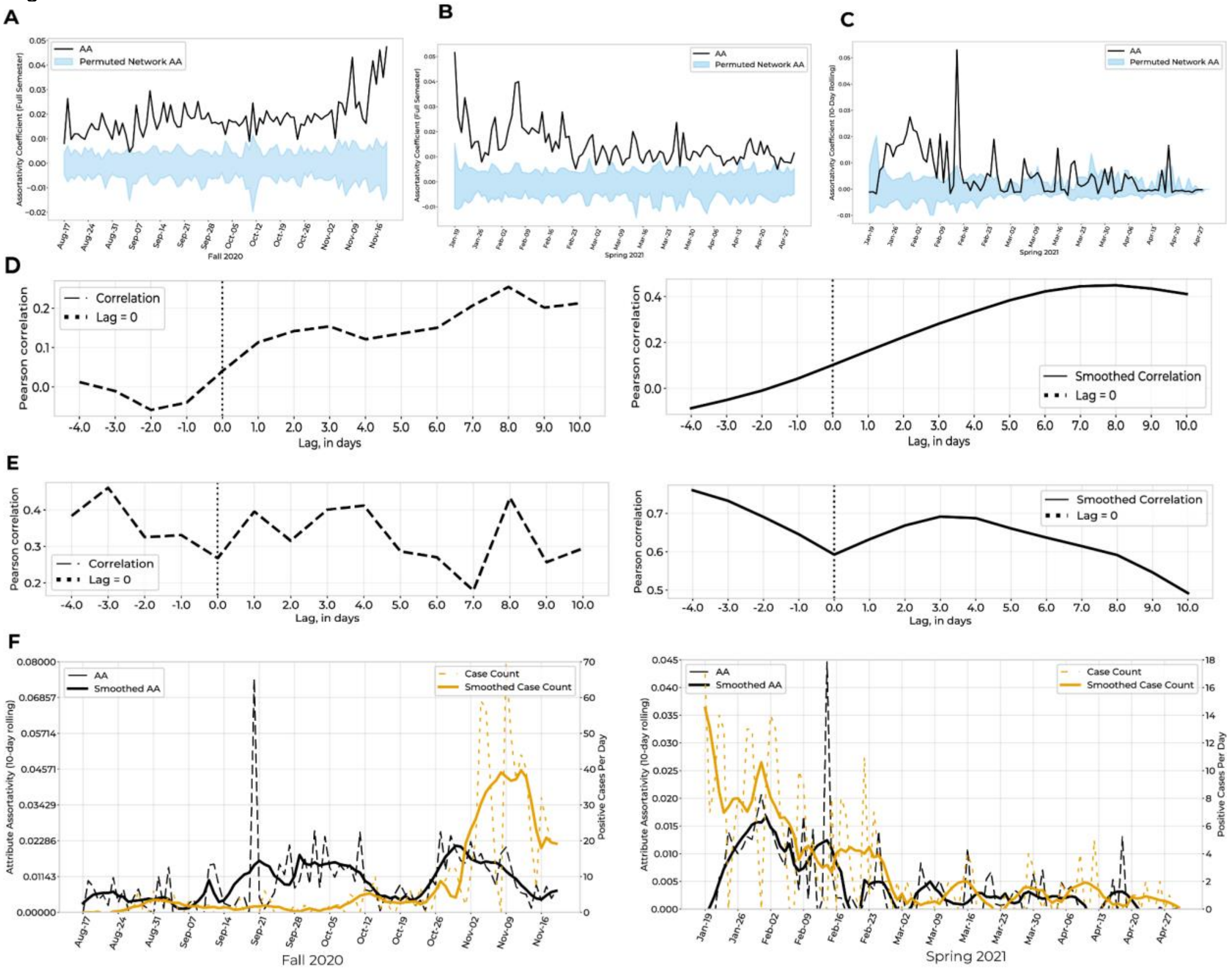

A-B. Comparison of attribute assortativity (AA) for positives *vs*. negatives, for Fall 2020 (**A**) and Spring 2021 (**B**). 95% Confidence Intervals (CI; blue) were calculated by permuting positive and negative labels for individuals within the proximity network each day. The AA of the proximity network (black) was above the upper bound of the CI for 98.9% (94/95 days) in the Fall 2020 semester and 98% (100/102 days) in the Spring 2021 semester, indicating significance with $p < 0.025$. **C.** We calculated the same metric with altered groupings: pre-positives (all individuals within a 10-day window prior to their positive test) *vs.* negatives (individuals not within a 10-day window prior to a positive test, regardless of overall semester testing status). The AA of the proximity network (black) was above the upper bound of the CI for 37.2% (38/102 days) in the Spring 2021 semester, indicating significance with $p < 0.025$. **D.** Correlations between daily AA (pre-positives *vs*. negatives) and daily case count with varying lag times, where both AA and case count are non-smoothed (top) or smoothed using Savitzky Golay filter (bottom), for Fall 2020. The highest correlation occurs with a lag time such that AA preceded the case count by 8 days (Pearson correlation = 0.253 for non-smoothed, 0.449 for smoothed). **E.** Cross-correlations between daily AA (pre-positives *vs*. negatives) and daily case count with varying lag times, where both AA and case count are non-smoothed (top) or smoothed using Savitzky Golay filter (bottom), for Spring 2021. The highest correlation occurs with lag time such that AA preceded the case count by 4 days (Pearson correlation=0.411 for non-smoothed; 3 days for smoothed, with correlation = 0.691). **F.** Unsmoothed case counts and unsmoothed AA *vs.* smoothed case counts and smoothed AA for Fall 2020 (left) and Spring 2021 (right).

Figure S9. Attribute assortativity within the WiFi proximity network when defining the “pre-positive” attribute as positive individuals missed by manual contact tracing. Related to Figure 3.

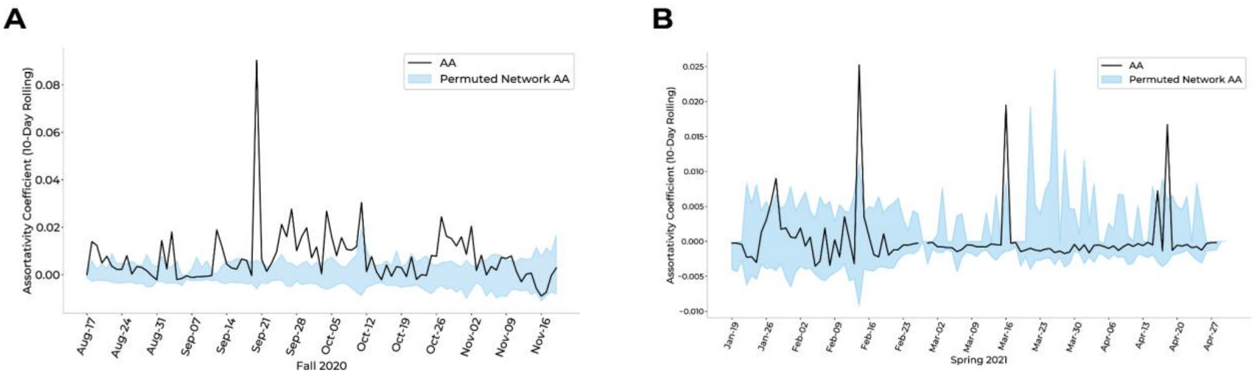

A-B. Comparison of attribute assortativity (AA) for pre-positives *vs*. negatives, for Fall 2020 (**A**) and Spring 2021 (**B**). 95% Confidence Intervals (CI; blue) were calculated by permuting pre-positive and negative labels for individuals within the proximity network each day. We calculated the same metric with pre-positives defined as individuals in the 10 days prior to their positive test missed by manual contact tracing, and negatives defined as individuals not in the 10 days prior to a positive test, regardless of overall semester testing status. The AA of the proximity network (black) was above the upper bound of the CI for 45.2% (43/95 days) of the Fall 2020 semester (**A**) and 8.8% (9/102 days) of the Spring 2021 semester (**B**), indicating significance with $p < 0.025$.

Figure S10. Sequenced wastewater samples are subject to increased degradation relative to clinical samples, but do not display wastewater-specific biases in coverage across the SARS-CoV-2 genome. Related to Figure 5.

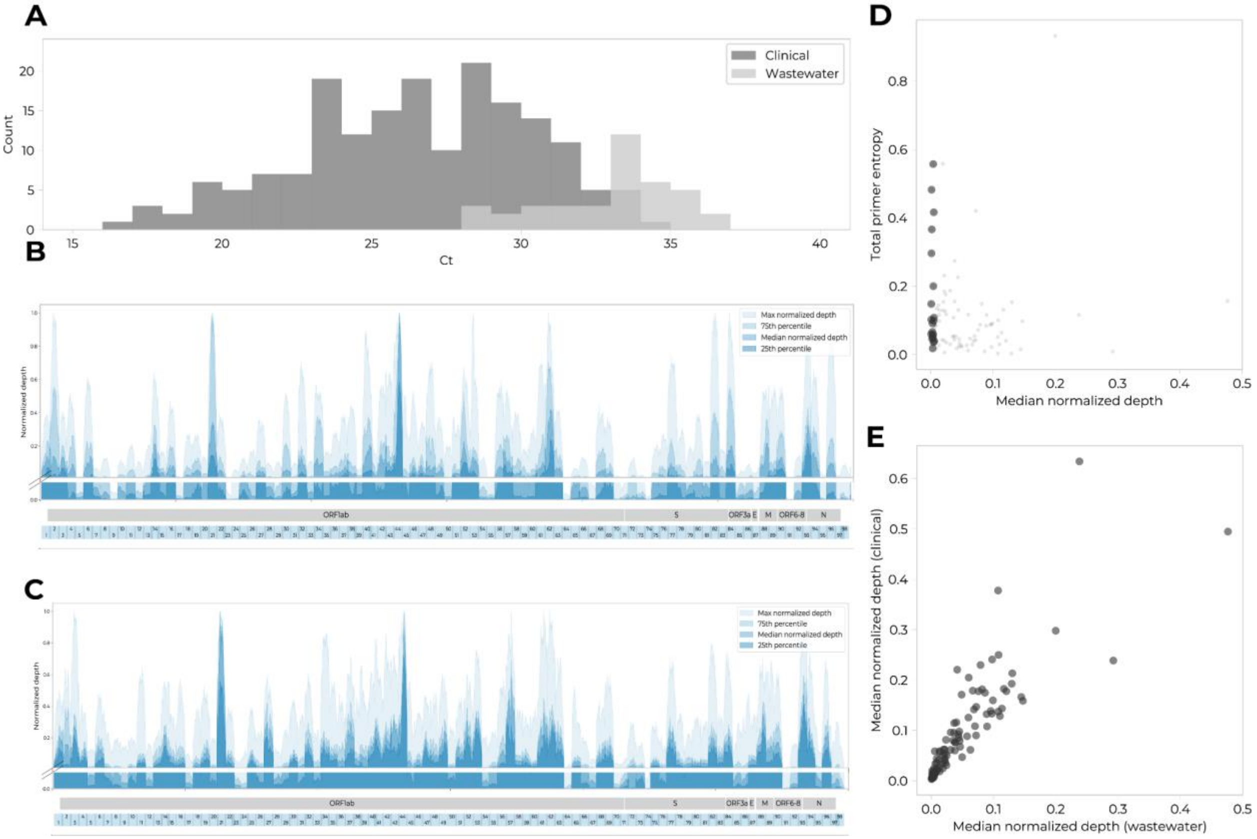

A. Cycle thresholds (Ct) for sequenced wastewater and clinical samples from Colorado Mesa University. **B-C.** Median normalized read depth per base, across all wastewater (**B**) and clinical (**C**) samples. The coloring indicates quartiles across all wastewater or clinical samples. Regions of lower depth in wastewater sequencing are of similarly low depth in clinical sequencing. **D.** Comparison of average amplicon read depth (*x* axis) and average amplicon primer entropy (*y* axis) for wastewater samples. We found no correlation between amplicon read depth and primer entropy (Pearson correlation = 0.02, p = 0.81). The 20 amplicons with lowest median normalized depth are shown as larger circles. **E.** Comparison of median normalized depth per amplicon between wastewater (*x* axis) and clinical (*y* axis) samples. There is a linear correlation between corresponding amplicons (Pearson correlation coefficient = 0.86; p < 0.001).

Figure S11. Presence of previously undetected mutations in sequenced wastewater samples suggests sequencing error. Related to Figure 5.

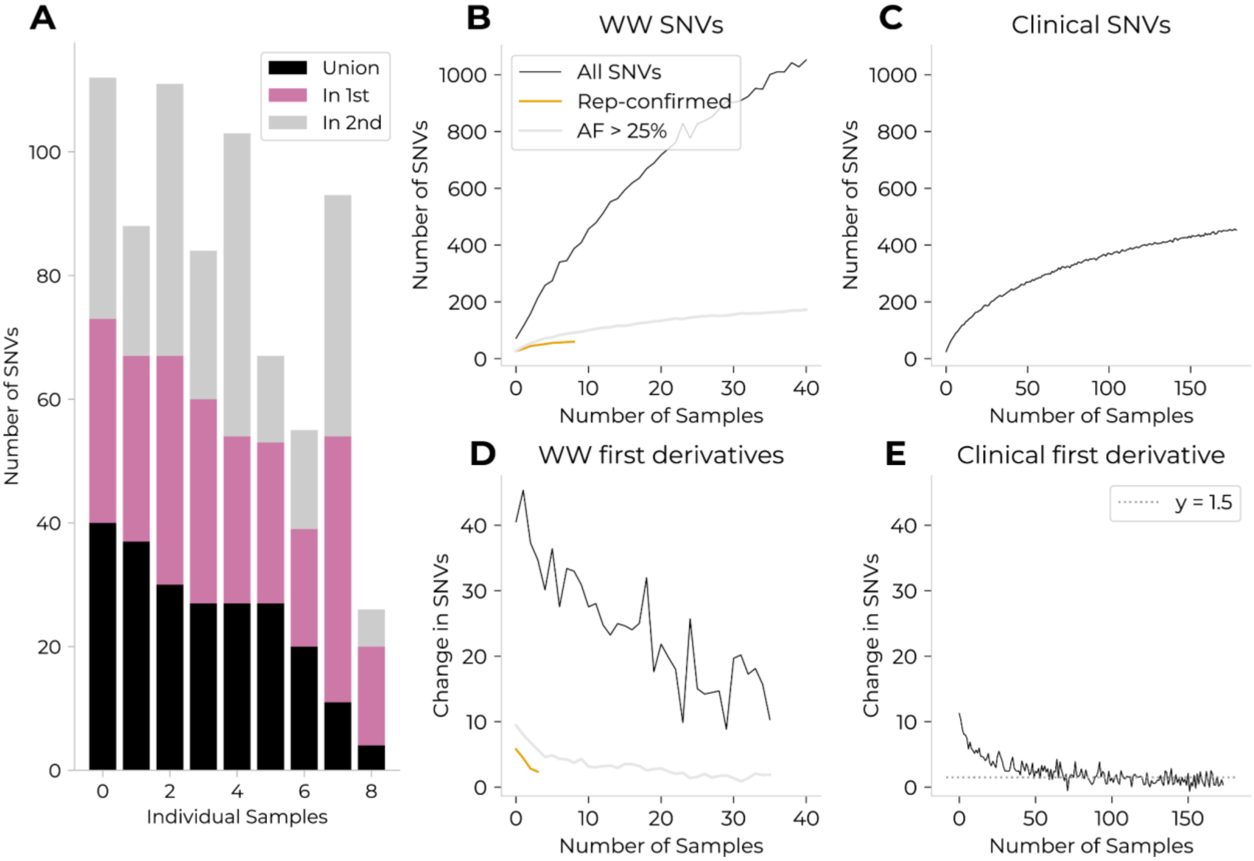

A. Distribution of single-nucleotide variants (SNVs) that were present in only one technical duplicate (pink and gray) or in both replicates (black). **B.** The number of unique SNVs expected for a given number of wastewater samples (see Methods). We additionally calculated the number of unique SNVs expected to pass an allele frequency quality control threshold of 25% (gray) or a technical replicate quality control (orange) for a given number of samples. **C**. The number of unique consensus-level SNVs expected for a given number of clinical samples. **D.** Smoothed (window=5) first derivative of unique wastewater SNVs per sample. Legend is the same as in panel (**B**). While the first derivatives of both replicate-confirmed SNVs (orange) and SNVs with an allele frequency greater than 25% (gray) approaches a rate of 1-3 new SNVs per sample, the first derivative for all SNVs of any allele frequency (black) approaches a rate of >10 new SNVs per sample. **E.** Smoothed (window=5) first derivative of unique clinical consensus-level SNVs per sample. The first derivative of all consensus-level SNVs seen in clinical samples approaches a rate of 1.5 new SNVs per sample. The comparison between (**D**) and (**E**) lends support to the hypothesis that most SNVs seen in wastewater samples are spurious, and that either confirming SNVs via technical replicates or a high allele frequency threshold is sufficient to remove the majority of spurious SNVs.

Figure S12. Social proximity patterns, transmission reconstruction networks, and experimental viral phenotypes associated with B.1.429.1. Related to Figure 6.

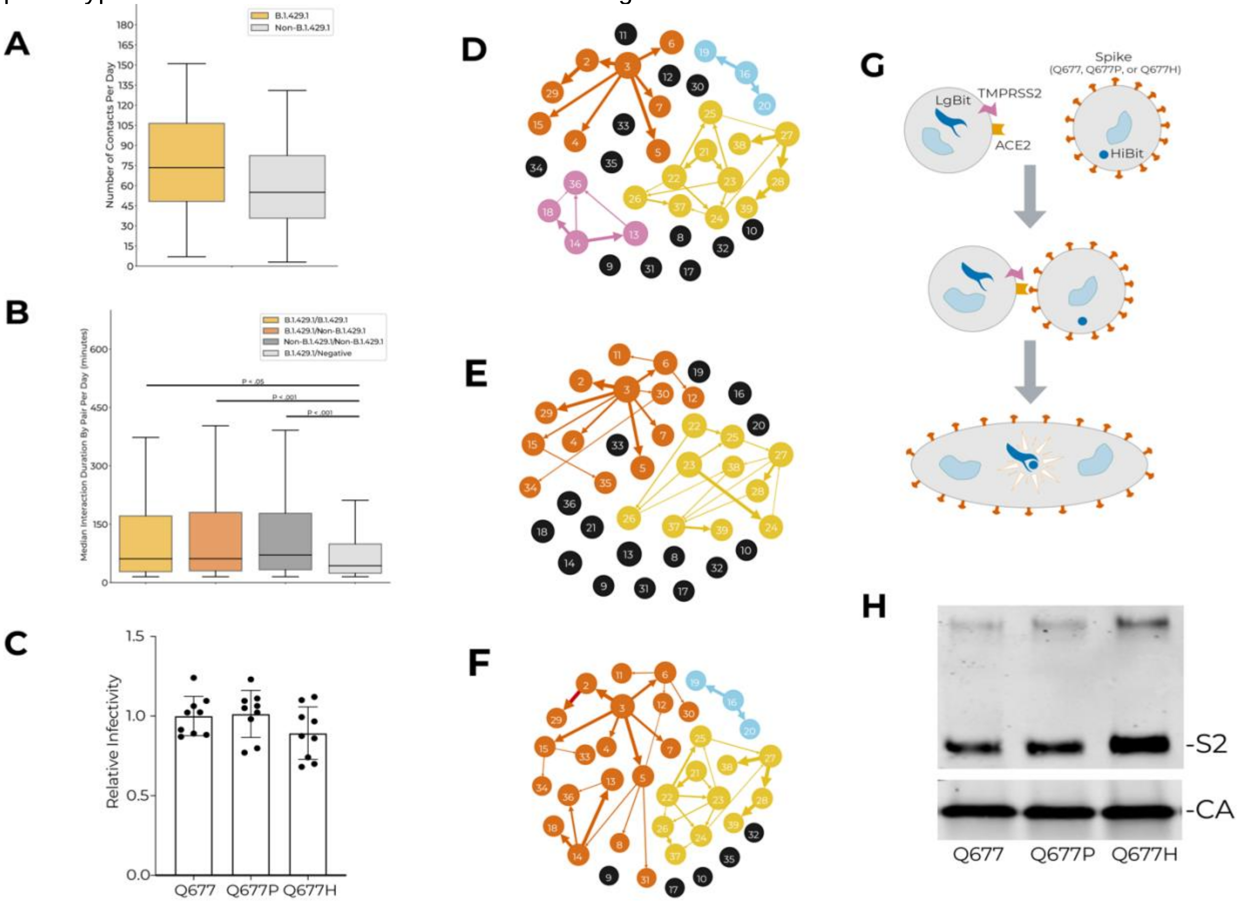

A. Average number of daily contacts per person in the 10 days prior to testing positive, for B.1.429.1-positive individuals *vs.* individuals contemporaneously infected with other lineages. p = 0.07. **B.** Pairwise median daily interaction duration for pairs of individuals with different viral lineages (see legend). Pairs of positive individuals interacted with one another in the 10 days prior to testing positive for longer durations than pairs of one B.1.429.1-positive and one negative individual. There were no other significant differences in pairwise interaction durations. **C.** Infectivity of viral pseudotypes with the S:Q677P or S:Q677H amino acid changes, relative to the ancestral allele. **D-F.** Transmission reconstruction networks for B.1.429.1 cases created with solely genomic information (**D**), solely manual contact tracing data (**E**), and both genomic information and WiFi-inferred 2-day contact data (**F**). **G.** The experimental strategy for assessing the fusogenicity of the S:Q677H or S:Q677P mutations relative to the ancestral residue, using two populations of cells: one expressing the modified Spike protein on the cell surface, and a second expressing the ACE2/TMPRSS2 receptors. The two populations were combined and fusion signal was assessed via HiBit-LgBit luminescent reporter. **H.** Western blot illustrating successful creation of viral pseudotypes bearing Spike protein with S:677Q, S:Q677H, and S:Q677P.
