# Supplementary material for: Multimodal surveillance of SARS-CoV-2 at a university enables development of a robust outbreak response framework
Source: Med. 2022 Dec 9;3(12):883–900.e13. doi: 10.1016/j.medj.2022.09.003 (PMC9482833; doi:10.1016/j.medj.2022.09.003)
Supplement: Data S1. Tables related to Figures 1, 2, 3, 4, 5, 6, and 7 and the STAR Methods [file mmc2.pdf]

Data S1. Tables related to Figures 1-7 and the STAR Methods.

Table S1. Properties of residence halls at Colorado Mesa University

Hall	Students	Percent Occupancy	Floors	Dining Hall Requirement	In-unit Bathroom	RAs	Square Footage	Ceiling Height	Volume per Person	Tests per Person	COVID-19 Cases
Hall A	160	0.89	3	1	1	5	46695	8	2334.75	5.09	43
Hall B	282	0.94	4	1	1	4	96429	8	2735.57	4.17	59
Hall C	251	0.88	4	1	1	8	80100	8	2552.99	4.31	52
Hall D	174	0.94	4	0	1	6	59360	8	2729.12	5.05	31
Hall E	99	0.83	3	0	1	4	28080	8	2269.09	5.63	19
Hall F	124	0.97	4	1	1	5	45996	9.67	3586.95	3.80	16
Hall G	396	0.91	4.25	1	0	15	93650	9.67	2286.86	3.81	46
Hall I	124	0.73	3	1	0	4	42507	8	2742.39	3.81	12
Hall L	187	0.93	3	1	0	6	42883	8	1834.57	4.14	31
Hall M	310	0.97	5	1	1	10	72500	8	1870.97	4.29	54
Hall N	157	0.79	3	1	0	6	44178	8	2251.11	4.37	16
Hall O	118	0.81	5	1	0	5	43843	8	2972.41	4.36	17

Structural and communal properties of residence halls. Volume per person was calculated as square footage * ceiling height / number of students per hall and serves as a proxy for air volume per person. Tests per person was calculated as the total number of COVID-19 tests conducted on hall residents over the academic year divided by the number of students in the hall. Hall G has 4 floors and a partial basement, covering approximately 25% of the square footage of the other floors.

Table S2. Properties of athletic teams at Colorado Mesa University

Team	Number of Students	Contact Level	Season	Location	Tests per Person	Number of COVID-19 Cases
Women's Golf	11	Low	both	outdoor	7.91	<5
Women's Club Volleyball	11	Low	both	indoor	NaN	<5
Men's Golf	12	Low	both	outdoor	5.42	<5
Women's Tennis	12	Low	both	outdoor	4.83	<5
Women's Cross Country	14	Low	fall	outdoor	4.43	<5
Men's Triathlon	18	Low	both	outdoor	NaN	<5
Cycling	65	Low	both	outdoor	NaN	<5
Women's Swimming	33	Low	both	indoor	7.48	6
Men's Tennis	10	Low	both	outdoor	6.20	7
Women's Triathlon	14	Low	both	outdoor	5.50	7
Cheerleading	30	Moderate	both	indoor	NaN	7
Men's Soccer	30	High	fall	outdoor	4.70	8
Sand Volleyball	19	Moderate	spring	outdoor	7.26	8
Women's Basketball	15	High	both	indoor	3.20	9
Women's Wrestling	22	High	both	indoor	6.73	9
Indoor Volleyball	20	Moderate	fall	indoor	5.95	9
Men's Track	55	Moderate	both	both	4.45	10
Men's Basketball	19	High	both	indoor	4.00	11
Men's Swimming	38	Low	both	indoor	4.34	11
Women's Lacrosse	30	Moderate	spring	outdoor	6.13	12
Women's Soccer	35	High	fall	outdoor	5.46	13
Women's Track	55	Moderate	both	both	3.62	13
Baseball	48	Moderate	spring	outdoor	3.63	14
Men's Lacrosse	43	Moderate	spring	outdoor	4.79	17
Men's Wrestling	40	High	both	indoor	4.10	23

Football	95	High	fall	outdoor	6.24	48

Number of students, contact level, season, location, tests per person, and number of COVID-19 cases per sports team. Tests per person was calculated as the total number of COVID-19 tests conducted on team members over the academic year divided by the number of students on the team; NaNs represent teams for which we lack testing data. Teams with fewer than 5 cases were labeled as “< 5” to minimize the risk of de-anonymity.

Table S3. Sports contact level definitions

Contact level	Definition	CMU teams
Low	Individual or small group sports where contact within six feet of other participants can be avoided. Sports that can be conducted with social distancing, consistent wearing of face coverings when within six feet of other people, or individually with no sharing of equipment or the ability to clean the equipment between use by competitors.	Cross country, cycling, golf, swimming, tennis, triathlon, volleyball (club)
Moderate	Team sports that can be played with only incidental or intermittent close contact between participants. This category may also include sports that involve close, sustained contact, but with protective equipment in place that may reduce the likelihood of respiratory particle transmission between participants OR sports where social distancing is possible but that use equipment that can't be cleaned between participants.	Baseball, cheerleading, lacrosse, track, volleyball (sand, indoor)
High	Team sports with frequent or sustained close contact (and in many cases, face-to-face contact) between participants and high probability that respiratory particles will be transmitted between participants.	Basketball, football, soccer, wrestling

Definitions of contact levels for sports teams. Sports teams were assigned a contact level of low, medium, or high based on variables that affect transmission risk, including physical proximity and the use of face coverings. Contact-level categories were created by synthesizing risk profiles defined by the California Department of Health's "Outdoor and Indoor Youth and Recreational Adult Sports" communication in April 2021 and the Colorado High School Activities Association 2020-2021 sports risk profiles.

Table S4. Chi-square analyses

**Variable**	**Chi-Square Statistic (df)**	**P-value (Uncorrected)**
Class years (Fall 2020)	95.67 (5)	4.31×10^{-19}
Class years (Spring 2021)	296.95 (5)	4.54×10^{-62}
Sports contact level	36.59 (2)	1.13×10^{-8}
Sports location (indoor *vs.* outdoor)	1.60 (1)	0.21
Sports season	10.22 (2)	0.006
Sports teams	75.08 (25)	6.60×10^{-7}
Residence halls	31.30 (11)	9.87×10^{-4}

Results of Pearson's chi-squared test for categorical variables assessed for heterogeneity in COVID-19 risk. Test statistics, degrees of freedom, and uncorrected p-values are reported.

Table S5. Published viral genomic data for clinical and environmental specimens

Sequence Identifier (Clinical Samples)	Biosample Accession	GenBank Accession	GISAID Identifier	SRA Accession
USA/CO-Broad-CMU_00017/2020	SAMN17211059	MW521521	EPI_ISL_872745	SRS8145178
USA/CO-Broad-CMU_00039/2020	SAMN17211081	MW521525	EPI_ISL_872749	SRS8145099
USA/CO-Broad-CMU_00043/2020	SAMN17211085	MW521526	EPI_ISL_872750	SRS8145173
USA/CO-Broad-CMU_00051/2020	SAMN17211093	MW521527	EPI_ISL_872751	SRS8145098
USA/CO-Broad-CMU_00054/2020	SAMN17211096	MW521528	EPI_ISL_872752	SRS8145097
USA/CO-Broad-CMU_00060/2020	SAMN17211102	MW521529	EPI_ISL_872753	SRS8145065
USA/CO-Broad-CMU_00062/2020	SAMN17211104	MW521530	EPI_ISL_872754	SRS8145064
USA/CO-Broad-CMU_00063/2020	SAMN17211105	MW521531	EPI_ISL_872755	SRS8145063
USA/CO-Broad-CMU_00064/2020	SAMN17211106	MW521532	EPI_ISL_872756	SRS8145095
USA/CO-Broad-CMU_00073/2020	SAMN17211115	MW521533	EPI_ISL_872757	SRS8145059
USA/CO-Broad-CMU_00075/2020	SAMN17211117	MW521534	EPI_ISL_872758	SRS8145057
USA/CO-Broad-CMU_00079/2020	SAMN17211121	MW521535	EPI_ISL_872759	SRS8145055
USA/CO-Broad-CMU_00080/2020	SAMN17211122	MW521536	EPI_ISL_872760	SRS8145054
USA/CO-Broad-CMU_00084/2020	SAMN17211126	MW521537	EPI_ISL_872761	SRS8145052
USA/CO-Broad-CMU_00088/2020	SAMN17211130	MW521539	EPI_ISL_872763	SRS8145050
USA/CO-Broad-CMU_00089/2020	SAMN17211131	MW521540	EPI_ISL_872764	SRS8145049
USA/CO-Broad-CMU_00090/2020	SAMN17211132	MW521541	EPI_ISL_872765	SRS8145048
USA/CO-Broad-CMU_00091/2020	SAMN17211133	MW521542	EPI_ISL_872766	SRS8145166
USA/CO-Broad-CMU_00092/2020	SAMN17211134	MW521543	EPI_ISL_872767	SRS8145165
USA/CO-Broad-CMU_00094/2020	SAMN17211136	MW521544	EPI_ISL_872768	SRS8145046
USA/CO-Broad-CMU_00098/2020	SAMN17211140	MW521545	EPI_ISL_872769	SRS8145162
USA/CO-Broad-CMU_00100/2020	SAMN17211142	MW521546	EPI_ISL_872770	SRS8145160
USA/CO-Broad-CMU_00101/2020	SAMN17211143	MW521547	EPI_ISL_872771	SRS8145159
USA/CO-Broad-CMU_00103/2020	SAMN17211145	MW521548	EPI_ISL_872772	SRS8145157
USA/CO-Broad-CMU_00004/2020	SAMN17210672	MW454484	EPI_ISL_765574	pending
USA/CO-Broad-CMU_00007/2020	SAMN17210675	MW454485	EPI_ISL_765575	pending
USA/CO-Broad-CMU_00010/2020	SAMN17210678	MW454486	EPI_ISL_765576	pending
USA/CO-Broad-CMU_00107/2020	SAMN17211149	MW521549	EPI_ISL_872773	SRS8145044
USA/CO-Broad-CMU_00108/2020	SAMN17211150	MW521550	EPI_ISL_872774	SRS8145043
USA/CO-Broad-CMU_00109/2020	SAMN17211151	MW521551	EPI_ISL_872775	SRS8145155
USA/CO-Broad-CMU_00110/2020	SAMN17211152	MW521552	EPI_ISL_872776	SRS8145154
USA/CO-Broad-CMU_00111/2020	SAMN17211153	MW521553	EPI_ISL_872777	SRS8145093
USA/CO-Broad-CMU_00113/2020	SAMN17211155	MW454487	EPI_ISL_765577	pending
USA/CO-Broad-CMU_00114/2020	SAMN17211156	MW454488	EPI_ISL_765578	pending
USA/CO-Broad-CMU_00116/2020	SAMN17211158	MW454489	EPI_ISL_765579	pending
USA/CO-Broad-CMU_00118/2020	SAMN17211160	MW454490	EPI_ISL_765580	pending
USA/CO-Broad-CMU_00123/2020	SAMN17211165	MW454492	EPI_ISL_765582	pending
USA/CO-Broad_WarriorLab-00125/2021	SAMN17906199	MW617630	EPI_ISL_1011598	SRS8270659
USA/CO-Broad_WarriorLab-00126/2021	SAMN17906200	MW617631	EPI_ISL_1011599	SRS8270657
USA/CO-Broad_WarriorLab-00128/2021	SAMN17906202	MW617632	EPI_ISL_1011600	SRS8270660
USA/CO-Broad_WarriorLab-00130/2021	SAMN17906204	MW617633	EPI_ISL_1011601	SRS8270663
USA/CO-Broad_WarriorLab-00131/2021	SAMN17906205	MW617634	EPI_ISL_1011602	SRS8270664
USA/CO-Broad_WarriorLab-00132/2021	SAMN17906206	MW617635	EPI_ISL_1011603	SRS8270666
USA/CO-Broad_WarriorLab-00133/2021	SAMN17906207	MW617636	EPI_ISL_1011604	SRS8270665
USA/CO-Broad_WarriorLab-00134/2021	SAMN17906208	MW617637	EPI_ISL_1011605	SRS8270667
USA/CO-Broad_WarriorLab-00135/2021	SAMN17906209	MW617638	EPI_ISL_1011606	SRS8270668
USA/CO-Broad_WarriorLab-00136/2021	SAMN17906210	MW617639	EPI_ISL_1011607	SRS8270669
USA/CO-Broad_WarriorLab-00137/2021	SAMN17906211	MW617640	EPI_ISL_1011608	SRS8270670
USA/CO-Broad_WarriorLab-00138/2021	SAMN17906212	MW617641	EPI_ISL_1011609	SRS8270671
USA/CO-Broad_WarriorLab-00140/2021	SAMN17906214	MW617642	EPI_ISL_1011610	SRS8270675
USA/CO-Broad_WarriorLab-00141/2021	SAMN17906215	MW617643	EPI_ISL_1011611	SRS8270674
USA/CO-Broad_WarriorLab-00142/2021	SAMN17906216	MW617644	EPI_ISL_1011612	SRS8270676
USA/CO-Broad_WarriorLab-00145/2021	SAMN17906219	MW617645	EPI_ISL_1011613	SRS8270679
USA/CO-Broad_WarriorLab-00146/2021	SAMN17906220	MW617646	EPI_ISL_1011614	SRS8270680
USA/CO-Broad_WarriorLab-00147/2021	SAMN17906221	MW617647	EPI_ISL_1011615	SRS8270681
USA/CO-Broad_WarriorLab-00148/2021	SAMN17906222	MW617648	EPI_ISL_1011616	SRS8270682
USA/CO-Broad_WarriorLab-00187/2021	SAMN17906261	MW617679	EPI_ISL_1011647	SRS8270725
USA/CO-Broad_WarriorLab-00188/2021	SAMN17906262	MW617680	EPI_ISL_1011648	SRS8270724
USA/CO-Broad_WarriorLab-00189/2021	SAMN17906263	MW617681	EPI_ISL_1011649	SRS8270726

USA/CO-Broad_WarriorLab-00190/2021	SAMN17906264	MW617682	EPI_ISL_1011650	SRS8270727
USA/CO-Broad_WarriorLab-00191/2021	SAMN17906265	MW617683	EPI_ISL_1011651	SRS8270729
USA/CO-Broad_WarriorLab-00192/2021	SAMN17906266	MW617684	EPI_ISL_1011652	SRS8270731
USA/CO-Broad_WarriorLab-00162/2021	SAMN17906236	MW617658	EPI_ISL_1011626	SRS8270697
USA/CO-Broad_WarriorLab-00177/2021	SAMN17906251	MW617670	EPI_ISL_1011638	SRS8270711
USA/CO-Broad_WarriorLab-00178/2021	SAMN17906252	MW617671	EPI_ISL_1011639	SRS8270714
USA/CO-Broad_WarriorLab-00182/2021	SAMN17906256	MW617674	EPI_ISL_1011642	SRS8270719
USA/CO-Broad_WarriorLab-00149/2021	SAMN17906223	MW617649	EPI_ISL_1011617	SRS8270684
USA/CO-Broad_WarriorLab-00150/2021	SAMN17906224	MW617650	EPI_ISL_1011618	SRS8270685
USA/CO-Broad_WarriorLab-00151/2021	SAMN17906225	MW617651	EPI_ISL_1011619	SRS8270686
USA/CO-Broad_WarriorLab-00152/2021	SAMN17906226	MW617652	EPI_ISL_1011620	SRS8270687
USA/CO-Broad_WarriorLab-00153/2021	SAMN17906227	MW617653	EPI_ISL_1011621	SRS8270688
USA/CO-Broad_WarriorLab-00156/2021	SAMN17906230	MW617654	EPI_ISL_1011622	SRS8270691
USA/CO-Broad_WarriorLab-00157/2021	SAMN17906231	MW617655	EPI_ISL_1011623	SRS8270689
USA/CO-Broad_WarriorLab-00158/2021	SAMN17906232	MW617656	EPI_ISL_1011624	SRS8270692
USA/CO-Broad_WarriorLab-00163/2021	SAMN17906237	MW617659	EPI_ISL_1011627	SRS8270698
USA/CO-Broad_WarriorLab-00167/2021	SAMN17906241	MW617661	EPI_ISL_1011629	SRS8270701
USA/CO-Broad_WarriorLab-00168/2021	SAMN17906242	MW617662	EPI_ISL_1011630	SRS8270703
USA/CO-Broad_WarriorLab-00160/2021	SAMN17906234	MW617657	EPI_ISL_1011625	SRS8270695
USA/CO-Broad_WarriorLab-00169/2021	SAMN17906243	MW617663	EPI_ISL_1011631	SRS8270702
USA/CO-Broad_WarriorLab-00172/2021	SAMN17906246	MW617665	EPI_ISL_1011633	SRS8270708
USA/CO-Broad_WarriorLab-00173/2021	SAMN17906247	MW617666	EPI_ISL_1011634	SRS8270709
USA/CO-Broad_WarriorLab-00176/2021	SAMN17906250	MW617669	EPI_ISL_1011637	SRS8270713
USA/CO-Broad_WarriorLab-00179/2021	SAMN17906253	MW617672	EPI_ISL_1011640	SRS8270715
USA/CO-Broad_WarriorLab-00181/2021	SAMN17906255	MW617673	EPI_ISL_1011641	SRS8270717
USA/CO-Broad_WarriorLab-00183/2021	SAMN17906257	MW617675	EPI_ISL_1011643	SRS8270720
USA/CO-Broad_WarriorLab-00184/2021	SAMN17906258	MW617676	EPI_ISL_1011644	SRS8270722
USA/CO-Broad_WarriorLab-00185/2021	SAMN17906259	MW617677	EPI_ISL_1011645	SRS8270721
USA/CO-Broad_WarriorLab-00186/2021	SAMN17906260	MW617678	EPI_ISL_1011646	SRS8270723
USA/CO-Broad_WarriorLab-00193/2021	SAMN17906267	MW617685	EPI_ISL_1011653	SRS8270730
USA/CO-Broad_WarriorLab-00194/2021	SAMN17906268	MW617686	EPI_ISL_1011654	SRS8270732
USA/CO-Broad_WarriorLab-00195/2021	SAMN17906269	MW617687	EPI_ISL_1011655	SRS8270733
USA/CO-Broad_WarriorLab-00196/2021	SAMN17906270	MW617688	EPI_ISL_1011656	SRS8270734
USA/CO-Broad_WarriorLab-00197/2021	SAMN17906271	MW617689	EPI_ISL_1011657	SRS8270735
USA/CO-Broad_WarriorLab-00198/2021	SAMN17906272	MW617690	EPI_ISL_1011658	SRS8270736
USA/CO-Broad_WarriorLab-00199/2021	SAMN17906273	MW617691	EPI_ISL_1011659	SRS8270737
USA/CO-Broad_WarriorLab-00200/2021	SAMN17906274	MW617692	EPI_ISL_1011660	SRS8270739
USA/CO-Broad_WarriorLab-00201/2021	SAMN17906275	MW617693	EPI_ISL_1011661	SRS8270740
USA/CO-Broad_WarriorLab-00202/2021	SAMN17906276	MW617694	EPI_ISL_1011662	SRS8270741
USA/CO-Broad_WarriorLab-00203/2021	SAMN17906277	MW617695	EPI_ISL_1011663	SRS8270742
USA/CO-Broad_WarriorLab-00204/2021	SAMN17906278	MW617696	EPI_ISL_1011664	SRS8270744
USA/CO-Broad_WarriorLab-00205/2021	SAMN17906279	MW617697	EPI_ISL_1011665	SRS8270743
USA/CO-Broad_WarriorLab-00206/2021	SAMN17906280	MW617698	EPI_ISL_1011666	SRS8270745
USA/CO-Broad_WarriorLab-00207/2021	SAMN17906281	MW617699	EPI_ISL_1011667	SRS8270746
USA/CO-Broad_WarriorLab-00208/2021	SAMN17906282	MW617700	EPI_ISL_1011668	SRS8270747
USA/CO-Broad_WarriorLab-00209/2021	SAMN17906283	MW617701	EPI_ISL_1011669	SRS8270748
USA/CO-Broad_WarriorLab-00210/2021	SAMN17906284	MW617702	EPI_ISL_1011670	SRS8270749
USA/CO-Broad_WarriorLab-00211/2021	SAMN17906285	MW617703	EPI_ISL_1011671	SRS8270750
USA/CO-Broad_WarriorLab-00212/2021	SAMN17906286	MW617704	EPI_ISL_1011672	SRS8270752
USA/CO-Broad_WarriorLab-00213/2021	SAMN17906287	MW617705	EPI_ISL_1011673	SRS8270753
USA/CO-Broad_WarriorLab-00215/2021	SAMN17906289	MW617706	EPI_ISL_1011674	SRS8270754
USA/CO-Broad_WarriorLab-00216/2021	SAMN17906290	MW617707	EPI_ISL_1011675	SRS8270755
USA/CO-Broad_WarriorLab-00217/2021	SAMN17906291	MW617708	EPI_ISL_1011676	SRS8270756
USA/CO-Broad_WarriorLab-00218/2021	SAMN17906292	MW617709	EPI_ISL_1011677	SRS8270758
USA/CO-Broad_WarriorLab-00219/2021	SAMN18306824	MW749874	EPI_ISL_1253885	SRS8468532
USA/CO-Broad_WarriorLab-00220/2021	SAMN18306825	MW749875	EPI_ISL_1253886	SRS8468533
USA/CO-Broad_WarriorLab-00221/2021	SAMN18306826	MW749876	EPI_ISL_1253887	SRS8468534
USA/CO-Broad_WarriorLab-00222/2021	SAMN18306827	MW749877	EPI_ISL_1253888	SRS8468535
USA/CO-Broad_WarriorLab-00225/2021	SAMN18306830	MW749878	EPI_ISL_1253889	SRS8468539
USA/CO-Broad_WarriorLab-00226/2021	SAMN18306831	MW749879	EPI_ISL_1253890	SRS8468540
USA/CO-Broad_WarriorLab-00227/2021	SAMN18306832	MW749880	EPI_ISL_1253891	SRS8468541
USA/CO-Broad_WarriorLab-00228/2021	SAMN18306833	MW749881	EPI_ISL_1253892	SRS8468542

USA/CO-Broad_WarriorLab-00229/2021	SAMN18306834	MW749882	EPI_ISL_1253893	SRS8468543
USA/CO-Broad_WarriorLab-00230/2021	SAMN18306835	MW749883	EPI_ISL_1253894	SRS8468544
USA/CO-Broad_WarriorLab-00231/2021	SAMN18306836	MW749884	EPI_ISL_1253895	SRS8468545
USA/CO-Broad_WarriorLab-00287/2021	SAMN18306892	MW749928	EPI_ISL_1253939	SRS8468340
USA/CO-Broad_WarriorLab-00288/2021	SAMN18306893	MW749929	EPI_ISL_1253940	SRS8468257
USA/CO-Broad_WarriorLab-00232/2021	SAMN18306837	MW749885	EPI_ISL_1253896	SRS8468546
USA/CO-Broad_WarriorLab-00233/2021	SAMN18306838	MW749886	EPI_ISL_1253897	SRS8468547
USA/CO-Broad_WarriorLab-00234/2021	SAMN18306839	MW749887	EPI_ISL_1253898	SRS8468548
USA/CO-Broad_WarriorLab-00235/2021	SAMN18306840	MW749888	EPI_ISL_1253899	SRS8468551
USA/CO-Broad_WarriorLab-00236/2021	SAMN18306841	MW749889	EPI_ISL_1253900	SRS8468552
USA/CO-Broad_WarriorLab-00237/2021	SAMN18306842	MW749890	EPI_ISL_1253901	SRS8468386
USA/CO-Broad_WarriorLab-00239/2021	SAMN18306844	MW749891	EPI_ISL_1253902	SRS8468388
USA/CO-Broad_WarriorLab-00241/2021	SAMN18306846	MW749892	EPI_ISL_1253903	SRS8468390
USA/CO-Broad_WarriorLab-00242/2021	SAMN18306847	MW749893	EPI_ISL_1253904	SRS8468391
USA/CO-Broad_WarriorLab-00243/2021	SAMN18306848	MW749894	EPI_ISL_1253905	SRS8468392
USA/CO-Broad_WarriorLab-00244/2021	SAMN18306849	MW749895	EPI_ISL_1253906	SRS8468393
USA/CO-Broad_WarriorLab-00245/2021	SAMN18306850	MW749896	EPI_ISL_1253907	SRS8468395
USA/CO-Broad_WarriorLab-00246/2021	SAMN18306851	MW749897	EPI_ISL_1253908	SRS8468396
USA/CO-Broad_WarriorLab-00248/2021	SAMN18306853	MW749898	EPI_ISL_1253909	SRS8468398
USA/CO-Broad_WarriorLab-00249/2021	SAMN18306854	MW749899	EPI_ISL_1253910	SRS8468399
USA/CO-Broad_WarriorLab-00250/2021	SAMN18306855	MW749900	EPI_ISL_1253911	SRS8468400
USA/CO-Broad_WarriorLab-00251/2021	SAMN18306856	MW749901	EPI_ISL_1253912	SRS8468401
USA/CO-Broad_WarriorLab-00252/2021	SAMN18306857	MW749902	EPI_ISL_1253913	SRS8468402
USA/CO-Broad_WarriorLab-00253/2021	SAMN18306858	MW749903	EPI_ISL_1253914	SRS8468403
USA/CO-Broad_WarriorLab-00256/2021	SAMN18306861	MW749904	EPI_ISL_1253915	SRS8468407
USA/CO-Broad_WarriorLab-00257/2021	SAMN18306862	MW749905	EPI_ISL_1253916	SRS8468408
USA/CO-Broad_WarriorLab-00258/2021	SAMN18306863	MW749906	EPI_ISL_1253917	SRS8468409
USA/CO-Broad_WarriorLab-00260/2021	SAMN18306865	MW749907	EPI_ISL_1253918	SRS8468411
USA/CO-Broad_WarriorLab-00261/2021	SAMN18306866	MW749908	EPI_ISL_1253919	SRS8468412
USA/CO-Broad_WarriorLab-00262/2021	SAMN18306867	MW749909	EPI_ISL_1253920	SRS8468413
USA/CO-Broad_WarriorLab-00263/2021	SAMN18306868	MW749910	EPI_ISL_1253921	SRS8468313
USA/CO-Broad_WarriorLab-00264/2021	SAMN18306869	MW749911	EPI_ISL_1253922	SRS8468314
USA/CO-Broad_WarriorLab-00265/2021	SAMN18306870	MW749912	EPI_ISL_1253923	SRS8468316
USA/CO-Broad_WarriorLab-00266/2021	SAMN18306871	MW749913	EPI_ISL_1253924	SRS8468317
USA/CO-Broad_WarriorLab-00267/2021	SAMN18306872	MW749914	EPI_ISL_1253925	SRS8468318
USA/CO-Broad_WarriorLab-00268/2021	SAMN18306873	MW749915	EPI_ISL_1253926	SRS8468319
USA/CO-Broad_WarriorLab-00269/2021	SAMN18306874	MW749916	EPI_ISL_1253927	SRS8468320
USA/CO-Broad_WarriorLab-00271/2021	SAMN18306876	MW749917	EPI_ISL_1253928	SRS8468322
USA/CO-Broad_WarriorLab-00272/2021	SAMN18306877	MW749918	EPI_ISL_1253929	SRS8468323
USA/CO-Broad_WarriorLab-00273/2021	SAMN18306878	MW749919	EPI_ISL_1253930	SRS8468324
USA/CO-Broad_WarriorLab-00275/2021	SAMN18306880	MW749920	EPI_ISL_1253931	SRS8468327
USA/CO-Broad_WarriorLab-00282/2021	SAMN18306887	MW749923	EPI_ISL_1253934	SRS8468334
USA/CO-Broad_WarriorLab-00276/2021	SAMN18306881	MW749921	EPI_ISL_1253932	SRS8468328
USA/CO-Broad_WarriorLab-00278/2021	SAMN18306883	MW749922	EPI_ISL_1253933	SRS8468330
USA/CO-Broad_WarriorLab-00283/2021	SAMN18306888	MW749924	EPI_ISL_1253935	SRS8468335
USA/CO-Broad_WarriorLab-00284/2021	SAMN18306889	MW749925	EPI_ISL_1253936	SRS8468336
USA/CO-Broad_WarriorLab-00285/2021	SAMN18306890	MW749926	EPI_ISL_1253937	SRS8468338
USA/CO-Broad_WarriorLab-00286/2021	SAMN18306891	MW749927	EPI_ISL_1253938	SRS8468339
USA/CO-CDCBI-Warrior_00289/2021	SAMN18498486	MW834877	EPI_ISL_1413769	SRS8613058
USA/CO-CDCBI-Warrior_00290/2021	SAMN18498487	MW834878	EPI_ISL_1413771	SRS8613060
USA/CO-CDCBI-Warrior_00291/2021	SAMN18498488	MW834879	EPI_ISL_1413774	SRS8613059
USA/CO-CDCBI-Warrior_00292/2021	SAMN18498489	MW834880	EPI_ISL_1413776	SRS8613061
USA/CO-CDCBI-Warrior_00293/2021	SAMN18498490	MW834881	EPI_ISL_1413779	SRS8613063
USA/CO-CDCBI-Warrior_00294/2021	SAMN18498491	MW834882	EPI_ISL_1413781	SRS8613064
USA/CO-CDCBI-Warrior_00295/2021	SAMN18498492	MW834883	EPI_ISL_1413784	SRS8613065
USA/CO-CDCBI-Warrior_00296/2021	SAMN18498493	MW834884	EPI_ISL_1413787	SRS8613066
USA/CO-CDCBI-Warrior_00300/2021	SAMN18790465	MZ217780	EPI_ISL_2133621	SRS8763083
USA/CO-CDCBI-Warrior_00301/2021	SAMN18790466	MZ217781	EPI_ISL_2133623	SRS8763082
USA/CO-CDCBI-Warrior_00302/2021	SAMN18790467	MZ217782	EPI_ISL_2133625	SRS8763084
USA/CO-CDCBI-Warrior_00304/2021	SAMN18790469	MZ217783	EPI_ISL_2133626	SRS8763087
USA/CO-CDCBI-Warrior_00305/2021	SAMN18790470	MZ217784	EPI_ISL_2133628	SRS8763088
USA/CO-CDCBI-Warrior_00312/2021	SAMN19224119	MZ217786	EPI_ISL_2133928	SRS9003810

USA/CO-CDCBI-Warrior_00311/2021	SAMN19224118	MZ217785	EPI_ISL_2133927	SRS9003809

**Sequence Identifier (Wastewater)**	**Biosample Accession**	**GenBank Accession**	**GISAID Identifier**	**SRA Accession**
USA-CO-Broad_CMU_W0011-2021	SAMN29048169			SRR19659512
USA-CO-Broad_CMU_W0012-2021	SAMN29048170			SRR19659511
USA-CO-Broad_CMU_W0013-2021	SAMN29048171			SRR19659500
USA-CO-Broad_CMU_W0014-2021	SAMN29048172			SRR19659489
USA-CO-Broad_CMU_W0015-2021	SAMN29048173			SRR19659478
USA-CO-Broad_CMU_W0016-2021	SAMN29048174			SRR19659475
USA-CO-Broad_CMU_W0017-2021	SAMN29048175			SRR19659474
USA-CO-Broad_CMU_W0018-2021	SAMN29048176			SRR19659473
USA-CO-Broad_CMU_W0019-2021	SAMN29048177			SRR19659472
USA-CO-Broad_CMU_W0020-2021	SAMN29048178			SRR19659471
USA-CO-Broad_CMU_W0021-2021	SAMN29048179			SRR19659510
USA-CO-Broad_CMU_W0030-2021	SAMN29048180			SRR19659509
USA-CO-Broad_CMU_W0034-2021	SAMN29048181			SRR19659508
USA-CO-Broad_CMU_W0040-2021	SAMN29048182			SRR19659507
USA-CO-Broad_CMU_W0043-2021	SAMN29048183			SRR19659506
USA-CO-Broad_CMU_W0082-2021	SAMN29048184			SRR19659505
USA-CO-Broad_CMU_W0084-2021	SAMN29048185			SRR19659504
USA-CO-Broad_CMU_W0094-2021	SAMN29048186			SRR19659503
USA-CO-Broad_CMU_W0095-2021	SAMN29048187			SRR19659502
USA-CO-Broad_CMU_W0109-2021	SAMN29048188			SRR19659501
USA-CO-Broad_CMU_W0130-2021	SAMN29048189			SRR19659499
USA-CO-Broad_CMU-W0031-2021	SAMN18858704			SRR19659498
USA-CO-Broad_CMU-W0033-2021	SAMN18858705			SRR19659497
USA-CO-Broad_CMU-W0035-2021	SAMN18858706			SRR19659496
USA-CO-Broad_CMU-W0038-2021	SAMN18858707			SRR19659495
USA-CO-Broad_CMU-W0039-2021	SAMN18858708			SRR19659494
USA-CO-Broad_CMU-W0042-2021	SAMN18858709			SRR19659493
USA-CO-Broad_CMU-W0044-2021	SAMN18858710			SRR19659492
USA-CO-Broad_CMU-W0045-2021	SAMN18858711			SRR19659491
USA-CO-Broad_CMU-W0046-2021	SAMN18858712			SRR19659490
USA-CO-Broad_CMU-W0047-2021	SAMN18858713			SRR19659488
USA-CO-Broad_CMU-W0053-2021	SAMN18858714			SRR19659487
USA-CO-Broad_CMU-W0054-2021	SAMN18858715			SRR19659486
USA-CO-Broad_CMU-W0058-2021	SAMN18858716			SRR19659485
USA-CO-Broad_CMU-W0059-2021	SAMN18858717			SRR19659484
USA-CO-Broad_CMU-W0062-2021	SAMN18858718			SRR19659483
USA-CO-Broad_CMU-W0069-2021	SAMN18858719			SRR19659482
USA-CO-Broad_CMU-W0070-2021	SAMN18858720			SRR19659481
USA-CO-Broad_CMU-W0101-2021	SAMN18858721			SRR19659480
USA-CO-Broad_CMU-W0107-2021	SAMN18858722			SRR19659479
USA-CO-Broad_CMU-W0108-2021	SAMN18858723			SRR19659477
USA-CO-Broad_CMU-W0127-2021	SAMN18858724			SRR19659476

Published sequences used in this study, listed with NCBI BioSample accessions, NCBI GenBank sequence accessions, GISAID identifiers, or SRA accessions.

Table S6. Lineages present in sequenced wastewater samples

Sample ID	Date	Site	B.1.1.519	B.1.126	B.1.2	B.1.234	B.1.350	B.1.429	B.1.429.1	B.1.533
**CO_CMU_W0011**	2-12	Site 5			0.09				0.84	
**CO_CMU_W0012**	2-12	Site 5			0.11				0.84	
**CO_CMU_W0013**	2-12	Site 5			0.12				0.82	
**CO_CMU_W0014**	2-16	Site 1		1.00						
**CO_CMU_W0015**	2-16	Site 8			0.12			0.11	0.77	
**CO_CMU_W0016**	2-17	Site 3							1.00	
**CO_CMU_W0017**	2-17	Site 6			0.91					
**CO_CMU_W0018**	2-17	Site 7			1.00					
**CO_CMU_W0019**	2-17	Site 11								
**CO_CMU_W0020**	2-17	Site 4							1.00	
**CO_CMU_W0021**	2-17	Site 2			0.93					
**CO_CMU_W0030**	2-11	Site 6			0.13	0.31			0.54	
**CO_CMU_W0034**	2-15	Site 5			0.28				0.47	
**CO_CMU_W0040**	2-18	Site 11			0.79					
**CO_CMU_W0043**	2-18	Site 8						0.16	0.81	
**CO_CMU_W0082**	3-01	Site 6							0.93	
**CO_CMU_W0084**	3-01	Site 4			0.99					
**CO_CMU_W0094**	3-04	Site 5			0.58				0.34	
**CO_CMU_W0095**	3-04	Site 3			0.49				0.26	
**CO_CMU_W0109**	3-09	Site 1							1.00	
**CO_CMU_W0130**	3-18	Site 1							0.31	
**CO_CMU_W0031**	2-11	Site 1					0.03		0.96	
**CO_CMU_W0033**	2-15	Site 10							0.99	
**CO_CMU_W0035**	2-18	Site 5			0.99					
**CO_CMU_W0038**	2-18	Site 4			0.99					
**CO_CMU_W0039**	2-18	Site 1			0.93				0.05	
**CO_CMU_W0042**	2-18	Site 10							0.99	
**CO_CMU_W0046**	2-22	Site 4			0.99					
**CO_CMU_W0058**	2-23	Site 11			0.98					
**CO_CMU_W0059**	2-23	Site 5							0.85	
**CO_CMU_W0062**	2-23	Site 1							1.00	
**CO_CMU_W0069**	2-25	Site 5			0.99					
**CO_CMU_W0070**	2-25	Site 3							0.96	
**CO_CMU_W0101**	3-08	Site 3							0.99	

**CO_CMU_W0107**	3-08	Site 5						0.94	
**CO_CMU_W0108**	3-09	Site 3						0.99	
**CO_CMU_W0127**	3-16	Site 5	0.12		0.08			0.72	0.07

Wastewater-detected lineages and their relative abundances. For each wastewater sample, collection date and site of collection are also listed. Lineages and abundances were determined via the Freyja program.

Table S7. Replicate-confirmed single nucleotide variant (SNV) mutations identified in sequenced wastewater samples but not present in sequenced clinical samples

**SNV**	**In CO**	**Change**	**Gene**	**Amino acid**	**Presence in proportion of 42 wastewater samples**	**Maximum allele frequency**
T5260A	False	Synonymous	ORF1ab	T1665T	10%	29%
T6174G	False	Missense	ORF1ab	I1970S	17%	32%
T9843A	False	Stop	ORF1ab	L3193*	14%	4%
C10650T	False	Missense	ORF1ab	T3462I	31%	100%
T27444G	False	Synonymous	ORF7a	L17L	7%	27%
C835T	True	Synonymous	ORF1ab	F190F	38%	100%
C6501T	True	Missense	ORF1ab	P2079L	38%	99%
G14126A	True	Missense	ORF1ab	S4621N	5%	100%
C21952T	True	Synonymous	S	V130V	38%	100%
T26099C	True	Missense	ORF3a	I236T	33%	100%
C28344T	True	Missense	N	T24I	38%	96%

Replicate-confirmed mutations identified in wastewater samples, but not in clinical samples. SNV nucleotide changes and positions are listed in bp, relative to the reference ancestral genome (NC_045512.2), along with corresponding amino acid changes, the mutation type, the proportion of samples bearing the mutation, and the highest allele frequency of that mutation across all samples. Some of these mutations were present in Colorado clinical viral genomes.

Table S8. Nucleotide and amino acid substitutions present in the B.1.429.1 lineage

**Region**	**Amino Acid Changes Characteristic of B.1.429**	**Additional Amino Acid Changes Characteristic of B.1.429.1**
ORF1a	T265I, I4205V	F2827L, V3367I
ORF1b	P314L, D1183Y	P314L, D1183Y
S	S13I, W152C*, L452R*, D614G*	Q677H
ORF3a	Q57H	A23V
N	T205I*	P142S, M234I*
ORF8		V100L

Characteristic amino acid mutations for B.1.429.1 and parent lineage B.1.429. Mutations are categorized by location. Mutations with an accompanying asterisk were not seen in all B.1.429.1 sequenced genomes; however, this may have been due to variable sequencing coverage.

Table S9. Jaccard similarity between clusters derived from genomic reconstruction supplemented by different contact tracing data sets

**Comparison Groups**	**Cluster Color**	**Jaccard Similarity**
**CT *vs.* 2-Day WiFi**	Orange	.421
	Light Blue	1.0
	Pink	0.0
	Yellow	1.0
**CT *vs.* 10-Day WiFi**	Orange	.889
	Light Blue	1.0
	Pink	1.0
	Yellow	1.0
**2-Day WiFi *vs.* 10-Day WiFi**	Orange	.474
	Light Blue	1.0
	Pink	0
	Yellow	1.0

We computed the Jaccard similarity across distinct transmission reconstruction networks created with individuals harboring the B.1.429.1 lineage. We supplemented the genomic data with three different definitions of close contacts: (1) manual contact tracing data (CT), (2) 10-day WiFi-derived contacts, and (3) 2-day WiFi-derived contacts.

Table S10. Summary of data types used at Colorado Mesa University, with additional recommendations for future surveillance programs

	**Epidemiological Analyses**	**Clinical Viral Genomic Sequencing**	**Wastewater Surveillance & Sequencing**	**WiFi Proximity Analyses**
**General Utility**	Identify epidemiological risk factors	Parametrize cluster distribution and identify lineages and mutations	Establish quality control standards	Discriminate interaction patterns by test positivity
**Current Utility to Institution**	Specify athletic, residential, and class-year risks	Identify VoC/VoI & transmission clusters	Designate testing resources based on surveillance; sequencing to detect mutations not seen clinically	———————
**Potential for Future Utility**	Automatically integrate metadata, wastewater surveillance, sequencing, and WiFi-based contact tracing into comprehensive surveillance system made directly accessible for the community or public health officials			
**Cost of Acquisition**	*Medium*: Personnel (cataloging information)	*Medium*: Transportation of excess clinical samples	*Medium–High*: Establishment of collection devices	*Low*: Established infrastructure
**Cost of Analysis**	*Low*: Personnel (analysis)	*High*: Sequencing reagents and computing resources	*Low*: PCR reagents<br>*Medium*: Sequencing costs reduced relative to clinical due to fewer samples	*Low*: Computing resources
**Difficulty of Analysis**	*Low*: Established statistical methods	*Low*: Established tools for phylogenetic trees	*Low*: Established tools for PCR analysis<br>*Medium*: Few established tools/methods for mixed sequence analysis	*High*: Few established tools

Comparison of the utility and cost of each data source used in this study. We recommend incorporation of these tools in a specific manner given resource availability (Figure 7). VoC = Variant of Concern; VOI = Variant of Interest.

Table S11. Summary of metrics used for the WiFi proximity network analyses

**Metric Name**	**Variables**	**Formula and Text Description**	**Figure Panel**
**Median Daily Contacts**	*individual*	$Median\left\{\begin{matrix}\text{for each day present on campus:}\\ \text{number of unique contacts for } \textit{individual}\end{matrix}\right\}$ <br>For each day that an individual is present on campus, calculate the number of unique contacts and take the median across all days.	Figure 3A, left
**Median Exposure Time per Contact**	*individual*	$Median\left\{\begin{matrix}\text{for each day present on campus:}\\ \dfrac{\sum_{\text{unique contacts}} \text{interaction time with } individual}{\text{number of unique contacts for } \textit{individual}}\end{matrix}\right\}$ <br>For each day than an individual is present on campus, divide the total time spent with all contacts by the number of unique contacts. Take the median of these values.	Figure 3A, right
**Number of Days on Campus**	*individual*	# of days that *individual* is connected to an AP <br>Calculate the number of days an individual is present on campus.	Supplemental Figure 5AB; Supplemental Figure 7B
**Number of Individuals on Campus**	*day, user category*	# of individuals of *user category* connected to an AP on *day* <br>For a given day, calculate the number of unique individuals of a given user category (i.e., students who tested positive for SARS-CoV-2) that are present on campus.	Supplemental Figure 5CD, left
**Proportion of Individuals on Campus**	*day, user category*	$\dfrac{\text{\# of individuals of } user\ category \text{ connected to an AP on } day}{Max\left\{\begin{matrix}\text{for each day:}\\ \text{\# of individuals of } \textit{user category} \text{ connected to an AP}\end{matrix}\right\}}$ <br>For a given day, calculate the number of unique individuals of a given user category (i.e., students who tested positive for SARS-CoV-2) present on campus. Scale the metric to the maximum value across all days.	Supplemental Figure 5CD, right
**Median AP Connection Duration**	*day of week, building category*	$Median\left\{\begin{matrix}\text{for each day} \in \textit{day of week}\text{:}\\ \text{for each AP connection} \in\ building\ category\text{:}\\ \text{duration of connection}\end{matrix}\right\}$ <br>For a given building category and a given day of the week (i.e., Mondays), calculate the median AP connection duration. Building categories include “all,” “residential,” “other,” and “academic.”	Supplemental Figure 6AB
**Median Daily AP Connections**	*day of week, building category*	$Median\left\{\begin{matrix}\text{for each day} \in \textit{day of week}\text{:}\\ \text{number of AP connections} \in \textit{building category}\end{matrix}\right\}$ <br>For a given building type and a given day of the week (i.e., Mondays), take the median of the number of AP connections. Building categories include “all,” “residential,” “other,” and “academic.”	Supplemental Figure 6CD
**Total Daily Interaction Duration**	*pair, day*	$\sum_{\text{all interactions of } \textit{pair} \text{ on } \textit{day}} \text{interaction duration}$ <br>Sum all interactions durations for a given pair on a given day. Pairs are defined as two individuals connected to the same AP at the same time.	Supplemental Figure 7C; Supplemental Figure 12A
**Median Daily Interaction Duration**	*pair, day*	$Median\left\{\begin{matrix}\text{for each interaction of } \textit{pair} \text{ on } \textit{day}\text{:}\\ \text{interaction duration}\end{matrix}\right\}$	Supplemental Figure 7D; Supplemental Figure 12B

		Take the median of all interaction durations for a given pair on a given day. Pairs are defined as two individuals connected to the same AP at the same time.	

The metric name, associated variables, formula, text description, and associated figure panel(s) are listed for all metrics used in WiFi proximity network analyses. AP, access point.
