# Supplementary material for: Multimodal surveillance of SARS-CoV-2 at a university enables development of a robust outbreak response framework
Source: Med. 2022 Dec 9;3(12):883–900.e13. doi: 10.1016/j.medj.2022.09.003 (PMC9482833; doi:10.1016/j.medj.2022.09.003)
Supplement: Methods S1. A templated version of the institutional review board study protocol, related to STAR Methods [file mmc3.pdf]

Methods S1. A templated version of the Institutional Review Board study protocol. Related to STAR Methods.

Purpose of the Study and Background

Purpose of the study: This study aims to help better detect SARS-CoV-2 [the virus that causes Coronavirus Disease 2019 (COVID-19)] and other disease-causing agents, by developing and implementing novel diagnostic and surveillance tests. This study also uses pathogen genome sequencing to aim to understand the spread of the virus/microbe between individuals and within and between groups of individuals that congregate and their surrounding community. By testing for and sequencing other microbes, we will also be able to identify illnesses that are co-occurring with COVID-19 and uncover potential emerging health crises. We will also integrate viral sequencing results with public health data and individuals' survey responses to better understand risk factors for disease and quantify the effectiveness of prevention and containment interventions.

Background: This study will occur during an unprecedented and rapidly expanding pandemic of COVID-19. Caused by the coronavirus SARS-CoV-2, COVID-19 has led to significant morbidity and mortality (estimated case-fatality ratio: 1.38%[1]). As of January 7, 2021, approximately 86 million people have been infected with SARS-CoV-2, including about 21 million in the United States[2]. Coronaviruses (family: Coronaviridae) are a family of single-stranded, positive-sense RNA viruses that infect the respiratory tract of animal and human hosts[3]. While many coronaviruses cause mild syndromes (e.g., the common cold), others can cause severe disease, including the Severe Acute Respiratory Syndrome (SARS), the Middle East Respiratory Syndrome (MERS), and COVID-19[4]. SARS-CoV-2 results in a wide range of clinical manifestations, including no symptoms[5], mild to moderate illness, and severe disease [e.g., acute respiratory distress syndrome (ARDS)][6], and disproportionately impacts vulnerable populations (e.g., the elderly and immunocompromised individuals). Infected individuals can shed virus whether asymptomatic, pre-symptomatic, or symptomatic[5,7], contributing to the rapid global spread.

Congregating settings such as schools, businesses, and community organizations are at particular risk of COVID-19 spread, including potential super spreader events. This study aims to help better detect SARS-CoV-2, to track the virus spread through viral genome sequencing, and to better understand risk factors for disease and quantify the effectiveness of prevention and containment interventions by examining public health data and individuals' survey responses.

Diagnostic and surveillance testing are critical to identify cases earlier to halt transmission events and enhance mitigation strategies. Early in the outbreak much of the testing to detect SARS-CoV-2 has been reverse transcriptase-polymerase chain reaction (RT-PCR) from nasal or nasopharyngeal swabs. Saliva samples are also of interest because they have a similar diagnostic sensitivity to nasopharyngeal swab and are more comfortable to collect[8]. The testing tools are still limited; according to a study done on patients in China during the peak of their outbreak in Spring 2020, nasopharyngeal RT-PCR test sensitivity was found to be 63%[9]. Furthermore, it can take several days to return results from RT-PCR tests[10]. For this reason, rapid antigen tests have become an additional important public health measure. However, low sensitivity and delayed results limits potential public health response within congregating settings and communities.

Additionally, because of the rapid, and often asymptomatic transmission of SARS-CoV-2, identifying transmission links between positive cases of COVID-19 using traditional epidemiological methods is challenging. Techniques like viral sequencing can both confirm the accuracy of and further elucidate the transmission links between cases and identify the origins of new introductions into congregating communities. Understanding the factors that affect transmission and disease burden in these populations can lead to implementation of public health measures in congregating settings.

Our first goal of the study is to improve, develop and implement novel diagnostic and surveillance tests. This will allow for increased testing within a community and facilitate identification of asymptomatic individuals with COVID-19. COVID-19 testing rates and capacity are essential to support a rapid public health response[8].

Our second goal of the study is to genome sequence SARS-CoV-2 positive samples from our study sites to understand transmission dynamics within these populations. Specifically, we aim to understand the spread of the virus between individuals and within and between groups of individuals that congregate and their surrounding community. By testing for and sequencing other microbes, in both SARS-CoV-2 positive and suspected samples, we will also be able to identify illnesses that are co-occurring with COVID-19 and uncover potential emerging health crises.

Our third goal of the study is to understand transmission and risk factors for disease through examining public health data paired with survey data that collects information on behaviors, demographics, symptoms, and contacts. With consent, this data can be linked to diagnostic and sequencing from our first two goals to gain a more detailed understanding of these factors.

Our fourth goal is to engage with congregate settings to understand community-wide transmission through contact data, genomic data, and epidemiology. Using clustering analyses, we will look at de-identified data from all individuals in a community to gain a more in-depth picture of disease spread.

Our work aims to support public health efforts for COVID-19 and other diseases. The study can leverage COVID-19 testing programs and surveillance that already exist within organizations and increase capabilities from current disease-tracing programs. Research participants are recruited at partnering sites, and their coded samples and data are sent to the central site for testing and analysis.

Criteria For Subject Selection

Number of subjects: A total of 20,000 subjects could be recruited into the study depending on the spread and need for outbreak investigation. However, the true number could be much smaller. Given the rapid nature of virus spread, we are listing here the larger potential number. As more study sites are added into this protocol, the number of study subjects may change based on the populations around this congregating setting.

Gender of subjects: All people, of all gender identities, will be allowed to participate in this study without any restrictions. Eligible people willing to participate in the study and opting to participate in research procedures will be allowed to do so.

Age of subjects: Participants must be 18 years or older. Minors (under 18 years of age) cannot participate in the study. Minors are not included because they currently represent a very small, or non-existent, population within the organizations involved within this study. Minors who belong to these organizations also are assumed to live away from their legal guardians, and thus would not readily be able to obtain a parent/guardian signature.

All study sites have an age of majority of 18 years of age. It should be noted that minors may incidentally be included in Part D, Clustering and Risk Analysis, because the age of individuals will often not be known.

Racial and ethnic origin: All people, of all racial and ethnic identities, will be allowed to participate in this study without any restrictions. Eligible people willing to participate in the study and opting to participate in research procedures will be allowed to do so, and thus we expect to have a racial/ethnic distribution within our study that reflects the racial/ethnic distribution of the congregating setting.

Inclusion criteria:

- Subjects should be at risk of SARS-CoV-2
- For Parts A-C only: subjects must have decision-making capacity to provide consent on their own behalf. To ensure that participants can properly understand the consent form and research aims:
    - Subjects must be able to speak English
    - Subjects must be able to consent for themselves, following the age of majority according to their state.
- Our study team will follow age of majority laws as previously described[11].

Exclusion criteria:

- Subjects outside of the United States who are not at risk of the relevant infectious disease.
- For Parts A-C only: minors (under the age of majority) cannot participate in the study. Minors are not included because they currently represent a very small, or non-existent, population within the organizations involved within this study. Minors who belong to these organizations also are assumed to live away from their legal guardians, and thus would not readily be able to obtain a parent/guardian signature.
- Subjects must be able to speak English. This requirement is in place to ensure that potential study participants can properly understand the consent form and research aims, and that they will be able to communicate with researchers.
- Study team members with access to patient identifiable information who have not signed an attestation form will not be permitted to join the study through Parts A-C.

Vulnerable subjects:
For all study parts*:* Pregnant women, students, and employees will be included within the study. Pregnant women will not be specifically targeted for recruitment, but because they are also at risk of contracting COVID-19, they are eligible to participate.

For Parts A-C: Students and employees will be recruited for Parts A-C from their universities or from other congregating settings if they are at least 18 years of age. They will be made aware through the consent form that they will not receive preferential treatment due to their involvement within the study, nor be penalized for non-participation. If study team members would like to participate in the study and have access to patient identifiable information, they will be required to sign an attestation form indicating their agreement to abide by measures designed to mitigate risks of conflict of interest, coercion, and misuse of data. This attestation will ensure that study staff are informed and aware of their own conflicts of interest arising from their dual roles within the study.

All participants will be informed that participation is completely voluntary, and they can withdraw or alter their participation at any time with no repercussions or consequences.

For Part D: Part D involves surveillance within a community using excess clinical samples and clustering data. As such, we will often not be able to distinguish who is a minor and who is not within our study. Although most individuals will be above 18, some students may be below this age and their samples/data may be incidentally included in our Part D datasets.

Methods and Procedures

This research proposal has four separate arms: (A) experimental viral/microbial diagnostic test development and implementation, (B) viral/microbial sequencing, (C) health information and survey data analysis, and (D) community clustering analysis.

This study will be organized according to study enrollment and sample collection sites. Our research team will partner with organizations/congregating settings to conduct Part D and to consent individual participants in Parts A-C.

Participating congregating settings (also referred to as “organizations” within this protocol) can choose to participate in any or all arms. For example, if organization X opts to allow their students/employees to opt into research arms A and B, these student/employees would only be offered a consent form with these two options. Please note that individual consent will not be collected for Part D, but work will only be done when organizations agree to participate in Part D. Students, staff, organization members, or members of the surrounding community may be enrolled into this study through a participating organization. Participation in this protocol will not affect an organization member or a community member’s relationship to the participating organization.

As COVID-19 has shown us, viruses can have tremendous reach. Thus, this project will involve both members of an organization, as well as people in the surrounding community. For example, University Z is providing COVID-19 tests to its own students and staff, in addition to people in the community. As such,

we will allow community members to join into this research protocol as well. Participation in this protocol will not affect an organization member or a community member's access to tests.

Lastly, outside of our work in congregating settings, researchers at the central site may also recruit individual participants around the U.S. to sign up for Parts A-C (providing samples and metadata for sequencing and diagnostic test development).

PART A - Viral/Microbial Test Development (Conducted exclusively for research purposes)
When a participant tests positive for SARS-CoV-2, has a suspected exposure, or is experiencing symptoms of COVID-19, they will have the opportunity to provide a sample for Part A. To do so, they can contact the study team. If enrolled through the central site, they can do so by filling out a survey (on a secure server) requesting a sample collection kit. If enrolled through a participating organization, they can do so by contacting their organizational study team using the email and phone number provided on their consent form (if the organizational study team doesn't reach out to them directly).

Participants who consent to Part A allow their rapid antigen test, nasal or nasopharyngeal swab and saliva samples to be used by researchers for development of diagnostic technology. Samples will be collected in the following ways:

From central site-enrolled participants:

1. Researchers at the central site will send a sample collection kit to research participants upon request. Participants will follow the included instructions, then mail their kits back to the central site for diagnostic testing.

From participants who belong to a participating organization or are in the surrounding community:

2. Researchers at the local participating organization will collect prospective samples, potentially of any type (including nasopharyngeal swabs, rapid antigen tests, and/or saliva samples) from consenting participants belonging to the organization or surrounding community.
3. Researchers can use any excess of their samples from their organization's clinical/surveillance testing for sequencing.
4. Researchers at the central site can use samples from excess biospecimen samples approved for use under other approved IRB protocols (see "Other Datasets").

Participants will have the option to provide a rapid antigen test, nasal, nasopharyngeal, and/or saliva sample to researchers to help them develop and implement tests to detect SARS-CoV-2 and other viruses/microbes. Participants can submit a sample regardless of whether they have been infected by SARS-CoV-2. By consenting to Part A, participants also are allowing researchers to use excess biospecimen samples, originally collected for the purpose of diagnostics or surveillance in their community, for the development of diagnostic tests. All biospecimen samples will be coded, labeled only with an ID number.

PART B - SARS-CoV-2 and Viral/Microbial Sequencing (Conducted exclusively for research purposes)
When a participant tests positive for SARS-CoV-2, has a suspected exposure, or is experiencing symptoms of COVID-19, they will have the opportunity to provide a sample for Part A. To do so, they can contact the study team. If enrolled through the central site, they can do so by filling out a survey (on a secure server) requesting a sample collection kit. If enrolled through a participating organization, they can do so by contacting their organizational study team using the email and phone number provided on their consent form (if the organizational study team doesn't reach out to them directly).

Participants who consent to Part B allow their samples to be used by researchers for sequencing SARS-CoV-2 and/or other viruses and microbes. Samples will be collected in the following ways:

From central site-enrolled participants:

1. Researchers at the central site will send a sample collection kit to research participants upon request. Participants will follow the included instructions, then mail their kits back to the central site for sequencing.

From participants who belong to a participating organization or are in the surrounding community:

1. Researchers at the local participating organization will collect prospective nasal swabs, potentially of any type (including nasopharyngeal swabs), rapid antigen tests, and/or saliva samples from consenting participants.
2. Researchers can use any excess of their samples from their organization's clinical/surveillance testing for sequencing.
3. Researchers at the central site can use sequencing data from excess biospecimen samples approved for use under other approved IRB protocols (see "Other Datasets").

The purpose of this research is to identify circulating viral strains and their prevalence in the community. Further research on this data will help researchers carry out cluster investigations, understand connections between cases, identify sources of infection, and assess the impact of interventions. Sequencing may also be conducted for other viruses and microbes to look for potential emerging health issues within the community. Sequencing other pathogens is also helpful for researchers to understand what diseases co-occur with COVID-19.

Other Datasets. If available, we will compare excess biospecimen sample data from other approved IRB protocols or through de-identified excess samples sequenced through Research Arm D. For example, de-identified excess samples from University Z may have already been sequenced for the purpose of public health monitoring. Study of excess biospecimen samples increases the pool of samples our researchers can use and will help us gain a better understanding of the spread of different SARS-CoV-2 viral strains within the community over time. This comparison is incredibly important for understanding earlier time points in the pandemic.

Therefore, we intend to link the sequencing data generated from an existing protocol operationalized by the central site to any data a participant provides in this study (including any biospecimen samples submitted under this protocol, survey response data, other data provided via the Fathom dashboard under Part C, and experimental diagnostic test results). We will seek approval under the respective IRB board for an amendment to allow us to re-identify biospecimen samples from the protocol for the purpose of linking data to this study.

PART C - Health Information and Survey Response Data Analysis (Conducted exclusively for research purposes)

For all participants:

The central site may circulate surveys to research participants to ask about topics such as pre-existing conditions, vaccination status, vaccination type, vaccination date, health behaviors, and more. Designated researchers with access to participant contact information will send surveys over a secure server (such as RedCap or Qualtrics). All surveys distributed by the central site will undergo IRB approval before being incorporated. Survey questions are available for review in the supplemental attachment "Survey Questions."

For participants belonging to a participating organization or the surrounding community:

Most participating organizations will already collect health information as part of their routine health and outbreak surveillance. If an organization permits, participants have the option to allow their organizations to share this health information, including diagnostic test results, symptom attestation and contact tracing data related to disease outbreaks, with study staff. For University Z, two surveys are currently conducted. Further information about these two surveys is provided below:

Routine surveillance survey and health information:

Participants can consent to share their responses to public health surveillance questions collected by their organizations. For example, University Z distributes a daily symptom survey on a web-based application. This app was developed by the company Fathom and allows for members of the community to manage their health through the COVID-19 pandemic. Questions covered within this data set could include symptoms in the past 24 hours, COVID-19 vaccination status, and whether the participant has had potential contact with a COVID-19 positive person within the past 14 days.

As part of the public health response in these communities, individuals will also be sharing information from routine COVID-19 testing and surveillance conducted by the organization. For instance, the Fathom database helps University Z keep track of COVID-19 diagnostic test results, vaccination scheduling, and contacts. By opting into Part C, individuals also are opting to share this data with researchers for analysis.

If the individual and organization both agree to share contact information with our study, they will do so in a manner that is de-identified (example information, individual #1 is 1 degree of separation from individual #4).

COVID-19-positive surveillance survey:
Some organizations will ask individuals to complete a survey when they test positive for COVID-19. This additional survey and its distribution may vary by organization. Organizations may distribute the survey online or through a secure app. Any data collected through these surveys will be hosted on secure databases, such as Qualtrics or Fathom. In general, this survey will include, but is not limited to symptoms, age, sex, race, pre-existing conditions, COVID-19 vaccination status, and housing arrangements.

<u>Part D - Genomic Epidemiology and Community Clustering Analysis (conducted exclusively for research purposes)</u>
When studying outbreak dynamics, it is important to examine the role of importation and transmission patterns in propagating epidemic spread. For example, case clusters and superspreading events may be major drivers of transmission but contact tracing from such events can be intensive and limited in scope. By performing an analysis based on genomic epidemiology, we can better understand the spread of disease in a community setting to prioritize public health interventions. Conducting these analyses can help distinguish between different forms of community spread that occur when individuals are in proximity by chance.

This research section is separate from Part A, B, and C in that we will be collecting Part D data from participating organizations (added via protocol amendment once they have been identified) rather than from specific individuals. Part D data will be collected under a waiver of consent. Potentially all individuals and/or community members may be included in genomic epidemiology and clustering analyses, even if they have not consented directly into the study in Parts A-C.

Secondary-use sample viral/microbial sequencing:
It is important to catalog viral/microbial diversity within affected regions as well as within individual patients. This knowledge can yield crucial insight into the origin and evolution of an epidemic, transmission dynamics, evolutionary rates, and any potential mutations that may influence diagnostics and prevention strategies. We intend to partner with communities to help them sequence coded secondary-use samples and environmental samples such as wastewater. Participating organizations, research collaborators, or their partner diagnostic labs would send coded secondary-use samples to the central site for viral/microbial sequencing. Although our partners may hold a code for the samples for public health and surveillance purposes or work under separate IRB-approved protocols, this code will not be disclosed to central site researchers.

Samples may be sent with metadata including date of collection. Secondary use samples will be processed through the same techniques as Samples in Part B. All human results will be securely destroyed before the analysis phase; only viral and microbial genomes will be analyzed.

Case metadata:
Participating organizations may provide metadata to help identify potential disease clusters and similarities in disease severity. This metadata may be analyzed in conjunction with sequencing data from secondary-use samples provided by a participating organization. Such metadata includes, but is not limited to:

1. Participant symptoms
2. Participant sex/gender

3. Grouping data. Grouping data would indicate whether two individuals have had contact with each other. This information will be used by central site researchers to gauge the effectiveness of a participating organization or community settings' health response. For example, University Z collects public health data on students, and could provide information on the individual's class year (if applicable), coded class schedule (e.g., "in Class A") and university group or extracurricular group participation (e.g, "in Group A"). Central site researchers may receive sports team type (e.g., soccer, football, lacrosse) and other group names for purposes of identifying high-risk activities within community settings and highlighting these activities for other institutions as potentially risky activities. A participating organization may also share dates of large group events, as these are relevant to understanding potential superspreader events.
4. Group statistics. This includes but is not limited to: number of positive cases per group, total number of individuals per group, meeting frequency and average meeting duration, and whether precautions were taken during group meetings (such as meeting outside or wearing masks).
5. Deidentified contact links also may be made between individuals based on participant-identified contact data or through geolocation data (described below in "Geolocation Contact Data and Risk Estimation"). As an example of participant-identified contact data, University Z collects contact data through a HIPAA-compliant web-app, and may share that individual X had contact with individual Y.

Organizations may provide clustering data for the purpose of improving public health surveillance at their institution. Select appointed individuals may have access to a code key to match codes to actual groups or individuals and will control access to that code based on their own organizational source documentation and policies. Code keys will never be distributed to central site researchers. Researchers may publish the metadata listed above along with sequencing data, to communicate potential high-risk groups in other communities.

Geolocation contact data and risk estimation:
We are using genomic data from positive cases to group contemporaneous cases by genotype, further partitioning cases into clusters. Where derived differences exist between genomes, phylogenetic analysis can be used to identify the directionality of transmission between individuals and clusters of cases. This is especially limited by the relatively low substitution rate of SARS-CoV-2; many co-occurring cases have verbatim-identical genomes, making it ambiguous who infected whom. In the dynamic community, traditional contact tracing does not provide the granularity of spatial or temporal localization to identify all individuals who were in physical proximity that may have plausibly led to transmission.

To improve resolution of clusters or otherwise ambiguous transmission chains, and to evaluate risk of infection, participating organizations may provide central site researchers with secondary-use geolocation data. This geolocation data could be in the form of datasets such as WiFi connectivity or badge swipes. To provide for a control group and basis for comparison when considering aggregate epidemiological statistics, we will use proximity data for all individuals within the dataset, not just positive cases.

In the case of University Z, the university has engaged Degree Analytics to aggregate metrics from the university-controlled Wi-Fi network to track metrics in support of student success, operations, and security on campus. This WiFi data can indicate whether individual X and Y were at the same Wi-Fi access point and specifies their duration of contact. The Wi-Fi dataset as used internally by University Z currently makes use of identifiers; for our analysis we request the use of a version of the dataset that is de-identified: individual identifiers would be coded to mask identity, and Wi-Fi access point IDs would be hashed so their specific location is not known. The dataset would only show pairs of coded individuals who were in proximity, when, and for how long. Relating coded identifiers to clinical specimens (i.e., viral genomes) will be done by University Z and Degree Analytics. This data is important because it can vastly improve our ability to estimate risk on campus and to resolve transmission chains. Central site researchers will not make attempts to identify individuals based on geolocation data.

Wastewater surveillance and sequencing:

Research suggests SARS-CoV-2 can be detected in wastewater approximately one week prior to symptom onset of COVID-19. Therefore, infectious disease surveillance in wastewater from congregate settings such as dormitories, apartment buildings, or neighborhoods can be a cost-effective mechanism of early detection of an outbreak. Furthermore, viral genomic sequencing of infectious diseases from wastewater provides insight into the diversity of viral strains and co-circulation of other microbes, and may contribute to public health interventions to prevent the spread of COVID-19 or other diseases in our communities. Residual wastewater specimens will be shipped to the central site for viral/microbial genome sequencing. Only microbial genomic information will be stored, sequenced, and analyzed. Within the microbial analysis of wastewater, there is no identifiable health information connected to any specific individual. Therefore, no protected health information will be accessed by either the organization or the study researchers.

Researchers will compare sequencing data from excess clinical data, collected nasal/saliva swabs, and wastewater surveillance samples. By comparing the coded locations where wastewater samples were collected to the coded locations of positive cases, we will attempt to uncover the burden of disease within a participating organization. Comparing each dataset is also useful to understanding the usefulness of wastewater collection as a surveillance method. Data concerning the viral strains of disease within the wastewater will be shared back to the organization donating that information (e.g., University Z). The data generated from wastewater sample analysis may also be given to state and local health departments collaborating with the organization. Viral strain will be linked to the location of sample collection, meaning that the data will correspond to specific geographic locations. Locations will be coded for the purpose of our analysis, but a code key will be maintained by the participating organization, Fathom, or a central site study manager. Since this is aggregate data, there will be no information traceable to a specific individual within the organization. However, some detected sequences in wastewater may be similar to sequences from clinical samples. Data may be reported in an academic paper put forward by researchers at the central site.

Data Analysis

Part A analysis:

The scope of this research will extend beyond qPCR-based diagnostics to include a new method: CRISPR-based diagnostics. The discovery of the RNA-targeting CRISPR effector protein Cas13a (formerly known as C2c2), and development of Specific High-sensitivity Enzymatic Reporter unLOCKing (SHERLOCK), a Cas13a-based diagnostic technology, has allowed for the development of Cas13a-based viral diagnostics. The SHERLOCK diagnostic technology involves two steps: 1) an isothermal amplification step with primers designed to the virus of interest, where the reaction input is either RNA or DNA and 2) a detection step where the amplified product is detected by Cas13a using a virus-specific guide sequence. This detection event triggers the cutting of a fluorescent reporter molecule that can be measured on a fluorescent plate reader, or a colorimetric reporter using lateral flow technology.

We will also test samples using a scalable, multiplexed pathogen detection platform called Combinatorial Arrayed Reactions for Multiplexed Evaluation of Nucleic acids (CARMEN). In the CARMEN platform, nanoliter droplets containing CRISPR-based nucleic acid detection reagents self-organize in a microwell array to pair with droplets of amplified samples, testing each sample against each CRISPR RNA (crRNA) in replicate. The combination of CARMEN and Cas13 detection (CARMEN-Cas13) enables robust testing of >4,000 crRNA-target pairs on a single array. It can sensitively detect and differentiate between viral sequences at the species, strain/subtype, and SNP levels. The development and validation of this platform for 169 human-associated viruses has been previously described[12]. These CRISPR-based technologies will be leveraged to detect the presence of SARS-CoV-2 or other respiratory pathogen RNA within the suspected or laboratory-confirmed samples. A colorimetric output may also be used to assess these samples, in addition to the fluorescence detection step described above.

Many samples will be analyzed using SHERLOCK, CARMEN, and other diagnostic tests at the central site. Some samples will be analyzed using SHERLOCK in comparison with RT-qPCR (the gold standard), CARMEN, and with other point-of-care diagnostic methods.

Part B bioinformatics and Part D secondary-use sample sequencing analysis:

Sequencing: The central site has determined optimal viral sequencing protocols for SARS-CoV-2-positive clinical samples collected[13]. This currently involves Illumina-based unbiased metagenomic short-read sequencing and reference-guided assembly using the Wuhan-Hu-1[14] sequence. Our team has also developed an amplicon-based sequencing approach that can dramatically increase the scale of testing. We will continue to optimize technical conditions for viral sequencing from a range of sample types. For example, we will attempt to sequence viral genomes from rapid antigen tests since these tests are commonly used among SARS-CoV-2 positive individuals. To ensure deep coverage of viral genomes and to reduce the cost of sequencing during the current outbreak, the central site will continue to develop and implement methods to concentrate and enrich viral content in all sample types.

Data processing and removing human reads: We will attempt to assemble sequencing reads using our viral assembly pipeline to obtain full viral genomes or partial fragments. The central site's viral analysis and assembly pipeline can assemble and annotate viral genomes in under a day. As part of the process of sequencing any viral and microbial genomes, some human DNA may also inadvertently be sequenced. To improve the quality of the viral analysis and protect participants' genetic information, informatics tools may be used to interrogate whether raw sequencing data derived from ribosomal RNA (rRNA), general human RNA (mRNA) or viral RNA. All human results will be securely destroyed before the analysis phase; only viral and microbial genomes will be analyzed.

We will describe the number of reads, quality, and viral coverage for each sample. Then, we will investigate the similarity of the sequences (viral contigs, unknown reads) using similarity searches and phylogenetic visualization. In addition to these genomic and phylogenetic analyses, we will employ mathematical models of disease transmission, such as the widely used SEIR (Susceptible-Exposed-Infected-Recovered) compartmental model[15]. These models are useful tools to determine the epidemiological conditions that could lead to the occurrence of an outbreak[16].

Following deep sequencing on libraries from samples we will conduct sequencing analysis to investigate viral genomes as well as the metagenomic data. We will attempt to assemble sequencing reads using our viral assembly pipeline to obtain full viral genomes or partial fragments. The central site's viral analysis and assembly pipeline can assemble and annotate viral genomes in under a day. This toolset also assists in the generation of submission files for NCBI SRA and Genbank.

Genome-wide phylogenetic analysis will be performed using multiple tools, including maximum likelihood reconstruction using RAxML, iQ-TREE, or FastTree, and Bayesian reconstruction using BEAST. Evolutionary dynamics will be studied using BEAST to estimate rates of evolutionary change, to estimate the time to the most recent common ancestor, and to perform phylogeographical analyses. Employing tools that we have developed in other contexts, we will use genetic distances and within-host variants to infer transmission networks and to estimate the number and likelihood of unobserved infected individuals and transmission events.

Part C analysis:
We will examine the public health and participant survey data from participants who consent to Part C of this research study to quantify the effectiveness of prevention and containment interventions[17–20].

Researchers can further analyze survey information to look for correlations between COVID-19 infection, the genetic information from the virus that infected a positive participant, symptoms, contacts, and/or demographics using data from Part A-C. Linking viral genome sequence data with survey responses, diagnostic results, and vaccination status will allow researchers to gain an understanding of disease transmission patterns and risk factors. Results will be used to inform public health decisions within these organizations and potentially beyond.

Part D metadata and geolocation community clustering analyses:
Contact networks, transmission networks, and infectious disease models will be created to simulate how disease spreads throughout an entire community setting. Central site researchers and researchers from Degree Analytics will study how the connectivity of communities is correlated to the spread of disease. This includes calculating epidemiologic measures such as but not limited to risk ratios, confidence

intervals for risk, likelihood of infection across campus, and attack rate. Further work will be done using metadata to confirm the most likely transmission route between individuals.

Degree Analytics' role in analysis:
Degree Analytics is a company which uses big data processing analytics tools to help universities understand student behaviors and success. They are currently providing services to University Z by analyzing WiFi connectivity data. For this research, a member of Degree Analytics will be responsible for transforming Degree Analytics' identifiable, standardized connectivity data outputs into usable, de-identified formats for more detailed analysis. A 1-way hash or similar randomized coding techniques will be performed to ensure that the risk of re-identification within the dataset is very low. Subsequently, this researcher will engage in research data analysis, studying how student mobility is correlated to SARS-CoV-2 spread in community settings. Please note that while this researcher has access to identifiers through his role at Degree Analytics in providing data to University Z, all data analysis will be conducted using de-identified data.

Fathom's role in analysis:
Fathom is a company which may already partner with participating organizations and provide services for their public health response. Individuals at Fathom will now join this research study. Fathom's goal is to help human subjects research projects evolve and grow through enabling simpler data storage and analysis. Their research involvement may include the following:

1. Technology development
    a. Fathom may serve in an advisory capacity to develop tools and technologies that advance researchers' work within the protocol.
2. Organizing study data
    a. Aiding with deidentification/coding
        i. Fathom already partners with some organizations as a service provider and may have access to identifiers for some participants within this research study. As such, they may also aid with coding study data or perform other data management functions.
        ii. Fathom may write coding scripts to aid researchers with deidentification/coding of data.
    b. Tool-building: Fathom will be building tools to aid with the collection of study data. In this way, Fathom will serve as a liaison between enrollment sites and research sites but won't be collecting study data themselves.
    c. Data maintenance: Fathom may store study data on behalf of central site and organizational researchers.
3. Data analysis and visualization
    a. Fathom may help generate figures to give insight to data trends or improve current drafted research figures.
    b. They may help quantify survey responses, visualize study data, build phylogenetic trees, and more.
4. Contributing to publications

Participating organization's role in analysis:
A participating organization may also help coordinate de-identification of data and data management functions. Researchers at participating organizations may also perform epidemiologic analyses in conjunction with central site, Fathom, or Degree Analytics researchers on coded data. Access to code keys will be controlled by designated researchers at the participating organization.

Data Management

All biospecimen samples will be coded, labeled only with a coded ID number. Analyses at Degree Analytics, central site, and participating organizations will be conducted solely with coded or de-identified data. Researchers at Fathom may use identifiable data for analysis, coding, and data coordination as necessary.

Part A and B: Access to detection and sequencing results:
Detection and sequencing data from Part A and Part B will be stored in coded form at the central site. Coded data will subsequently be shared with Fathom Information Design and participating organizations.

When working with congregate settings, we will return coded data to the study teams working at the congregate setting. We will always include a disclaimer that the data is research grade, and not intended to inform patient care. In some cases, data produced from this study can have public health utility, and the collaborating institution/congregate setting may use it for this purpose. As examples, (1) the virus genome sequence may help uncover key information about viral introductions or transmissions within a community; (2) sequence data may uncover a cryptic infection, not detected by a first diagnostic test, that should be followed up with a clinical diagnostic. Study teams at collaborating institutions may relink the data generated from the study to individuals in their organization to be used for public health purposes.

Part C data management:
Central site researchers may circulate surveys to subject participants using secure databases such as Qualtrics or RedCap. Identifiable study responses may be downloaded onto a secure central site server (Google Drive) by approved study managers with restricted access for more permanent storage.

When a participant consents to Part C and is part of a participating organization, the organization will share their health information data (such as survey response data or other information used for the purpose of disease outbreak response), with researchers. They may do so through a central site-managed google drive or another secure database. Access to this data is password protected and will be limited on an as-needed basis. For example, only researchers analyzing survey responses and health information will have access to view Part C data. Secure methods will be used to transport data between the questionnaire database and the organization, such as Secure Amazon Web Services S3 buckets.

Study leads at participating organizations or central site will conduct surveys containing direct patient identifiers. They, or Fathom, will remove direct patient identifiers and load survey response data and any other relevant health data (such as date of positive COVID-19 test and vaccination status) from consenting participants onto a secure central site-based Google Drive for the necessary researchers to view. All data on the Google Drive will be labeled with coded identifiers, not direct identifiers. Researchers may download this coded data to analyze on their personal laptops.

Part D data management:
For Part D, grouping and geolocation data will be obtained from a participating community setting/organization or from Degree Analytics with permission from the participating organization. Organizations and communities will send grouping data to the central site for analysis via a secure server with restricted access. Study teams at collaborating institutions may relink data to identifiers for the purpose of reaching out to individuals with information about consenting into the study.

Wastewater surveillance data: Wastewater samples will be sent to the central site for testing and analysis, along with coded locations of collection. Sequences will be shared back to organizations and compared to sequences from nasal/saliva samples. Code keys for the wastewater collection locations will be stored by a participating organization, Fathom, or central site project manager.

WiFi connectivity data: Degree Analytics will manage WiFi connectivity data in their computers. The Degree Analytics' Raw Sessions WiFi connectivity dataset will be de-identified using a hash or similar de-identification process. After sending the code key via a secure server to the participating organization (University Z), Degree Analytics researchers will subsequently delete the code key file linking the Raw Sessions dataset to the de-identified table, to prevent the possibility of re-identification at Degree Analytics. This means that all WiFi connectivity data analyses at Degree Analytics will be conducted on de-identified data. The de-identified dataset may be forwarded to the central site for analysis from Degree Analytics or University Z via a secure database.

Data Storage and Confidentiality

Data at the central site: Access to all information is protected, requiring at minimum a username and password for access. All central site owned and operated systems (those known as “On-Prem” systems) are secured within a co-located data center. These systems are behind the central site firewall. They comply with all relevant security policies for authenticated, secure and minimum access. All systems are patched, monitored, and scanned routinely for vulnerabilities and intrusions. Data is encrypted, where applicable, in compliance with state and federal government standards, regulations, and in accordance with security and privacy policies. All configuration changes that could affect accessibility or security are approved by management. Anti-virus software is used on all computers, along with other monitoring. Human subject data is always subjected to appropriate security controls. Periodic audits ensure that appropriate controls are in place for access to protected data.

The central site will be responsible for storing consent forms for individuals across all enrollment sites and for maintaining the Google Drive. The Google Drive will be managed by the central site, will be password-protected using 2-factor authentication, and will be encrypted to ensure data is shared only between the intended collaborators and organizations. Folder access within the drive will be restricted to collaborators on an as needed basis, and controlled by the PI and project managers at the central site. Several levels of access will be set in the Google Drive based on study role: data analysis, study management, and study code keys (if applicable). As the lead study site, project managers at the central site may have access to direct identifiers as part of their role in orchestrating study recruitment and research data across all sites. Analysis will only be conducted on coded or de-identified data.

Information may also be uploaded to the central site’s Terra system. Terra is a software platform for large-scale scientific analysis of genomic data. The central site is contractually obligated to operate Terra at the FISMA (Federal Information Systems Management Act) "moderate" level. FISMA is a practice of documentation, audit, and organizational risk acceptance. Covered topics include network penetration testing and assessment by a federally authorized outside firm, maintaining system logs separate from the primary system for forensic analysis, regular review of logs and changes by an in-house auditor, security training and background screening for staff with elevated access to the system, and documented procedures to respond to security incidents. In addition to several government FISMA authority to operate (ATO), Terra is FedRAMP (Federal Risk and Authorization Management Program) In Process with expected ATO in early 2021.

Coded data may also be uploaded into a private, central site-owned GitHub repository. Access to this GitHub site is restricted to individuals with a central site GitHub account who are directly added as researchers on the project (in addition to IT admin at the central site). Only the deidentified code, de-identified sequencing data and associated metadata will be released upon publication into a new public GitHub repository.

Data at enrollment sites (University Z): Enrollment sites will securely store collected consent forms for participants recruited to their site and will be responsible for communicating information about recruited participants to project coordinators at the central site. Enrollment sites also may store code keys for data referring to participants recruited at their site, if they are stored in a secure database.

Data at Fathom: Fathom may maintain code keys and identifiable datasets for researchers in this protocol. By using identifiable datasets, Fathom can cross-check coded datasets used by different groups of researchers and ensure that all groups are referencing the correct individuals, while helping us limit the movement of identifiable information. Fathom may additionally aid in protecting data confidentiality by coding study data for individual research sites. Lastly, it is important that Fathom work with identifiable datasets can help with debugging the tools that they are developing for researchers.

1. Confidentiality measures at Fathom: Fathom uses a HIPAA-compliant Amazon Web Service (AWS) architecture using a shared responsibility model. The architecture employs a combination of access control mechanisms, cryptographic mechanisms, and redundancy to maintain confidentiality of the data at-rest and in-transit. All data is encrypted at rest. Data backups are used and stored under restrictive permissions. All access and activity are logged and alerted via AWS Cloudwatch to detect potential security violations.

2. Data transfer with Fathom: Data will be stored and transferred in a secure manner, including but not limited to Amazon Web Services S3 buckets, a central site-hosted Google Drive, University Z Qualtrics, Signal, or relational database service (RDS) databases. Fathom will use restrictive identity access management policies to ensure that data is accessed on an as-needed basis by approved study members or research collaborators.
3. Coded identifier key: Fathom may provide services to code research datasets. The code key will be stored on-site at Fathom, and/or may be released to research collaborators at the participating organization and study managers. Code keys will be stored separately from coded and de-identified data. All study computers and devices will be password protected, with data stored only on secure servers.

<u>Data at Degree Analytics:</u> WiFi data will be de-identified using a 1-way hash. A code linking the hashed IDs and individuals will be sent to the data owner (University Z) via a secure mechanism such as an S3 bucket. This code will be stored on a secure database such as a password-protected organizational Microsoft Teams folder at University Z. This code key will be deleted at Degree Analytics, and analyses will only be conducted on de-identified data.

<u>Data shared between organizations:</u>

1. Coded data will be transferred across organizations using a secure database such as the central site-hosted Google Drive. Information attached to a sample may include data such as: participant's coded ID number, survey response data, viral sequencing data, or diagnostic and experimental pathogen detection test results. The Google Drive will be managed by a designated research team member at the central site. Risk will be minimized in Google Drive and any secure database by (1) creating a central place for research documents, limiting the movement of spreadsheets across organizations; (2) tracking edits and log access to monitor account activity; (3) requiring passwords for access to secure documents, and (4) the ability to set several tiers of access.
2. Direct identifiers may be shared outside of an enrollment site for the purposes of (1) sharing enrollment and contact information with study coordinators at the central site; or (2) sharing identifiable datasets with Fathom for secure storage and coding. Datasets will be shared using secure databases with password-protected, restricted access such as S3 buckets.

<u>Survey data</u>:

1. Congregating settings/organizations may distribute and store survey data in different ways. To permit access to survey data, organizations may give explicit permission to specific researchers to obtain access to coded or deidentified responses in a secure database. Direct identifiers from organizational surveillance surveys and health applications will be removed before the analysis stage, either by coding at an enrollment site or by Fathom researchers from the organizational database where it is stored. Coded datasets may be distributed between research sites. For example, at University Z, the data will be collected on an app, developed by the company Fathom, which is protected by SAML single sign-ons in a HIPAA compliant manner. That means that the data will be encrypted at rest, and all edits/database access are logged and password protected. Access to data will be limited; researchers with explicit permission from a local participating site will be able to see responses by confirming their identity with a unique password. Coded data will be transferred from the cloud to a local site researcher's personal laptop in a secure manner (for example, Amazon S3 buckets) and can be sent to the central site via a secure database like a central site-operated Google Drive. On central site computers, all data will be stored in a local, encrypted manner as per the standards of the central site. Central site researchers will have access to survey responses linked to coded identifiers as well as other health information related to COVID-19 on the organizational app (such as COVID-19 test results and COVID-19 vaccine information); Central site researchers will not be able to see direct identifiers.
2. Central site-developed surveys: Designated researchers at Central Site and University Z will circulate surveys to research participants using a secure database such as RedCap Cloud or Qualtrics. These researchers will be responsible for monitoring survey responses and

ensuring that central site-developed survey response data is coded before passing it to researchers for analysis. Survey data may be sent securely to Fathom for coding.

3. COVID-19 positive surveillance survey data: Organizations may distribute and store survey data in different ways. Organizations may give explicit permission to specific researchers to obtain access to a secure database with coded/de-identified data. An organization may also permit Fathom to code, securely distribute, and analyze survey responses. Survey responses will be coded, though Fathom may see direct identifiers related to this survey data. Coded survey data will be stored on a secure database at the central site, such as a central site-hosted Google Drive. Survey responses will be password-protected and will only be accessible to researchers in this study. Analysis of coded data may be performed on researchers' personal laptops.

Consent forms:

An electronic consent form will be made available in several ways:

1. The electronic consent form will be developed in a secure, web-based, data collection platform, such as REDCap or Qualtrics, with a user management system allowing project owners to grant and control varying levels of access to data collection instruments and data (e.g., read only, coded-only data views) for other users. REDCap is a HIPAA-compliant data management system which uses encryption, data tracking/logging, and secure authentication to protect participant confidentiality. Qualtrics is similarly approved to collect and store sensitive, identifiable data.
2. An enrollment site will send a copy of the consent form and provide a secure database for potential participants to upload their signed form. For example, University Z may provide an organizational Microsoft Teams folder for a participant to upload their documents.
3. Written consents will be stored in a locked filing cabinet at the enrollment site and may be scanned and uploaded to a secure database hosted by the enrollment site. Access to consent forms will be restricted to authorized study personnel, such as researchers involved in study consent management at the central site or lead organizational researchers. Participants will be provided copies of their written or electronic consent form via email.
4. Central site project managers will maintain a restricted Google Drive folder to help organize and track participants across all study sites.

Keys linking coded data to direct participant identifiers: The key linking participants' identifying information to a coded identifier will be maintained by lead organizational researchers at each enrollment site or by Fathom. Code keys will not be shared between researchers at different enrollment sites, but project managers at the central site may have access to code keys at different enrollment sites for the purpose of contacting participants or distributing surveys. Central site researchers will not have access to the key for the purpose of data analysis. At University Z, the key will be stored on a network computer in a secure, password protected internal network R-Drive (not on the computer hard drive). Only authorized study personnel will be provided with an account and password allowing them to access this key. Authorized representatives of the following groups may need to review this research as part of their responsibilities to protect research subjects: representatives of the IRB, regulatory agencies, and federal oversight agencies. The study team will permit access to such records by communicating with appropriate researchers at the participating organization and allowing them to release the information in a secure, password-protected manner to these agencies for compliance regulation purposes.

Genomic data sharing through a repository: All samples have been coded by study researchers at the participating organizations. The data will be uploaded to the repository using these codes. No PHI will be submitted to the repositories, except for the date of sample collection and a participant's county of collection. It is important to include the sample collection date because this represents the date at which the virus stopped replicating, which is important when considering the spread of viral strains or mutations within the context of a pandemic. Similarly, the county of collection will aid general surveillance efforts. The fields uploaded to these repositories will be publicly available and not restricted in any way.

Information uploaded to the repository will include:

- SARS-CoV-2 genomic sequence and other microbial/viral genome sequences
- their prevalence within the research study (%)
- where a sample was collected (state and county level)

- date of sample collection
- host (human)
- isolation source (nasal fluid, saliva, rapid antigen test, etc.)

Communication with study participants: Enrollment sites will maintain contact with study participants through study leads and staff, who are appointed by the organization to have access to the key to the code containing participant IDs.

An enrollment site contact has been provided for study participants for them to ask any questions. Additionally, study coordinators at the central site will interact with study participants through a central site-managed study Gmail account. This email account has been established for participants or potential participants to report any issues and ask questions. If a participant discloses their coded identifier, study coordinators may inadvertently be able to identify participants' samples. Study coordinators will not share information they learn through the study email account with other central site researchers, nor will they make any attempt to look up an individual participant's data unless helping the participant withdraw from the study.

Transition from research participation: Since patient care is not provided through this protocol, when subjects terminate their participation in the research, researchers will simply no longer collect research data from this subject. If part of an organization, the research participant will retain the ability to participate in all their organization's COVID-19 response efforts, including diagnostic testing. Access to these services does not change based on study participation status.

Risk/Benefit Assessment

Risk category: This research presents as minimal risk.

Potential risk:

1. Though the study is minimally invasive with few health risks, there is risk of a breach of participant confidentiality. We will obtain a variety of data pertaining to participants' health, habits, contacts, and we will conduct viral diagnostics and genomics.
2. Participation of study team members: Study team members and their close contacts will not be excluded from participating, as their data will be essential to developing a comprehensive picture of transmission. Study team members' personal data may thereby be shared with other members of the study team and there is a risk that study team members involved in the study may misuse study data for personal reasons.
3. As part of the process of sequencing any viral and microbial genomes, some human DNA may also inadvertently be sequenced. This increases the risk that researchers may learn information about an individual through their genome.
4. Collection of nasal swabs (such as nasopharyngeal swabs or foam-tipped oral-nasal swabs), rapid antigen tests, or saliva samples, can be uncomfortable or anxiety-producing for some individuals, though some participants will be required to be tested per their organizational policy, regardless of participation in our study.
5. Undue influence: Given that the study is offered in collaboration with an organization which may be a participant's employer, school, or fulfill another supervisory role, participants may naturally feel pressure to enroll in the research study.

Protection against risks:

1. To minimize the risk that one or more individuals gain inappropriate access to private or identifiable information, we will host study data in secure systems, restrict access to data, and implement a coding system for participant data. Information will be strictly maintained to ensure confidentiality. Data will be coded during analysis and protected with robust security measures. Designated managers, researchers at Fathom, or principal investigators at each site will be allowed to view study code keys for purposes of managing the data; outside of these individuals, access to identifiable data will be restricted. Study managers and the PI will oversee coordinating data between sites, communicating data restrictions, and enforcing data safety measures. They will also oversee contacting study participants if necessary (such as if returning research results

or distributing survey questions). All study computers and devices will be password protected, with identifiable data stored only in secure servers. Finally, participants will not be required to contribute any information that they are uncomfortable disclosing for any reason. For more information about data storage, see “Data Storage and Confidentiality.” There is an additional confidentiality risk from Part D; although the metadata we collect are not identifying on their own, we acknowledge that together our clustering data may be close to becoming identifiable. For this reason, we will employ the same data management and safety techniques for Part D.

2. For any study team members which participate in the research in Parts A-C and have access to study team data, an attestation form will be required. This form will ensure that the risks associated with the dual role of a participant and study team member are understood, and that the study team participant acknowledges that their study colleagues will potentially have access to identifying information about them. To protect other study participants from potential misuse of data:
    a. A participating study team member must attest that they will abstain from study design decisions from which they could have a conflict of interest based on their involvement as a study subject, and that they will make no attempt to inappropriately access study data (e.g., such as to discover the identities of other research subjects whom they may have infected or who may have infected them).
    b. Enrollment sites will enforce a management plan (for example, changing password-protected key to identifiable data every time a study team member enrolls).
    c. Enrollment sites will ensure interactions between these individuals and the outside collaborators only includes discussion of coded data.

3. To minimize the amount of data that can be extrapolated about an individual from the sequencing process, only viral and microbial genomes, not human DNA, will be analyzed. As per our standard practice, informatics tools may be used to interrogate whether raw sequencing data derived from ribosomal RNA (rRNA), general human RNA (mRNA) or viral RNA. All human results will be securely destroyed before the analysis phase.

4. To minimize anxiety caused by study procedures, individuals will have the option to consent into providing samples. If they do not feel comfortable providing these samples, they are not required to submit them as part of the research study.

5. To protect against inaccurate interpretation of results when returned to collaborating organizations, all return of results will include the disclaimer that results of specific tests are research grade, not FDA approved, and therefore should not be used to inform clinical care.

6. To minimize any undue influence on potential participants to sign up for this study, both written and verbal communication pertaining to the study will emphasize its voluntary nature and assure participants that there will be no adverse consequences should they choose not to participate.

<u>Potential benefits to the subjects:</u> There are no direct benefits to study participants from taking part in this research. Subjects are not paid for participating in this study.

<u>Alternatives to participation:</u> The alternative to participating in this study is to not participate in the study.

If an individual is a member of the community or surrounding community of an organization participating in our research, the individual can opt in or out of any research arm offered in the consent form. They can also seek out COVID-19 testing via local public health services, without participating in this study.

<u>Subject Identification, Recruitment, And Consent/Assent</u>
Recruitment will occur throughout the research study, as long as there is a risk of COVID-19 disease transmission.

Recruitment will occur within a congregating setting as follows:

1. Participating organizations already have areas for routine COVID-19 testing and/or surveillance. On-site researchers will ensure consent forms and recruitment documents are available near routine testing sites to encourage individuals, whether from within the organization or from the surrounding community, to join the study.
2. Similarly, individuals who test positive for COVID-19 may learn about our study from study leads at the organization through phone or email.
3. Recruitment documents will be circulated to individuals within participating organizations via email listservs and posters by researchers or other members of the organization (such documents have been included within this application for University Z).
4. With organizational permission, recruitment documents may also be distributed by researchers throughout the surrounding community via email, email, or posters.
5. If the clustering analysis in Part D reveals an interesting case which could shed light on disease spread and transmission, central site researchers may request that the designated researcher at the participating organization use the code-key to re-identify the individual and then have designated personnel at the participating organization contact the individual with information about this study. If an individual chooses to do so, they may subsequently consent into Parts A-C.

Central site study staff will recruit individuals in the following ways:

1. Recruitment material will be provided over email via a study email or from researchers' central site-institute email accounts to potential research participants. Potential research participants will be identified through the following ways:
    a. Organizations may provide a list of emails of students. For example, they may provide a list of emails of incoming freshman, or an email list of those who are planning on attending an event. Organizations will not disclose an individuals' testing status for SARS-CoV-2 nor any other respiratory disease, but instead will provide a general list of emails for individuals whose infection status could be known or unknown.
    b. Use of existing email listservs, such as a listserv for an affiliation group at an organization. As an example, we would be interested in sending out recruitment emails for our study to university students studying biology via the university's biology departmental listserv.
2. Recruitment material posted on the lab website.
3. Recruitment material provided at physical locations (with QR code to the lab website or to the consent form).
4. Social Media including but not limited to: Twitter, Facebook, and Instagram.

Study staff may use email to communicate with site administrators or research subjects prior to consenting but information contained in the emails will be limited to answers to general questions about the study or confirm testing dates and times. All emails to subjects will be sent from organizational accounts such as an official University Z email or a Gmail account monitored by the central site.

To reduce risk of coercion through recruitment of employees and/or students, both written and verbal communication pertaining to the study will emphasize its voluntary nature and assure participants that there will be no adverse consequences should they choose not to participate.

Process of Consent

Study Arms A, B, and C consent: Research Arms A, B, and C will involve collection of consent forms from individual study participants.

Consent could be collected through an inked or electronic signature on a paper or online consent form. Online consent forms are intended to limit the potential of coercion or undue influence, as they limit involvement of study staff within the consent process. Participants will be made aware that participation is completely voluntary.

The informed consent process will be performed as follows:

- The study information and a copy of an electronic consent form will be provided to potential participants through self-initiated access of consent forms on personal portable electronic devices using posted QR codes or web-links on study posters, brochures, a study website, social media, or emails.
  - Self-initiated accessing of consent forms may occur on-site at an organization, at home, and/or after being approached by a designated researcher in-person, by phone, over the internet, or email. Participants may also access the consent via paper form, tablet, or other electronic device on-site at a participating organization.
- The research subject will be given the opportunity to read the informed consent form. Participants can ask questions and have all questions answered by the study team member in person, by email, phone, or video chat. The individual may spend as long as they wish reviewing the form.
- The informed consent document must be signed and dated by the research subject. As part of the screening process, the participant will answer questions to certify that they are above the age of majority.
- Electronic Consent Forms:
  - Electronic consent will take place using a secure online platform such as REDCap or Qualtrics. Participants will sign electronically on the online platform.
  - The research subject will be able to obtain a copy of the consent (e.g., through email, on the secure online consenting portal, or by contacting the study team).
  - Electronic consent forms will be stored online in a secure database. Access to these consent forms will be controlled and password protected. Only researchers whose jobs involve management of study enrollment and consent will have access to these consent forms.
- Written Consent Forms:
  - Written informed consent documents may be made available to participants. These documents will be signed, and a copy can be provided to the participant. Signed informed consent documents may be scanned and uploaded to the secure database. Any physical documents will be stored in a locked filing cabinet only accessible by appropriate study staff.
- A participant can withdraw or adjust their consent as desired by contacting the study team via the provided contact information.

Auditors/witnesses will not be used within this study.

<u>Study Arm D consent:</u> We are requesting a waiver of consent for data collected under research arm D. The study meets the criteria for a waiver of consent as follows:

The research involves no more than minimal risk to the subjects.
- All data for Study Arm D will be collected with permission from the institution where the data originates.
- Grouping metadata: Research is a secondary use study of samples and data previously collected for surveillance purposes.
- WiFi connectivity data: The WiFi connectivity dataset from Degree Analytics is minimally different from information gained through conventional contact tracing, a process also underway as part of public health efforts to contain the spread of infectious disease. The identity of individuals will be coded so they are not identifiable to the research team at the central site or Degree Analytics researchers during analysis, and the location of Wi-Fi access points will be coded during analysis, so the precise location is not known at a resolution below the level of coded individual buildings.
- Excess clinical samples: Secondary-use samples will be coded, and the code key will be restricted to Fathom Information Design and/or the participating institution as part of their public health response efforts.
- Wastewater samples: Wastewater samples contain waste from many individuals and cannot be traced back to a specific person.

The research could not practicably be carried out without the requested waiver or alteration.

- Secondary-use data and other pathogen detection samples may have been collected from any individuals within a community who may have graduated, moved, or otherwise be uncontactable, and unavailable to provide consent. For wastewater samples, it is unclear who might have contributed to the waste at a particular site.
- To provide for a control group and basis for comparison when considering aggregate epidemiological statistics, it is critical to obtain data from as many individuals as possible. Incorporating all available data allows for accurate estimation of risk using appropriate denominators and allows our team to reduce selection bias within our study.
- It is important to understand which affiliations/groups increase the risk of disease, and which do not impact an individual's risk of infection. As such, we need a dataset describing how individuals are clustered within an organization across many groups.

If the research involves using identifiable private information or identifiable biospecimens, the research could not practically be carried out without using such information or biospecimens in an identifiable format.

- This research is unique because analyses are directly informing public health responses in community settings. Although our research will limit the amount of identifiable private information, links need to be retained to individuals to respond to active health crises. It is also important to maintain code keys to the dataset to link multiple datasets together and create a more detailed picture of infectious disease outbreak within a community.
- WiFi connectivity data: The WiFi connectivity dataset incorporated into this research will be coded through a hashing mechanism. Code keys may be retained at the participating organization/community setting or at Fathom. This WiFi connectivity dataset will then be linked to test positivity status and date of infection, to understand when an individual was positive and moving across campus. This can help indicate if a variant is more infectious if an individual has a lower number of contacts than average but infected a higher proportion of people.
- Grouping metadata: While each field on its own is not individually identifiable, we are aware that combining such information increases the risk of identification. Our research team pledges to never attempt to re-identify individuals using grouping data. Publications will focus on risk of transmission, transmission of disease, and infection between groups. Each one of these components is important to understanding data gaps (e.g., reporting delays, missed transmission links), disparities in disease burden, and patterns of disease on campus. Extensive transmission and contact data are especially critical to understanding which epidemiological links between individuals contribute to a higher risk of transmission.
- Excess clinical samples: Samples will be coded outside of a participating organization and Fathom. It is important to maintain a code key outside of research to roll out appropriate public health responses. A code key is also necessary to match sequencing data with grouping metadata. Together, sequencing data and grouping metadata will be used to distinguish which group affiliations contribute to the transmission of disease.
- By comparing sequences within wastewater samples to clinical samples, if two sequences match, researchers may be able to assume where a participant was located (e.g., what dorm they live in). Locations will be coded. Comparing wastewater to clinical samples is essential to understanding the number of undetected infections in a community, as well as understanding the usefulness of wastewater data.

The waiver or alteration will not adversely affect the rights and welfare of the subjects.

- WiFi connectivity dataset: For the WiFi connectivity dataset, the university and Degree Analytics will continue to collect and use Wi-Fi data independent of this study for public health purposes. Students were informed upon signing into the organizational WiFi that their locations were being tracked for public health purposes. Students retain the ability to opt-out of tracking at any time.
- Excess clinical samples: Secondary-use samples have already been collected or will continue to be collected for other purposes, either related to disease surveillance or research.
- Grouping metadata: Coded individual-level data is aggregated for several organizational groups, then analyzed across groups. Each field on its own is not individually identifiable, and results returned to a participating organization will summarize higher/lower-risk groups rather than identifying problematic individuals.

- Wastewater samples: These samples are collections of waste from many individuals. While some sequences may be similar to clinical sequences, we cannot be sure whether a specific sequence comes from a study participant, or from an undetected case within the community. No identifiable private information is associated with wastewater samples.

Whenever appropriate, the subjects or legally authorized representatives will be provided with additional pertinent information after participation.

Subject capacity: All subjects participating in the research will have the capacity to participate in research; individuals with diminished capacity will not be involved within this research.

Subject/representative comprehension: To ensure comprehension, the presentation of information in the consent form has been adapted to middle-school level English and scientific terms are explained. This ensures that potential participants from all academic backgrounds/occupations will be able to understand the research aims. The risks of this research are low but are still explained on the form. Researchers will be present at the consent site to answer any additional questions. An email has also been provided for potential participants to ask questions.

Debriefing procedures: No information will be purposely withheld from research participants, and thus debriefing will not be conducted.

Consent forms: Consent forms will be submitted to this IRB for approval, and will disclose all risks, benefits, and alternatives of research participation.

Documentation of consent: See above in “Process of Consent.”

Costs to the subject: There will be no costs to participants for participating in the study.

Payment for participation: There will be no payment for participation.

Supplemental References

1. Verity R, Okell LC, Dorigatti I, Winskill P, Whittaker C, Imai N, et al. Estimates of the severity of coronavirus disease 2019: a model-based analysis. Lancet Infect Dis. 2020 Jun;20(6):669–77.

2. WHO Coronavirus (COVID-19) Dashboard [Internet]. [cited 2022 Sep 3]. Available from: https://covid19.who.int/

3. Payne S. Chapter 17 - Family Coronaviridae. In: Payne S, editor. Viruses. Academic Press; 2017. p. 149–58.

4. Coronaviruses [Internet]. National Institute of Allergy and Infectious Diseases; [cited 2022 Sep 3]. Available from: https://www.niaid.nih.gov/diseases-conditions/coronaviruses

5. Long QX, Tang XJ, Shi QL, Li Q, Deng HJ, Yuan J, et al. Clinical and immunological assessment of asymptomatic SARS-CoV-2 infections. Nat Med. 2020 Aug;26(8):1200–4.

6. Wu C, Chen X, Cai Y, Xia J 'an, Zhou X, Xu S, et al. Risk Factors Associated With Acute Respiratory Distress Syndrome and Death in Patients With Coronavirus Disease 2019 Pneumonia in Wuhan, China. JAMA Intern Med. 2020 Jul 1;180(7):934–43.

7. Rothe C, Schunk M, Sothmann P, Bretzel G, Froeschl G, Wallrauch C, et al. Transmission of 2019-nCoV Infection from an Asymptomatic Contact in Germany. N Engl J Med. 2020 Mar 5;382(10):970–1.

8. Premraj A, Aleyas AG, Nautiyal B, Rasool TJ. Nucleic Acid and Immunological Diagnostics for SARS-CoV-2: Processes, Platforms and Pitfalls. Diagnostics (Basel) [Internet]. 2020 Oct 23;10(11). Available from: http://dx.doi.org/10.3390/diagnostics10110866

9. Wang D, Hu B, Hu C, Zhu F, Liu X, Zhang J, et al. Clinical Characteristics of 138 Hospitalized Patients With 2019 Novel Coronavirus–Infected Pneumonia in Wuhan, China. JAMA. 2020 Mar 17;323(11):1061–9.

10. CDC. COVID-19 Serology Surveillance Strategy [Internet]. Centers for Disease Control and Prevention. 2021 [cited 2022 Sep 3]. Available from: https://www.cdc.gov/coronavirus/2019-ncov/covid-data/serology-surveillance/index.html

11. Doerr M, Grayson S, Moore S, Suver C, Wilbanks J, Wagner J. Implementing a universal informed consent process for the All of Us Research Program. Pac Symp Biocomput. 2019;24:427–38.

12. Ackerman CM, Myhrvold C, Thakku SG, Freije CA, Metsky HC, Yang DK, et al. Massively multiplexed nucleic acid detection with Cas13. Nature. 2020 Jun;582(7811):277–82.

13. Lemieux JE, Siddle KJ, Shaw BM, Loreth C, Schaffner SF, Gladden-Young A, et al. Phylogenetic analysis of SARS-CoV-2 in Boston highlights the impact of superspreading events. Science [Internet]. 2021 Feb 5;371(6529). Available from: http://dx.doi.org/10.1126/science.abe3261

14. Wu F, Zhao S, Yu B, Chen YM, Wang W, Song ZG, et al. A new coronavirus associated with human respiratory disease in China. Nature. 2020 Mar;579(7798):265–9.

15. Anderson RM, May RM. Infectious diseases of humans: Oxford University Press. USA; 1992.

16. Brauer, Driessche, Wu. Lecture notes in Mathematics: Preface. Mathematical Epidemiology [Internet]. Available from: https://asu.pure.elsevier.com/en/publications/lecture-notes-in-mathematics-preface

17. Nishiura H, Patanarapelert K, Sriprom M, Sarakorn W, Sriyab S, Ming Tang I. Modelling potential

responses to severe acute respiratory syndrome in Japan: the role of initial attack size, precaution, and quarantine. J Epidemiol Community Health. 2004 Mar;58(3):186–91.

18. Agusto FB, Del Valle SY, Blayneh KW, Ngonghala CN, Goncalves MJ, Li N, et al. The impact of bed-net use on malaria prevalence. J Theor Biol. 2013 Mar 7;320:58–65.

19. Daughton AR, Generous N, Priedhorsky R, Deshpande A. An approach to and web-based tool for infectious disease outbreak intervention analysis. Sci Rep. 2017 Apr 18;7:46076.

20. Lewnard JA, Grad YH. Vaccine waning and mumps re-emergence in the United States. Sci Transl Med [Internet]. 2018 Mar 21;10(433). Available from: http://dx.doi.org/10.1126/scitranslmed.aao5945
