# Supplementary material for: Multimodal surveillance of SARS-CoV-2 at a university enables development of a robust outbreak response framework
Source: Med. 2022 Dec 9;3(12):883–900.e13. doi: 10.1016/j.medj.2022.09.003 (PMC9482833; doi:10.1016/j.medj.2022.09.003)
Supplement: Document S2. Article plus supplemental information [file mmc4.pdf]

Med

CellPress
OPEN ACCESS

Clinical and Translational Resource and Technology Insights

Multimodal surveillance of SARS-CoV-2 at a university enables development of a robust outbreak response framework

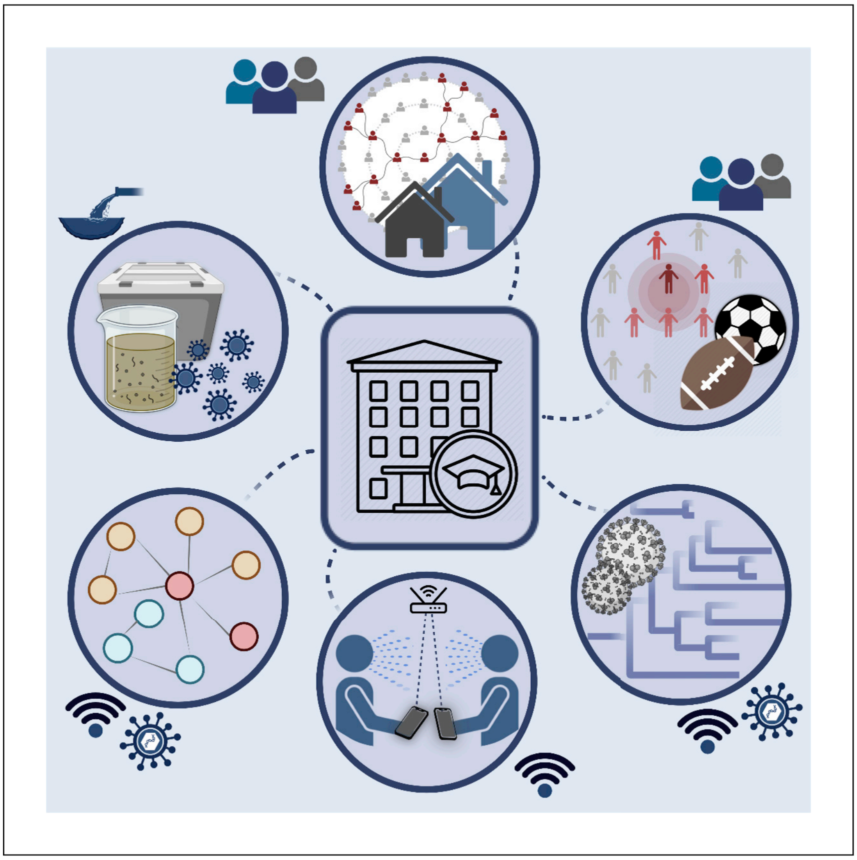

Petros et al. studied SARS-CoV-2 transmission at a university by integrating epidemiological metadata, Wi-Fi-based location data, and viral sequences from wastewater and clinical samples. They identified a novel viral lineage and used digital contact tracing, genomics, and functional assays to delineate the social and biological factors contributing to its expansion.

Foundational Research

Brittany A. Petros, Jillian S. Paull, Christopher H. Tomkins-Tinch, ..., Amy Bronson, Stephen F. Schaffner, Pardis C. Sabeti

jpaull@broadinstitute.org (J.S.P.)
tomkinsc@broadinstitute.org (C.H.T.-T.)
bloftnes@broadinstitute.org (B.C.L.)

Highlights

Surveillance of SARS-CoV-2 transmission at a public university

Wi-Fi co-location data identified social behaviors associated with infection

Overdispersion in virus transmission consists of both social and biological components

Concurrent clinical sample sequencing led to wastewater variant-calling criteria

Petros et al., Med 3, 883–900
December 9, 2022 © 2022 The Authors.
Published by Elsevier Inc.
https://doi.org/10.1016/j.medj.2022.09.003

Check for updates

Med

CellPress OPEN ACCESS

Clinical and Translational Resource and Technology Insights

Multimodal surveillance of SARS-CoV-2 at a university enables development of a robust outbreak response framework

Brittany A. Petros,[1,2,3,4,27] Jillian S. Paull,[1,4,27,*] Christopher H. Tomkins-Tinch,[1,5,27,28,*] Bryn C. Loftness,[1,6,7,8,27,*] Katherine C. DeRuff,[1] Parvathy Nair,[9] Gabrielle L. Gionet,[1] Aaron Benz,[10] Taylor Brock-Fisher,[1] Michael Hughes,[11] Leonid Yurkovetskiy,[12] Shandukani Mulaudzi,[13] Emma Leenerman,[11] Thomas Nyalile,[12] Gage K. Moreno,[1] Ivan Specht,[1] Kian Sani,[1] Gordon Adams,[1] Simone V. Babet,[14] Emily Baron,[15] Jesse T. Blank,[11] Chloe Boehm,[1,16] Yolanda Botti-Lodovico,[1] Jeremy Brown,[11] Adam R. Buisker,[11] Timothy Burcham,[17] Lily Chylek,[1] Paul Cronan,[17] Ann Dauphin,[12] Valentine Desreumaux,[14] Megan Doss,[18] Belinda Flynn,[11] Adrianne Gladden-Young,[1] Olivia Glennon,[17] Hunter D. Harmon,[11] Thomas V. Hook,[14] Anton Kary,[19] Clay King,[20] Christine Loreth,[1] Libby Marrs,[17] Kyle J. McQuade,[19] Thorsen T. Milton,[14] Jada M. Mulford,[19] Kyle Oba,[17] Leah Pearlman,[1] Mark Schifferli,[17] Madelyn J. Schmidt,[11] Grace M. Tandus,[14] Andy Tyler,[11] Megan E. Vodzak,[1] Kelly Krohn Bevill,[6] Andres Colubri,[1,21] Bronwyn L. MacInnis,[1] A. Zeynep Ozsoy,[19] Eric Parrie,[15] Kari Sholtes,[6,14] Katherine J. Siddle,[1,5] Ben Fry,[17,26] Jeremy Luban,[1,12,22,23,26] Daniel J. Park,[1,26] John Marshall,[11,26] Amy Bronson,[24,26] Stephen F. Schaffner,[1,26] and Pardis C. Sabeti[1,5,9,23,25,26]

SUMMARY

Background: Universities are vulnerable to infectious disease outbreaks, making them ideal environments to study transmission dynamics and evaluate mitigation and surveillance measures. Here, we analyze multimodal COVID-19-associated data collected during the 2020–2021 academic year at Colorado Mesa University and introduce a SARS-CoV-2 surveillance and response framework.

Methods: We analyzed epidemiological and sociobehavioral data (demographics, contact tracing, and WiFi-based co-location data) alongside pathogen surveillance data (wastewater and diagnostic testing, and viral genomic sequencing of wastewater and clinical specimens) to characterize outbreak dynamics and inform policy. We applied relative risk, multiple linear regression, and social network assortativity to identify attributes or behaviors associated with contracting SARS-CoV-2. To characterize SARS-CoV-2 transmission, we used viral sequencing, phylogenomic tools, and functional assays.

Findings: Athletes, particularly those on high-contact teams, had the highest risk of testing positive. On average, individuals who tested positive had more contacts and longer interaction durations than individuals who never tested positive. The distribution of contacts per individual was overdispersed, although not as overdispersed as the distribution of phylogenomic descendants. Corroboration via technical replicates was essential for identification of wastewater mutations.

Conclusions: Based on our findings, we formulate a framework that combines tools into an integrated disease surveillance program that can be implemented in other congregate settings with limited resources.

CONTEXT AND SIGNIFICANCE

Understanding the factors that influence the transmission of SARS-CoV-2 and other pathogens is critical to mitigate outbreaks. Here, researchers at Colorado Mesa University and at the Broad Institute of MIT and Harvard designed and assessed an infectious disease surveillance program that included diagnostic testing and symptom reporting, wastewater surveillance, and digital contact tracing of students at a university. Viral spread on campus was a result of social behaviors, such as the number of contacts and the types of interactions an infected person had, and biological factors, such as the SARS-CoV-2 variant an infected person harbored. The authors' findings highlight the need to design surveillance

This is an open access article under the CC BY license (http://creativecommons.org/licenses/by/4.0/).

Funding: This work was supported by the National Science Foundation, the Hertz Foundation, the National Institutes of Health, the Centers for Disease Control and Prevention, the Massachusetts Consortium on Pathogen Readiness, the Howard Hughes Medical Institute, the Flu Lab, and the Audacious Project.

programs that capture social behaviors and viral lineage information in congregate communities.

INTRODUCTION

Infectious disease outbreaks are existential threats to congregate communities; universities, in particular, are susceptible because of close-quarters housing,[1,2] dense social networks,[3–5] and widespread involvement in sports teams and other student organizations.[5,6] Students may also be individually vulnerable to infection due to sleep deprivation[7] and poor hygiene.[8] In addition to their own susceptibility, universities have potential to drive transmission in surrounding communities.[9–11]

At the same time, residential universities are ideal environments for the study of pathogen transmission and the impact of interventions due to their semi-insular nature and their role as centers of innovation.[12] In response to SARS-CoV-2 they widely employed high-cadence testing,[13–15] vaccination programs,[16,17] strict isolation of cases in dedicated facilities,[18–21] and social distancing measures.[22–25] In addition, universities are well-positioned to test and implement new surveillance methods that can subsequently be applied at greater scale. For example, they were among the first to implement SARS-CoV-2 wastewater surveillance,[18,26] institution-wide viral sequencing,[21,27] and contact tracing via WiFi network co-location data.[28,29]

Colorado Mesa University (CMU) committed to in-person instruction of approximately 8,000 students for the 2020–2021 academic year, motivated by a desire to avoid amplifying resource disparities via remote learning. This decision necessitated a rigorous SARS-CoV-2 surveillance program, balancing public health goals with efficient use of limited resources. Given these considerations, CMU eschewed mandatory periodic testing of all university members in favor of a surveillance program with randomized testing and robust reflexive testing—i.e., strategic testing of students due to reported symptoms, contact with recently diagnosed individuals, or a positive wastewater signal in their residential dorm.

CMU piloted *Lookout*, a tool integrating multiple data types to identify, alert, and test individuals or groups at increased risk of infection (Figure 1; demo: https://sentinel.network/lookout-demo-campus). Lookout integrated numerous data types, including symptoms (reported through the companion mobile app, *Scout*[30]), clinical diagnostic test results, student attributes (e.g., residence hall and sports team affiliations), self-reported contacts of positive individuals, viral genome sequences from diagnostic specimens, and wastewater viral titers. The interactive dashboard allowed the administration to quickly identify students at risk of infection and to minimize opportunities for transmission. Here, we explore the utility of combining these and additional data types (including WiFi co-location logs and genome sequences from wastewater effluent) to design effective disease surveillance systems.

RESULTS

CMU deployed a comprehensive and effective surveillance program based on a multi-pronged testing approach

Over the 2020–2021 academic year, CMU's surveillance program identified 1,113 COVID-19 cases (1,076 students, 37 faculty or staff) through randomized and reflexive testing. The test positivity rate was 5.1% in Fall 2020 (August 17–November 20)

[1]Broad Institute of MIT and Harvard, Cambridge, MA 02142, USA
[2]Harvard-MIT Program in Health Sciences and Technology, Cambridge, MA 02139, USA
[3]Harvard/MIT MD-PhD Program, Boston, MA 02115, USA
[4]Systems, Synthetic, and Quantitative Biology PhD Program, Department of Systems Biology, Harvard Medical School, Boston, MA 02115, USA
[5]Department of Organismic and Evolutionary Biology, Harvard University, Cambridge, MA 02138, USA
[6]Department of Computer Science and Engineering, Colorado Mesa University, Grand Junction, CO 81501, USA
[7]Complex Systems and Data Science PhD Program, University of Vermont, Burlington, VT 05405, USA
[8]Vermont Complex Systems Center, University of Vermont, Burlington, VT 05405, USA
[9]Howard Hughes Medical Institute, Chevy Chase, MD 20815, USA
[10]Degree Analytics, Inc., Austin, TX 78758, USA
[11]Colorado Mesa University, Grand Junction, CO 81501, USA
[12]Program in Molecular Medicine, University of Massachusetts Chan Medical School, Worcester, MA 01655, USA
[13]Harvard Program in Bioinformatics and Integrative Genomics, Harvard Medical School, Boston, MA 02115, USA
[14]Department of Civil, Environmental, and Architectural Engineering, University of Colorado, Boulder, CO 80309, USA
[15]COVIDCheck Colorado, LLC, Denver, CO 80202, USA
[16]Princeton University Molecular Biology Department, Princeton, NJ 08544, USA
[17]Fathom Information Design, Boston, MA 02114, USA
[18]Warrior Diagnostics, Inc., Loveland, CO 80538, USA
[19]Department of Biological Sciences, Colorado Mesa University, Grand Junction, CO 81501, USA
[20]Department of Mathematics and Statistics, Colorado Mesa University, Grand Junction, CO 81501, USA
[21]University of Massachusetts Medical School, Worcester, MA 01655, USA
[22]Biochemistry and Molecular Pharmacology, University of Massachusetts Medical School, Worcester, MA 01655, USA

Continued

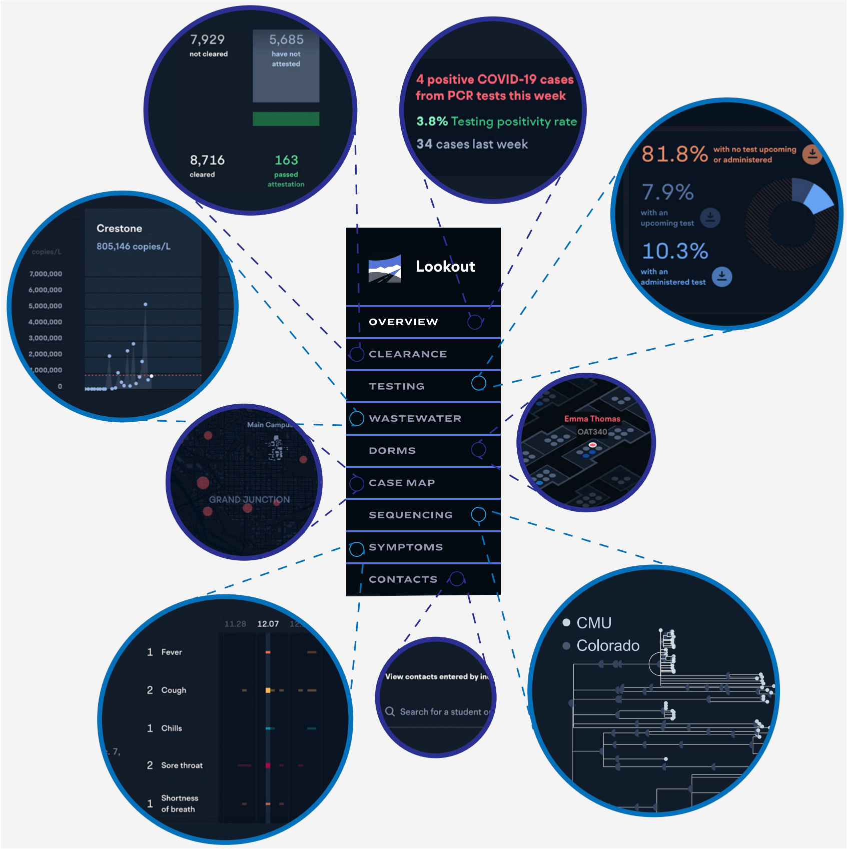

Figure 1. *Lookout* implements real-time monitoring of COVID-19 cases

The *Lookout* tool integrates diagnostic test results, student metadata, viral genome sequences, and wastewater viral titers. A demo with representative synthetic data is available at https://sentinel.network/lookout-demo-campus/. Overview: current data on community case burden, test volume, high-incidence groups, and symptom and exposure attestation. Clearance: counts of the individuals complying with the training and symptom attestation requirements for campus entry. Testing: reports of positive tests in the past 7 days as well as the volume of tests scheduled, taken, and missed for the current week. Testing—baseline: the number of tests administered over time relative to the amount needed to successfully test the entire population before a return to campus. Wastewater: viral loads over time, measured on a per-sewershed basis and aligned with individual test results from the same residence halls. Dorms: spatial position of residence hall cases on a per-floor basis. Individuals may be selected to view their campus associations (i.e., potential close contacts) and current attestation, test, and isolation status. Case map: view of case locations for members of the university community who live off campus, with hot spots for locations of high case density. Sequencing: phylogenetic tree of viral genomes collected from university cases. Individuals may be selected to highlight other individuals who are members of the same cluster. Viral lineages are noted. Symptoms: timelines depict reported symptoms for students or staff, including fever, cough, chills, sore throat, shortness of breath, loss of smell/taste, and runny nose. Contacts: the list of contacts reported by cases, and their associated contact information. Lookup: information on a user-queried individual, including group affiliations, test result history, symptom history, attestation history, and contact history.

and 1.5% in Spring 2021 (January 18–April 30) (Figures 2A–2C); individuals who tested positive were moved to an isolation dorm. CMU's randomized testing strategy sampled students non-uniformly to test those at greater risk of onward transmission, i.e., on-campus students and athletes.

In addition, CMU tested individuals identified by institutional contact tracing as close contacts. Of the identified positive individuals, 720 (65%) reported close

[23]Massachusetts Consortium on Pathogen Readiness, Boston, MA 02115, USA

[24]Physician Assistant Program, Department of Kinesiology, Colorado Mesa University, Grand Junction, CO 81501, USA

[25]Department of Epidemiology, Harvard T.H. Chan School of Public Health, Boston, MA 02115, USA

[26]Senior author

[27]These authors contributed equally

[28]Lead contact

*Correspondence:
jpaull@broadinstitute.org (J.S.P.),
tomkinsc@broadinstitute.org (C.H.T.-T.),
bloftnes@broadinstitute.org (B.C.L.)

https://doi.org/10.1016/j.medj.2022.09.003

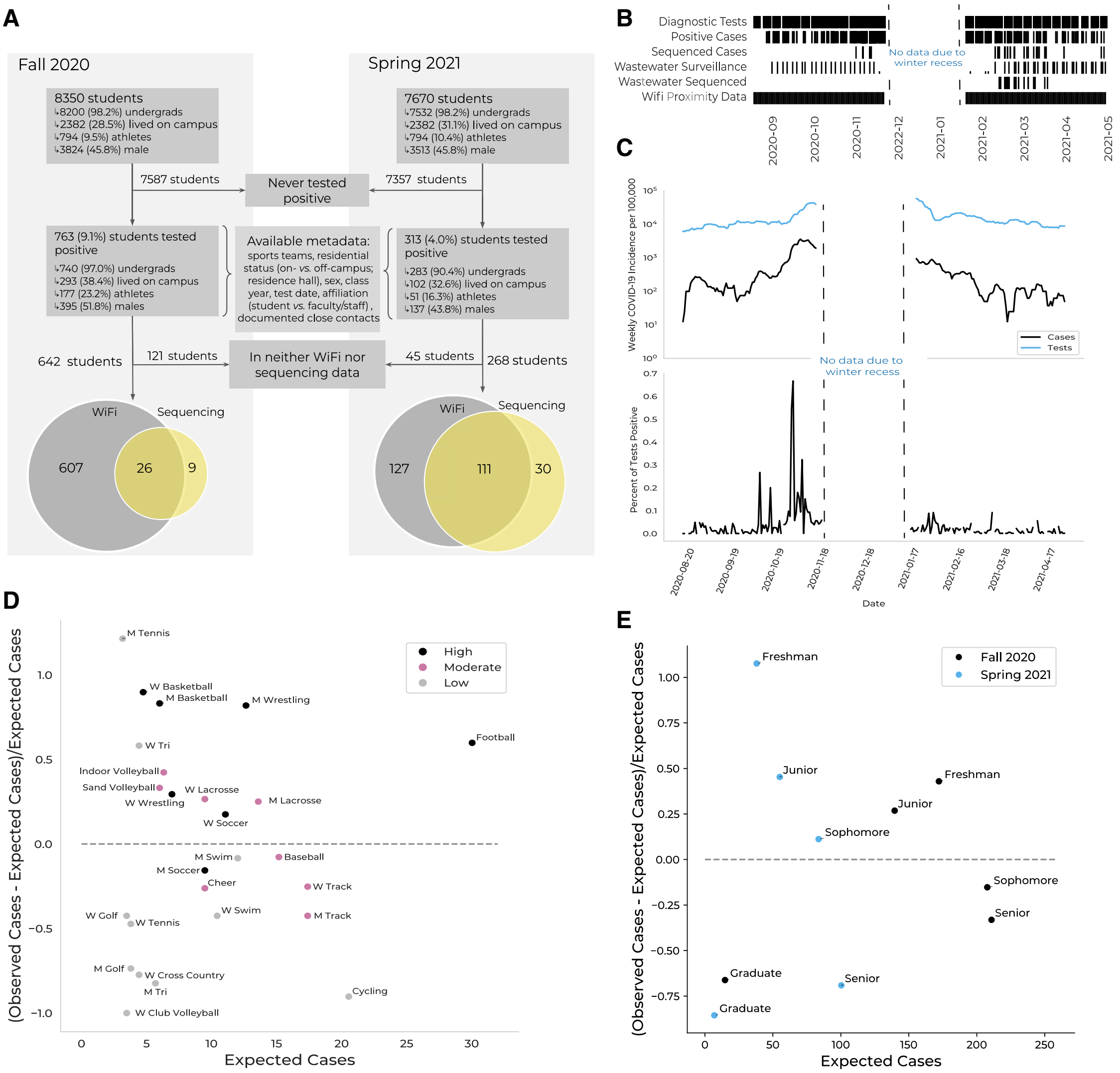

Figure 2. Data types, incidence rates, and epidemiological risk factors for SARS-CoV-2 positivity on Colorado Mesa University's campus

(A) Cohort description. A subset of students at Colorado Mesa University (CMU) tested positive for COVID-19 via reflexive or random surveillance qRT-PCR testing. CMU provided demographic and behavioral metadata for each case. Most students who tested positive were enrolled in the WiFi proximity program (gray). Some positive samples were available for viral genomic sequencing (yellow).

(B) Data collection time points (black) by data type during the Fall and Spring semesters. Data not shown for November 21–January 18 due to winter recess.

(C) Upper: weekly COVID-19 incidence (black) and number of tests conducted (blue) over the 2020–2021 academic year. Lower: percent positivity rate. Data not shown for November 21–January 18 due to winter recess.

(D) The difference between the number of cases observed and the number of cases expected (based on sports team size and scaled by the number of cases expected; y axis) versus the number of cases expected (x axis) per sports team. The dashed line at y = 0 separates teams with more (above) or fewer (below) cases observed than expected. Teams are colored by contact level (legend). M refers to men's teams and W to women's teams.

(E) The difference between the number of cases observed and the number of cases expected (based on class size and scaled by the number of cases expected; y axis) versus the number of cases expected (x axis) per class year. The dashed line at y = 0 separates classes with more (above) or fewer (below) cases observed than expected. Classes are colored by semester (legend).

contacts, enabling subsequent detection of 93 distinct cases (8.4% of the total cases) within a week of the sentinel case's positive test. These efforts identified plausible transmission links; among pairs of sequenced cases identified via contact tracing, 79% had closely related genomes (with a genetic distance of at most two mutations), compared with 10% among randomly chosen sequenced pairs (Figure S2A).

Frequent wastewater surveillance enhanced reflexive testing, identifying SARS-CoV-2-positive residence halls whose residents were then randomly selected for follow-up testing. The effort captured effluent from ~75% (Fall) and ~85% (Spring) of the residential population. In response to spikes in viral titer, contributing residence halls were oversampled for testing; when warranted, up to half of a hall's residents were tested. The success of this program is reflected in the correlations between hall testing rates, which were primarily modulated by reflexive testing, and hall incidence rates (correlation = 0.60, $p = 0.04$), and between wastewater titers and contemporaneous case counts (correlation = 0.40, $p < 0.001$; Figures 5A, 5B, and Figure S3).

To assess the overall efficacy of CMU's surveillance program, we compared CMU's incidence rate to that of Mesa County, which had limited testing available at the time. CMU's weekly incidence exceeded county incidence rates and predicted them with a lag time of 3 days (correlation = 0.73; Figures S1A and S1B). This is consistent with reports that adequate university testing can foreshadow community outcomes[12] and highlights the ability of university testing programs to serve as bellwethers. As the pandemic's impact on the surrounding community became clearer, the university sponsored testing for external community members, both as a public benefit and to limit spread of SARS-CoV-2 into the campus.[31]

Epidemiological analyses identify student attributes associated with SARS-CoV-2 positivity and support a surveillance paradigm of targeted testing and risk mitigation

We identified risk factors among a wide range of institutionally captured attributes for individuals who tested positive: role (i.e., student or faculty/staff), sex, class year,[32] test date, association with a residence hall, and membership on a sports team. Residence halls and sports teams were annotated with features, including perceived contact risk for sports teams (Data S1). Our results support a two-pronged surveillance strategy, in which groups at increased risk are targeted for higher-cadence testing, while putatively causal factors are mitigated via institutional policies that reduce risk.

Athletes were 2.45 times as likely to test positive (Figure S1C), despite testing only 1.55 times as frequently as non-athletes. Meanwhile, on-campus students tested positive 1.30 times as often as their off-campus peers (Figure S1C), despite testing 1.80 times as often. Thus, sports participation was associated with increased risk of SARS-CoV-2 positivity, while residential living was not. Males, freshmen, and juniors also exhibited more cases than expected (Figures S1C and 2E; Data S1). These findings may underscore risk factors relevant for other universities, such as athletic participation, while emphasizing that policy can mitigate factors otherwise presumed to be risky, such as on-campus living.

Among sports teams, we identified specific attributes that predicted differences in case counts relative to team size. High-contact sports teams had increased incidence rates (Data S1), with 50% more cases than expected from the risk for athletes as a whole, while low-contact teams had 47% fewer (Figure 2D). We found no association between either sports location (i.e., indoor versus outdoor sports) and incidence rates or between sports testing and incidence rates (correlation = −0.05, $p = 0.81$), although sports played in both seasons had higher incidence rates than Fall or Spring sports (Data S1). These findings are consistent with a model where

individual athletes sporadically contract COVID-19, with an increased risk of further transmission and thus outbreaks on higher-contact teams or teams with longer seasons.

Because COVID-19 incidence rates varied from 9.7% to 27% across residence halls (Data S1), we conducted linear regression with multiple possible predictors to characterize factors that influenced incidence rates (Figures S4A and S4B). Two features were significant predictors: percent occupancy (i.e., percent of available beds filled) and private (versus hallway) bathrooms (see Figures S4C–S4E for model validation). For every increase of 10% in occupancy, our model predicted an increase of 0.015 in incidence, supporting institutional de-densification measures. Strikingly, halls with in-unit or private bathrooms were predicted to have an incidence 0.059 higher than those with hallway bathrooms, consistent with reports that a majority of SARS-CoV-2 transmissions occur within households (here, within suites).[33] Other possible explanations include compensatory protective measures (i.e., masking or social distancing) in larger bathrooms or increased hygiene of hallway bathrooms, which were cleaned by professional staff rather than residents. Importantly, our model does not account for possible social confounders such as clustering of certain groups (e.g., athletes) within specific residence halls.

Distinct interaction dynamics of positive individuals within WiFi proximity data reveal potential for digital contact tracing

We explored a dataset of anonymized daily logged connection locations (i.e., access point and building) for students connected to campus WiFi for at least 15 min and documented how such data can be extended for real-time disease surveillance. Data were obtained from a program implemented in 2018 to assess facility use and student engagement. Students were alerted about the program via a campus-wide notice and could opt out; 98% of students participated.

Through an examination of campus-wide connectivity patterns, we identified associations between student activity and CMU's COVID-19-related policies. We found elevated on-campus presence during weekdays (versus weekends) and in residence halls (versus other building types) in Fall 2020 (Figures S5, S6A, and S6C), reflecting university policies that discouraged on-campus gathering. When mitigation policies relaxed in Spring 2021, weekend presence increased relative to Fall 2020 (Figures S6B and S6D). Moreover, after testing positive, individuals had 42% fewer contacts than during the preceding 10 days, indicating adherence to isolation policies (Figure S7A). This quantification of policy adherence suggests that WiFi data can be used to assess policy implementation or to determine the effects of policy updates in real time.

We found that positive individuals exhibited distinct patterns in their social behaviors. Individuals who eventually tested positive exhibited larger social networks than those who remained negative: they spent more days on campus (Figure S7B), had more daily contacts (Figure 3A, left), and had longer interactions with each contact (Figure 3A, right), creating more opportunities for viral transmission. Furthermore, pairs of students identified via contact tracing had significantly longer interactions in the 10 days preceding their positive tests than other pairs of positive students (Figures S7C and S7D). These pairs of positive students (i.e., pairs where COVID-19 transmission may have occurred) interacted for significantly longer than pairs in which transmission did not or could not have occurred (i.e., pairs involving one or more students who never tested positive; Figures S7C and S7D). These patterns suggest that WiFi tracing can be harnessed to automatically flag close contacts

CellPress
OPEN ACCESS

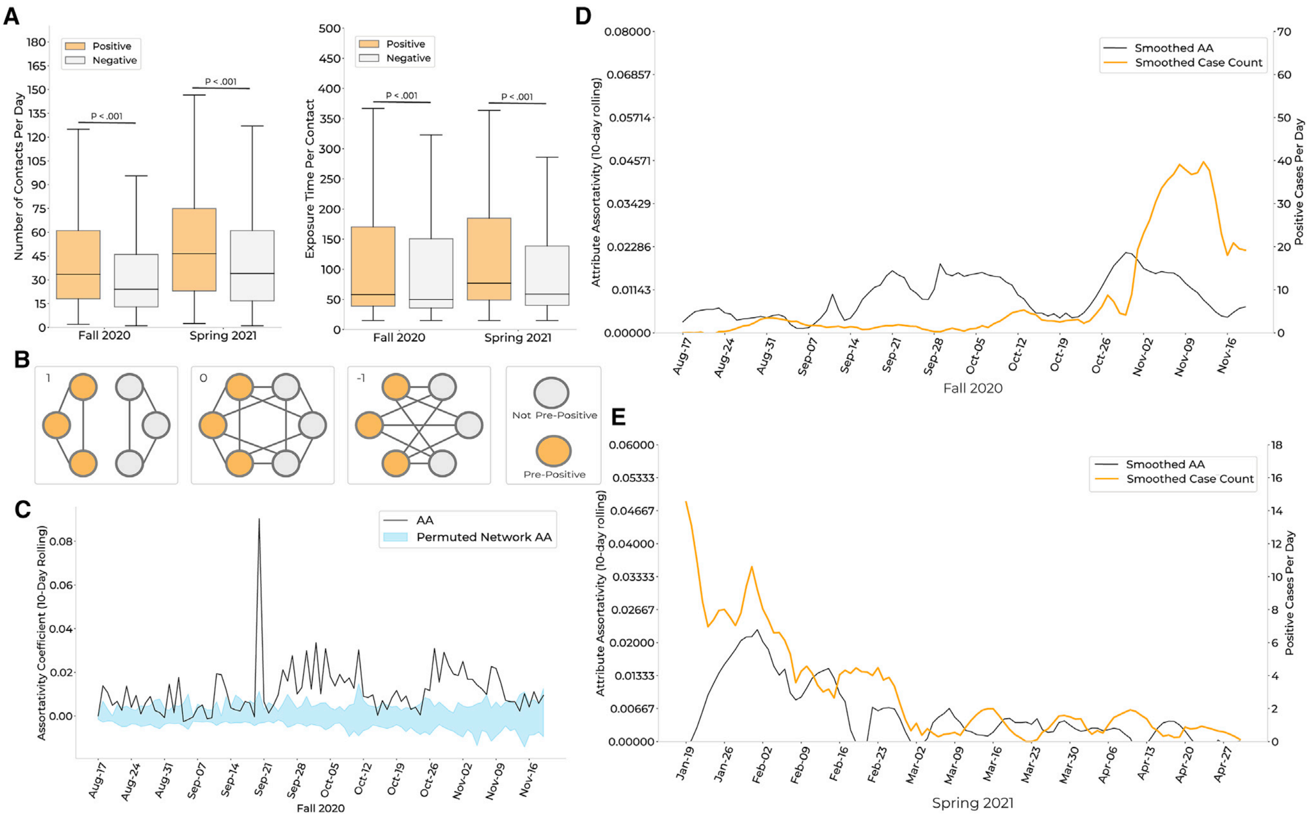

Figure 3. Social connectivity network inferred from the WiFi co-location data identifies behavioral trends that correlate with case counts

(A) Left: distributions of daily contacts for students who tested positive (orange) versus those who remained negative (gray) over the course of each semester. Right: distributions of average exposure time per contact, in minutes, for cases (orange) versus those who remained negative (gray). p values via Wilcoxon rank sum test.

(B) Visual representation of the network metric attribute assortativity (AA). Network scenarios where the AA coefficient is equal to 1, 0, or −1 are depicted. Positive AA values indicate a higher propensity for within-group interactions, while negative values indicate a higher propensity for between-group interactions.

(C) Comparison of per-semester AA for individuals within a 10-day window of a positive test (i.e., pre-positives) versus those who never tested positive (i.e., negatives). Ninety-five percent confidence intervals (Cis) (blue) were calculated by permuting pre-positive and negative labels within the proximity network 40 times per day. The AA of the proximity network (black) was above the upper bound of the CI for 69.4% (66/95 days) of the Fall 2020 semester, implying significance at $p < 0.025$. Results were consistent for Spring 2021 (Figure S8).

(D and E) Relationship between smoothed case counts and smoothed AA for Fall 2020 (D) and Spring 2021 (E). Smoothing via the Savitzky-Golay filter (window length = 17, polynomial order = 4).

of infected individuals, supplementing or even substituting for manual contact tracing.

We further explored interactions between positive and negative individuals using attribute assortativity (AA), which quantifies the extent to which individuals interact within versus between groups (Figure 3B). We found that both positive (i.e., individuals who test positive during the semester) and pre-positive (i.e., individuals within 10 days of a positive test) individuals were more likely to associate with one another than with negative individuals (Figures 3C and S8A–S8C). This relationship remained significant when removing pre-positive individuals who identified one another as close contacts, suggesting that it is not biased by reflexive testing following manual contract tracing (Figure S9). Interestingly, the AA for pre-positive individuals was a leading indicator of daily case counts, by 8 days (Fall) and 3 days (Spring; Figures 3D, 3E, and S8D–S8F), suggesting that the degree of within-group interactions among infectious individuals increases in the days leading to these individuals' positive tests.

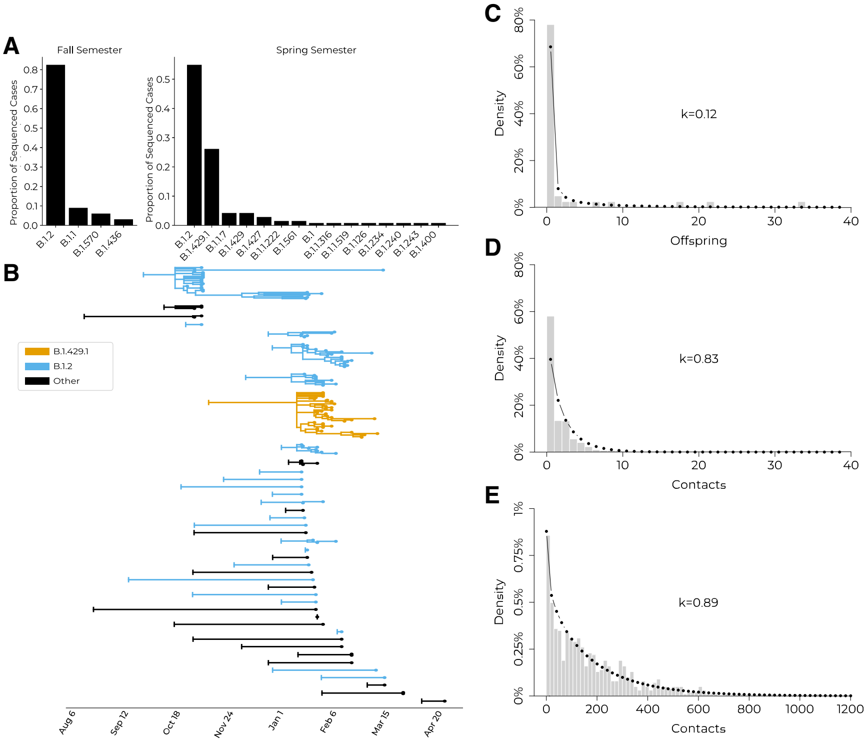

Figure 4. Viral genomic sequencing highlights case clusters, viral lineages, and phylogenomic overdispersion

(A) Pango lineage proportions for university cases during Fall 2020 and Spring 2021.

(B) Phylogenetic tree with branch lengths scaled to time. B.1.2 clusters (blue), B.1.429.1 cluster (orange), and all other lineages (black). Vertical bar on the left of each introduction indicates the inferred ancestral root date of each cluster; cases are tip dots at right of the tree.

(C) Distribution of phylogenetic offspring, with a negative binomial distribution fit (dotted line) to quantify overdispersion. Offspring were defined as all phylogenetic descendants of a single introduction to campus; mean = 2.56 offspring per introduction; k = 0.13 (95% CI, 0.04–0.21).

(D) Distribution of the number of contacts from positive individuals identified during contact tracing, with a negative binomial distribution fit (dotted line). Contacts were defined as individuals with interactions longer than 15 min in the 48-h period prior to positive test or symptom onset; mean = 1.71 contacts per positive individual; k = 0.83 (95% CI, 0.71–0.94).

(E) Distribution of the number of WiFi contacts observed from positive individuals, with a negative binomial distribution fit (dotted line). Contacts were defined as individuals with interaction durations greater than 15 min in the 48-h period prior to testing positive or symptom onset; mean = 177.94 contacts per positive individual; k = 0.89 (95% CI, 0.81–0.98).

Phylogenetic analysis of clinical viral genomes identifies cluster size overdispersion and cryptic transmissions, leading to concrete policy decisions

Viral genomic sequencing of residual biomaterial enabled exploration of transmission dynamics and monitoring of SARS-CoV-2 variants. At CMU, sequencing facilitated detection of 18 distinct Pango lineages (Figures 4A, S2B, and S2C).[34] B.1.2 was the most abundant lineage at CMU and in Colorado, reflecting circulation between CMU and the surrounding community and highlighting the importance of CMU's sponsored testing for Mesa County.[35] We identified continuous transmission of this lineage between semesters, with 7 Spring cases descending from 17 Fall cases as the result of an estimated 2–3 cryptic intermediate transmissions during winter recess.[36] This cluster was non-significantly enriched for off-campus students relative to the remaining sequenced cases (83% versus 70% off-campus; p = 0.15); possible off-campus continuation of the transmission chain over the break suggests

that institutional surveillance programs may benefit from maintaining testing availability during school breaks.

We detected overdispersion in both genomic and social clusters, highlighting the importance of policies that minimize superspreading events. Of 41 detected introductions to the university, onward transmission was only observed from 13, with 5 of the clusters containing 80% of sequenced cases (k = 0.12 in a negative binomial model, consistent with other studies[37,38]; Figures 4B and 4C). We also observed overdispersion in the number of contacts per individual using both contact tracing and WiFi proximity data, where 80% of reported contacts were made by 33% (k = 0.83) and 43% (k = 0.89) of positive individuals, respectively (Figures 4D and 4E). Notably, social overdispersion only accounted for 13%–14% (the ratio of social k^{-1} to phylogenomic k^{-1}) of the overdispersion in phylogenomic cluster size, emphasizing that overdispersion in SARS-CoV-2 transmission consists of both social and biological components. Below, we document the interplay between social and biological factors that influenced the expansion of a large case cluster.

Contemporaneous wastewater viral sequencing supplements lineage detection and enables detection of emergent mutations

During 6 weeks from February to mid-March 2021, we obtained 42 wastewater samples from 10 sites for sequencing; 9 samples were sequenced in duplicate (Figure S3C). The concurrent collection of wastewater samples and clinical specimens, with high breadth of coverage among the residential population, allowed us to directly compare viral sequences from wastewater with those from contemporaneous cases. We validated the utility of wastewater viral sequencing as a component of a comprehensive surveillance program, as currently instantiated by *Lookout*.

Wastewater viral titers were lower than titers of clinical specimens collected from upstream individuals (Figure S10A). We sequenced wastewater samples, which had similar sequence coverage to clinical samples from CMU, suggesting that there was no particular bias in viral RNA degradation in wastewater (Figures S10B–S10E). We used the Freyja tool[39,40] to detect eight lineages in wastewater, three of which were found in concurrent clinical cases (Figure 5C; Data S1). Another three were observed in clinical cases prior to wastewater collection, suggesting undetected campus circulation, shedding from previously infected individuals, or environmental persistence. The remaining two, B.1.533 and B.1.350, were present in the US but not the campus or state[35]; each was detected at low abundance in a single sample and may have originated from a single individual. Wastewater sequencing thus identified lineages not concurrently detected via clinical sequencing, demonstrating particular relevance in instances of incomplete clinical genomic sampling.

In addition to detection of defined lineages, wastewater sequencing can also identify novel mutations; for this latter use case, we found that quality control mechanisms were essential to identify true variation. Of 1,521 wastewater single-nucleotide variants (SNVs), 85% and 68% were not found in consensus genomes from CMU and Colorado clinical samples, respectively, and only 4% were derived from clinical minor alleles (Figure 5D). We thus hypothesized that many mutations arose from sequencing or amplification errors, a theory supported by the order-of-magnitude difference in the number of SNVs detected in wastewater versus clinical samples as a function of sample count (Figure S11). We subsequently developed quality control methods to corroborate mutations via detection in state-wide clinical genomes. We achieved high specificity for discarding SNVs not seen in Colorado

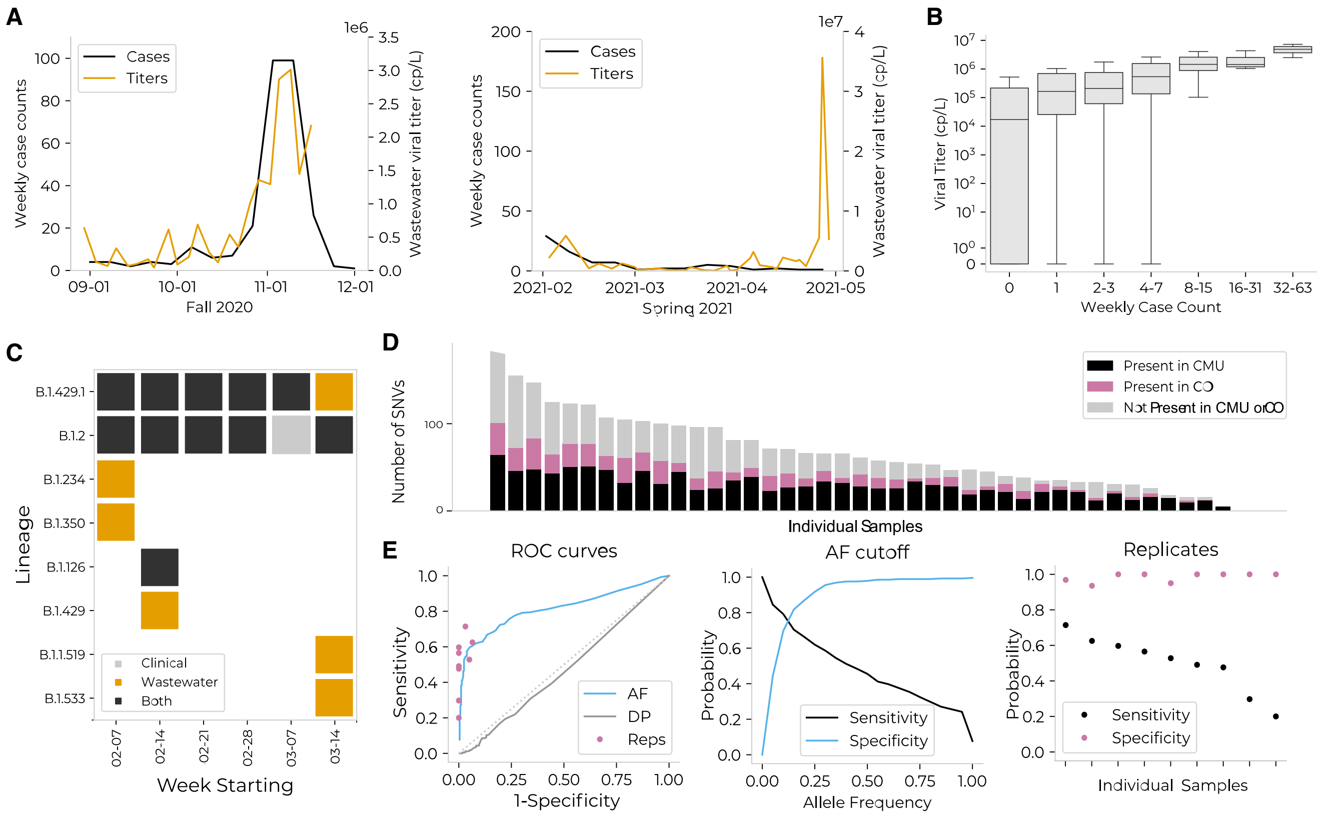

Figure 5. Wastewater surveillance and sequencing measures aggregate viral load, identifies circulating lineages, and parallels viral genomes from contemporaneous clinical cases

(A) Average wastewater viral titers (orange) versus weekly residential case count (black). Residential case counts were calculated relative to the subsets of dorms monitored (75% of residential population in Fall 2020; 99% in Spring 2021). There was an anomalous peak in wastewater viral titer observed in April, which may be due to technical error, differential shedding patterns, or undiscovered positive individuals.

(B) Viral titer (y axis) versus binned weekly case count (x axis; binned by powers of 2) for each wastewater sample. Viral titer and case count were significantly associated via Fisher's exact test (on binned slopes; $p = 0.04$) and Spearman's correlation = 0.40 ($p < 0.001$).

(C) Lineages detected on campus via wastewater or clinical sequencing.

(D) The number of single-nucleotide variants (SNVs) detected in wastewater samples; each bar represents a single sample. Individual samples are organized on the x axis in order of total number of SNVs. For each sample, SNVs are categorized by whether they were present in clinical sequences from CMU (black), in clinical sequences from Colorado (pink), or in neither (gray). On average, 51% of SNVs in a single wastewater sample were not found in CMU clinical samples, and 36% were not found in Colorado clinical samples.

(E) Comparison of quality control methods to remove SNVs not validated by presence in Colorado clinical sequences. The three methods compared are: (1) allele frequency (AF): discarding SNVs present at an allele frequency below a given threshold; (2) read depth (DP): discarding SNVs located at a site with a read depth below a given threshold; and (3) replicates (Reps): discarding SNVs not present within both of two technical replicates of a given sample. These analyses are limited to the nine samples for which technical replicates exist. Left: ROC curves for each of the three filters. Middle: sensitivity and specificity for allele frequency-based quality control method. Right: sensitivity and specificity for replicate-based quality control method.

when we required presence in both of two technical replicates (specificity = 98%) or an allele frequency exceeding 25% (specificity = 92%); both methods had low sensitivity (50% and 62%, respectively; Figure 5E), as each excluded SNVs corroborated by clinical viral genomes. This analysis provides real-word evidence of the importance of replicates for identifying true SNVs in wastewater samples, a finding previously shown for clinical minor allele validation.[41]

Of 68 replicate-corroborated SNVs found across the 9 wastewater samples, 11 (16%) were not seen in clinical CMU samples (Data S1). Six of the 11 were present in Colorado and had allele frequencies >96% in single wastewater specimens, likely reflecting on-campus circulation of viral genotypes unsampled by clinical sequencing.

Of the five remaining mutations, two were non-synonymous mutations in ORF1ab (I1970S, T3462I) and were novel compared with published global variation,[35] two were synonymous mutations, and one was a premature stop codon. The latter mutation, with an allele frequency of 4%, may be spurious; the other four, with allele frequencies between 27% and 100%, could reflect either gut tropism or cryptic transmission. Although these mutations' phenotypic effects remain unknown, their identification serves as a proof-of-concept and provides a framework for the detection of novel mutations in wastewater.

Detection of novel lineage B.1.429.1 on campus leads to high-resolution characterization of social and biological factors implicated in its spread

In Spring 2021, we detected a cluster of cases that was concerning due to its unprecedented size and genomic ancestry; we proceeded to characterize it analytically and experimentally to identify the social and biological factors that contributed to its spread. This B.1.429.1 cluster resulted from a single introduction to campus, which proliferated into several star-like descendant sub-clusters, consistent with clonal amplification (Figure 6A). In total, the outbreak lasted for 45 days; in its final 4 weeks, it represented 33% of sequenced clinical samples and was the most abundant lineage in 47% of wastewater samples (Figures 6A and 6B). B.1.429.1 descended from B.1.429—then deemed a variant of concern (VoC) due to reduced antibody neutralization and increased viral shedding, infectivity, and transmissibility[42]—and also included the recurrent S:Q677H substitution, posited to further increase transmissibility[43] (Data S1).

We integrated WiFi and genomic data to investigate whether social or biological factors were driving the spread of B.1.429.1. While B.1.429.1-infected individuals trended toward having more contacts than those contemporaneously infected with other viral lineages, the results were nonsignificant (Figure S12A); moreover, they displayed no differences in interaction durations (Figure S12B). Thus, we hypothesized that B.1.429.1's expansion was at least in part due to inherent qualities of the lineage rather than the social dynamics of the individuals within the cluster. We did find that B.1.429.1-infected individuals clustered together in social networks (Figure 6C); i.e., they were on average one social connection closer to one another than to other positive individuals. WiFi-connected B.1.429.1 pairs also had significantly lower viral genetic distances than non-connected B.1.429.1 pairs (Figure 6D), demonstrating that connections observed in the WiFi network include plausible transmission events.

We inferred direct transmission links among B.1.429.1 cases and found that WiFi-inferred transmission networks paralleled those constructed with traditional contact tracing data. Alone, manual contact tracing and genomic sequencing resolved transmission links for 61% and 68% of individuals, respectively (Figures S12D and S12E). Thus, we combined genomic data with traditional (2 days before tests) or WiFi-derived (2 or 10 days before tests) contact tracing, producing transmission models connecting 82%, 87%, and 74% of sequenced cases, respectively (Figures 6E, 6F, and S12F). We compared the cluster topology of these networks (via Jaccard distance; Data S1) and found that the WiFi 10-day data best approximated the traditional contact tracing data in transmission reconstruction. Due to the paucity of distinguishing mutations present between individual consensus sequences,[44] we used intrahost viral variation to supplement our transmission links. We identified a clear transmission chain where a single mutation present at low frequency in one specimen (no. 26 in Figures 6E and 6F) reached fixation in two specimens (nos. 27 and 28 in Figures 6E and 6F) collected 1 week later, consistent with bottlenecked transmission. These three individuals clustered together in all reconstruction networks, but without

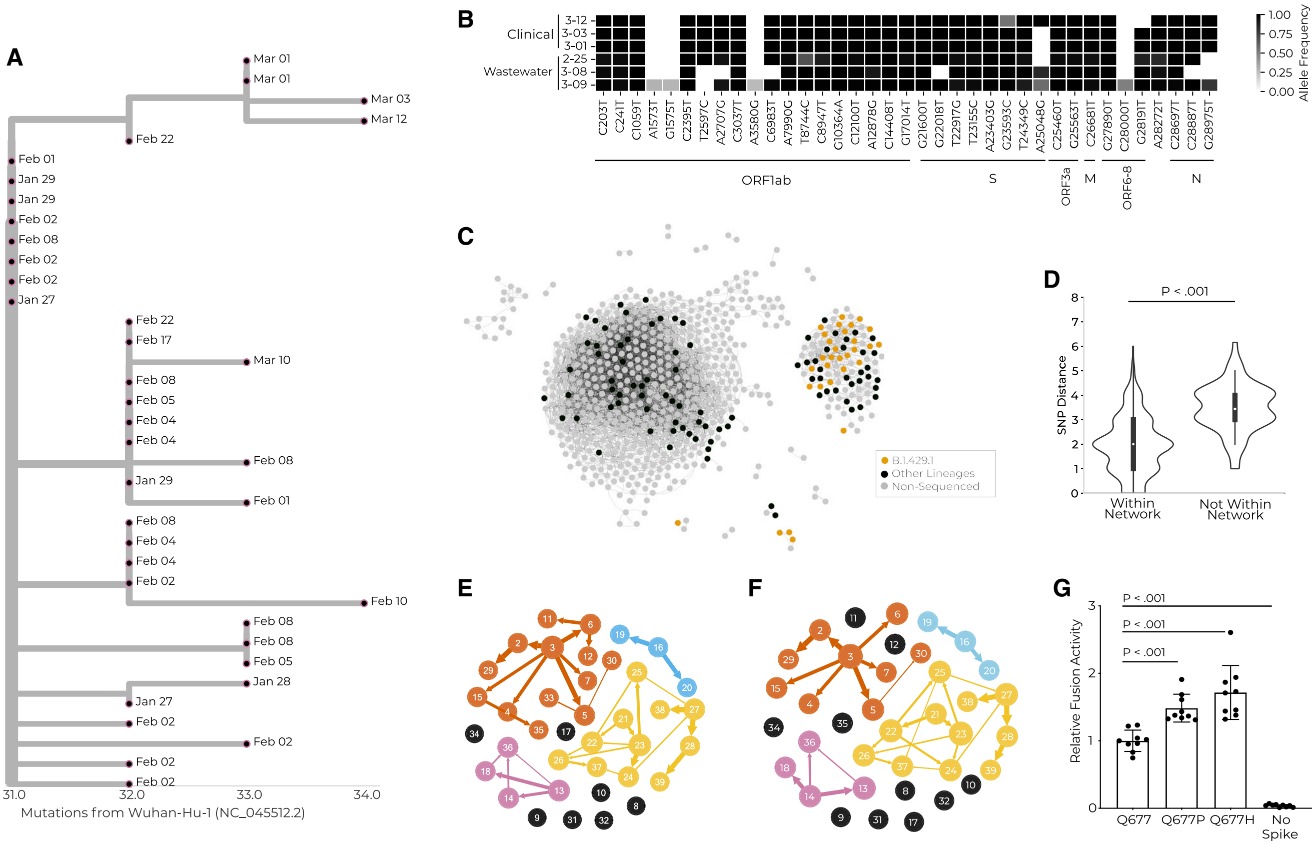

Figure 6. A multimodal exploration of the novel lineage B.1.429.1 via clinical and environmental genomic sequencing, WiFi proximity analyses, transmission reconstruction networks, and experimental validation

(A) Phylogenetic tree showing the relationship between cases within the B.1.429.1 case cluster. Tree tips are anchored at their dates of sample collection, and branch lengths are scaled by maximum likelihood.

(B) Three wastewater samples and three clinical samples (y axis), all of the B.1.429.1 lineage. The three wastewater samples had B.1.429.1 as the sole identified lineage, and were extracted from site 3, for which residential halls B and M were the only upstream contributors. Clinical viral genomes from three students believed to have contributed effluent to these wastewater samples, based on residential status and test date, are shown. The x axis represents all SNVs present in at least one wastewater sample with 25% allele frequency or greater. SNVs are grouped by genomic position.

(C) Social proximity network for interactions occurring between putatively infectious individuals. Edges represent one or more simultaneous WiFi access point connections between two individuals within 10 days of their positive tests. Each node represents a positive individual. The node color represents their sequencing status (legend).

(D) Genetic distance for individuals infected with the B.1.429.1 lineage who are or are not connected via the social proximity network shown in (C). Effect size = 1 mutation. p value via rank sum test.

(E and F) Transmission reconstruction network for B.1.429.1 cases created with genomic information as well as manual (E) or WiFi-inferred 10-day (F) contact tracing data.

(G) Cell-cell fusion activity of viral pseudotypes with the ancestral allele, or with the S:Q677P or S:Q677H amino acid changes, relative to a luminescent control with no Spike protein expressed.

the transmission direction inferred from minor alleles or phylogenetic descent, implying that transmission network reconstruction tools require further refinement.

Finally, we studied viral phenotypic factors that could explain the increased transmission of B.1.429.1 on campus. We assessed the impact of the S:Q677H mutation found in B.1.429.1 on single-cycle infectivity and on cell-to-cell fusogenicity in lentiviral pseudotypes (Figures S12G and S12H). While the mutation did not alter cell-free virion infectivity (Figure S12C), it significantly increased fusion efficiency relative to the ancestral B.1.429 spike protein (Figure 6G), likely due to its proximity to the protein's polybasic cleavage site. The S:Q677P mutation, which was detected in contemporaneous CMU lineages, had a similar phenotype. This finding is consistent

with a phenotypic advantage among SARS-CoV-2 haplotypes bearing S:Q677H or S:Q677P.

Fortunately, B.1.429.1 was minimally detected outside the campus, pointing to the success of CMU's containment policies. This vignette highlights the power of systematic, multimodal surveillance programs to not only identify and mitigate transmission events, but to also contribute to novel biological characterization of viral lineages.

DISCUSSION

Here, we analyzed clinical diagnostic data, case attributes, WiFi co-location logs, wastewater samples, and viral genomic sequences to assess CMU's pandemic response program and to determine the relevance of each data type to infectious disease surveillance. Our analyses showed that CMU effectively identified positive cases through contact tracing, wastewater surveillance, and increased focus on high-risk groups. Via analyses of WiFi connectivity data, we confirmed adherence to school policies and evaluated the ability of WiFi data to replace or supplement traditional contact tracing. In addition, we leveraged phylogenetic and epidemiological analyses to propose future policies to limit disease spread (e.g., continued testing during school breaks and risk prediction for testing prioritization) and to identify and mitigate specific factors associated with increased risk (e.g., requiring masking or increased testing to participate in high-contact sports). Our sequencing of wastewater samples not only identified lineages independently of clinical sequencing, but also allowed us to evaluate methods necessary for the detection of novel mutations in wastewater. Finally, through analysis of case cluster overdispersion and the novel lineage B.1.429.1, we highlighted the relevance of investigating both virological and sociobehavioral factors that can influence transmission.

Importantly, we used some data types to inform policy in near real time. Risk analyses and contact tracing data suggested that spread primarily occurred in social groups, such as in high-contact sports teams; CMU thus invited students to register in social units in Spring 2021, where they could associate freely but were subject to reflexive testing in the case of a positive test. Moreover, students were alerted to the presence of a VoC on campus 1–3 weeks after the lineage's arrival, including time for viral genomic sequencing, bioinformatics analysis, and the creation of straightforward and transparent messaging that re-emphasized public health protocols. On the other hand, wastewater sequencing and WiFi connectivity data were analyzed retrospectively, but demonstrate the utility of these tools in future prospective surveillance programs.

Our results lead us to formulate a framework combining the analyzed tools within an integrated disease surveillance system (Figure 7). We emphasize beginning with symptom reporting, contact tracing, and isolation of infected individuals, and continuing with efficient testing strategies, such as wastewater surveillance. While contact tracing is essential, it is also time intensive and expensive to maintain; with further research, WiFi proximity and geolocation data could potentially replace these efforts. Gathering epidemiological metadata, symptom attestations, and diagnostic test results digitally and with programmatic synthesis in mind is also a high priority because it can facilitate real-time analyses and subsequent policy adjustments; the *Lookout* system serves as a useful template (Figure 1).[30] If finances allow, we suggest adding genomic surveillance to identify transmission patterns and concerning lineages or mutations. For communities with wastewater surveillance, sequencing these samples provides a cheaper alternative to clinical sequencing of all upstream individuals and enables identification of lineages or mutations of interest. This tool cannot wholly replace clinical sequencing

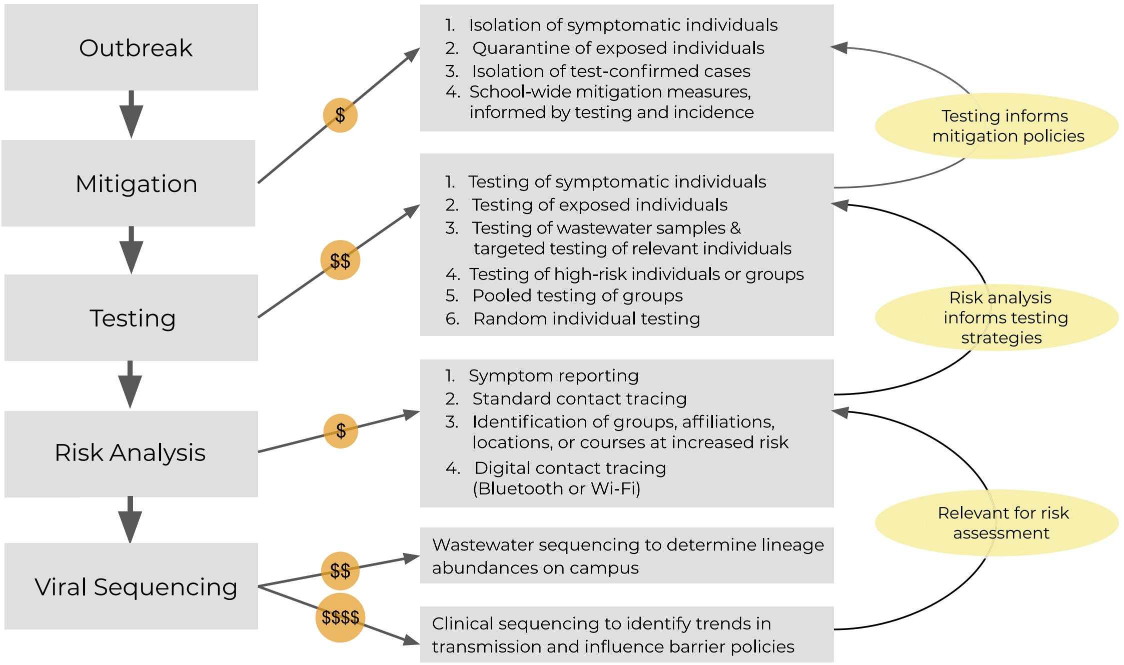

Figure 7. A stepwise approach to outbreak surveillance with consideration of resource limitations
The actions to employ during an institutional outbreak, with delineation of relative cost and information feedback cycles. During an outbreak, initial mitigation measures can be deployed prior to and independent of a surveillance program. A basic surveillance program will first incorporate testing, the results of which will inform additional mitigation policies. Next, analyses of case attributes can be used to assess the risk of infection for specific sub-populations; these analyses will allow for development of specialized, directed testing strategies. Finally, while more expensive, viral genomic sequencing of clinical or environmental samples can be used to identify transmission trends and to detect emergent viral genomic variation with potential public health or clinical relevance. This can be used to inform institutional policy and mitigation efforts. Actions involving solely personnel time are the least expensive to implement (i.e., mitigation, risk analyses), while actions requiring both personnel and laboratory consumables are more expensive (i.e., testing), and actions requiring highly trained personnel, laboratory consumables, and prolonged instrument time are the most expensive to implement (i.e., viral sequencing).

due to its inability to discern transmission trends. It is important to emphasize that disease surveillance is not a one-size-fits-all endeavor; in fact, we found parallel results across data types. We suggest that the automated integration of a subset of data types will more powerfully combat infectious disease outbreaks than a siloed implementation of all data types.

Limitations of the study

Our findings are subject to methodological and policy-based limitations. As with all studies of infectious disease surveillance, transmission events and clustering can violate statistical assumptions of independence among individuals. In addition, we could not separate the impact that individual attributes (e.g., a particular sports team or residence hall) had on risk of infection due to lack of data on the overlap between these attributes. Moreover, incomplete sampling of residual diagnostic and wastewater samples limited us to a partial snapshot of SARS-CoV-2 genetic diversity at CMU (Figures 2A and S3C). Furthermore, WiFi co-location data remain underexplored and do not capture off-campus interactions. As our study largely took place prior to the widespread availability of SARS-CoV-2 vaccines[45] and rapid antigen tests,[46] we cannot assess their impact on transmission or policy. Furthermore, there are barriers to the implementation and execution of these surveillance approaches, particularly for approaches with greater novelty (Data S1). Finally, CMU's surveillance paradigm prioritized community safety over individual privacy; thus, some of our findings may not be generalizable to institutions with different prioritizations.

Accounting for resource constraints, we built upon CMU's community-driven mindset to develop an efficient surveillance program and lay the groundwork for future advances. While a number of analyses here were conducted retrospectively, updates to surveillance software, such as *Lookout*,[30] could enable timely identification of risk factors, proximity and location patterns, and lineages or mutations that are rising in frequency or that have been categorized as VoCs. Moreover, this tool can be adapted for use with other seasonal (e.g., influenza) or emerging pathogens. These programs can further refine outbreak reconstruction tools by incorporating genomic data (including major and minor alleles) and contact tracing (obtained from manual efforts or WiFi analyses), with reported contacts weighted by the length or nature of the interaction. In summary, we propose the automated integration of multiple data types as the most powerful way to combat infectious disease outbreaks as they unfold.

STAR★METHODS

Detailed methods are provided in the online version of this paper and include the following:

- KEY RESOURCES TABLE
- RESOURCE AVAILABILITY
  - Lead contact
  - Materials availability
  - Data and code availability
- EXPERIMENTAL MODEL AND SUBJECT DETAILS
  - Ethics statement
  - Cell culture
- METHOD DETAILS
  - Wastewater collection and quantification
  - Viral genomic sequencing
  - Functional characterization of spike glycoprotein mutations
- QUANTIFICATION AND STATISTICAL ANALYSIS
  - Epidemiological modeling
  - WiFi analyses
  - Wastewater analyses
  - Viral genomic analyses

SUPPLEMENTAL INFORMATION

Supplemental information can be found online at https://doi.org/10.1016/j.medj.2022.09.003.

ACKNOWLEDGMENTS

This work was supported by the National Science Foundation (NSF) Graduate Research Fellowship Program to J.S.P. (grant no. 1745303), and C.H.T.-T. (grant no. 1745303); the Hertz Foundation to J.S.P.; the National Institute of General Medical Sciences to B.A.P. (grant no. T32GM007753); the National Science Foundation (NSF) to B.C.L. (grant no. 2046440); the Moore Foundation to B.C.L., the National Institute of Allergy and Infectious Diseases to B.C.L. (grant no. 2U19AI110818) and P.C.S. (grant nos. U19AI110818 and U01AI151812); the National Institutes of Health to J.L. (grant nos. R37AI147868 and R01AI148784); the Massachusetts Consortium on Pathogen Readiness to J.L.; the Centers for Disease Control and Prevention (CDC) COVID-19 baseline genomic surveillance contract to the Clinical Research Sequencing Platform (grant no. 75D30121C10501) and a CDC Broad Agency Announcement to B.L.M. (grant no. 75D30120C09605); and the Howard Hughes Medical Institute to P.C.S. This work is also made possible by support from Flu Lab and a cohort of donors through the Audacious Project, a collaborative

funding initiative housed at TED, including The ELMA Foundation, MacKenzie Scott, the Skoll Foundation, and Open Philanthropy. Any opinion, findings, and conclusions or recommendations expressed in this material are those of the author(s) and do not necessarily reflect the views of the National Science Foundation. This content is solely the responsibility of the authors and does not necessarily represent the official views of the National Institute of General Medical Sciences or the National Institutes of Health. The authors wish to thank Jacob E. Lemieux for helpful discussions and feedback. The authors also wish to thank Laurent Hébert-Dufresne for providing insights and feedback on the WiFi co-location analyses.

AUTHOR CONTRIBUTIONS

Conceptualization, B.A.P., J.S.P., C.H.T.-T., B.C.L., E.B., C.B., K.K.B., A.C., B.F., B.L.M., A.Z.O., E.P., K.S., J.L., D.J.P., J.M., A.B., S.F.S., and P.C.S.; methodology, B.A.P., J.S.P., C.H.T.-T., B.C.L., L.Y., T.N., G.A., S.V.B., A.G.-Y., K.B.B., B.F., A.Z.O., K.S., K.J.S., J.L., D.J.P., A.B., and S.F.S.; software, B.A.P., J.S.P., C.H.T.-T., B.C.L., A.B., T.B.-F., P.C., O.G., C.L., L.M., K.O., M.S., B.F., and D.J.P.; validation, B.A.P., J.S.P., C.H.T.-T., B.C.L., A.B., M.S., A.Z.O., K.S., and S.F.S.; formal analysis, B.A.P., J.S.P., C.H.T.-T., B.C.L., T.B.-F., L.Y., S.M., T.N., G.K.M., I.S., A.D., and B.F.; investigation, B.A.P., J.S.P., C.H.T.-T., B.C.L., K.C.D., T.B.-F., L.Y., S.M., T.N., G.K.M., I.S., G.A., S.V.B., A.D., A.G.-Y., A.K., C.K., C.L., K.J.M., L.P., M.S., K.K.B., B.F., A.Z.O., K.S., K.J.S., J.L., and D.J.P.; resources, B.A.P., J.S.P., B.C.L., K.C.D., G.L.G., A.B., M.H., K.S., J.T.B., J.B., A.R.B., L.C., V.D., M.D., O.G., H.D.H., T.V.H., C.L., T.T.M., J.M.M., M.J.S., G.M.T., M.E.V., K.K.B., B.F., K.S., J.L., J.M., A.B., and P.C.S.; data curation, B.A.P., J.S.P., C.H.T.-T., B.C.L., G.L.G., A.B., M.H., E.L., J.B., B.F., C.L., M.S., A.T., B.F., A.Z.O., K.S., and A.B.; writing – original draft, B.A.P., J.S.P., C.H.T.-T., B.C.L., P.N., G.L.G., T.B.-F., L.Y., A.Z.O., and K.S.; writing – review & editing, B.A.P., J.S.P., C.H.T.-T., B.C.L., P.N., G.L.G., L.Y., S.M., Y.B.-L., B.L.M., K.J.S., D.J.P., A.B., S.F.S., and P.C.S.; visualization, B.A.P., J.S.P., C.H.T.-T., B.C.L., P.N., T.B.-F., L.Y., T.N., T.B., P.C., O.G., L.M., K.O., M.F., B.F., and K.S.; supervision, B.A.P., C.H.T.-T., M.H., K.K.B., A.C., B.F., B.L.M., A.Z.O., K.S., K.J.S., J.L., D.J.P., J.M., A.B., S.F.S., and P.C.S.; project administration, K.C.S., G.L.G., M.H., Y.B.-L., O.G., K.K.B., B.F., B.L.M., A.Z.O., E.P., K.S., J.M., A.B., and P.C.S.; funding acquisition, B.L.M., J.L., D.J.P., J.M., A.B., and P.C.S. B.C.L. created the graphical abstract with BioRender.com. B.A.P., J.S.P., C.H.T.-T., B.C.L., and S.F.S. performed or oversaw statistical analyses. B.A.P., J.S.P., and C.H.T.-T. had unrestricted access to all data. All authors agreed to submit the manuscript, read and approved the final draft, and take full responsibility for its content, including the accuracy of the data and its statistical analysis.

DECLARATION OF INTERESTS

P.C.S. is a co-founder of, shareholder in, and scientific advisor to Sherlock Biosciences, Inc.; she is also a Board member of and shareholder in Danaher Corporation. P.C.S. has filed IP related to genome sequencing and analysis.

INCLUSION AND DIVERSITY

We support inclusive, diverse, and equitable conduct of research.

Received: August 23, 2022
Revised: September 6, 2022
Accepted: September 12, 2022
Published: September 19, 2022

REFERENCES

1. Shah, M., Quinlisk, P., Weigel, A., Riley, J., James, L., Patterson, J., Hickman, C., Rota, P.A., Stewart, R., Clemmons, N., et al. (2018). Mumps outbreak in a highly vaccinated university-affiliated setting before and after a measles-mumps-rubella vaccination campaign-Iowa, july 2015-may 2016. Clin. Infect. Dis. *66*, 81–88.

2. Harrison, L.H., Dwyer, D.M., Maples, C.T., and Billmann, L. (1999). Risk of meningococcal infection in college students. JAMA *281*, 1906–1910.

3. Delbos, V., Lemée, L., Bénichou, J., Berthelot, G., Taha, M.K., and Caron, F.; B14 STOP study group (2013). Meningococcal carriage during a clonal meningococcal B outbreak in France. Eur. J. Clin. Microbiol. Infect. Dis. *32*, 1451–1459.

4. MacLennan, J., Kafatos, G., Neal, K., Andrews, N., Cameron, J.C., Roberts, R., Evans, M.R., Cann, K., Baxter, D.N., Maiden, M.C.J., et al. (2006). Social behavior and meningococcal carriage in British teenagers. Emerg. Infect. Dis. *12*, 950–957.

5. Segaloff, H.E., Cole, D., Rosenblum, H.G., Lee, C.C., Morgan, C.N., Remington, P., Pitts, C., Kelly, P., Baggott, J., Bateman, A., et al. (2021). Risk factors for severe acute respiratory syndrome coronavirus 2 (SARS-CoV-2) infection and presence of anti-SARS-CoV-2 antibodies among university student dormitory residents, september-november 2020. Open Forum Infect. Dis. *8*, ofab405.

6. Vang, K.E., Krow-Lucal, E.R., James, A.E., Cima, M.J., Kothari, A., Zohoori, N., Porter, A., and Campbell, E.M. (2021). Participation in fraternity and sorority activities and the spread of COVID-19 among residential university communities - Arkansas, August 21-september 5, 2020. MMWR Morb. Mortal. Wkly. Rep. *70*, 20–23.

7. Nolan, T., O'Ryan, M., Wassil, J., Abitbol, V., and Dull, P. (2015). Vaccination with a multicomponent meningococcal B vaccine in prevention of disease in adolescents and young adults. Vaccine *33*, 4437–4445.

8. Prater, K.J., Fortuna, C.A., McGill, J.L., Brandeberry, M.S., Stone, A.R., and Lu, X. (2016). Poor hand hygiene by college students linked to more occurrences of infectious diseases, medical visits, and absence from classes. Am. J. Infect. Control *44*, 66–70.

9. Lu, H., Weintz, C., Pace, J., Indana, D., Linka, K., and Kuhl, E. (2021). Are college campuses superspreaders? A data-driven modeling study. Comput. Methods Biomech. Biomed. Eng. *24*, 1136–1145.

10. Valesano, A.L., Fitzsimmons, W.J., Blair, C.N., Woods, R.J., Gilbert, J., Rudnik, D., Mortenson, L., Friedrich, T.C., O'Connor, D.H., MacCannell, D.R., et al. (2021). SARS-CoV-2 genomic surveillance reveals little spread from a large university campus to the surrounding community. Open Forum Infect. Dis. *8*, ofab518.

11. Crowe, J., Schnaubelt, A.T., Schmidt-Bonne, S., Angell, K., Bai, J., Eske, T., Nicklin, M., Pratt, C., White, B., Crotts-Hannibal, B., and Staffend, N. (2021). Pilot program for test-based SARS-CoV-2 screening and environmental monitoring in an urban public school district. Preprint at bioRxiv. https://doi.org/10.1101/2021.04.14.21255036.

12. Petros, B.A., Turcinovic, J., Welch, N.L., White, L.F., Kolaczyk, E.D., Bauer, M.R., Cleary, M., Dobbins, S.T., Doucette-Stamm, L., Gore, M., et al. (2022). Early introduction and rise of the Omicron SARS-CoV-2 variant in highly vaccinated university populations. Clin. Infect. Dis. ciac413. https://doi.org/10.1093/cid/ciac413.

13. Rennert, L., and McMahan, C. (2022). Risk of severe acute respiratory syndrome coronavirus 2 (SARS-CoV-2) reinfection in a university student population. Clin. Infect. Dis. *74*, 719–722. https://doi.org/10.1093/cid/ciab454.

14. Paltiel, A.D., Zheng, A., and Walensky, R.P. (2020). Assessment of SARS-CoV-2 screening strategies to permit the safe reopening of college campuses in the United States. JAMA Netw. Open *3*, e2016818.

15. Stubbs, C.W., Springer, M., and Thomas, T.S. (2020). The impacts of testing cadence, mode of instruction, and student density on fall 2020 covid-19 rates on campus. Preprint at medRxiv. https://doi.org/10.1101/2020.12.08.20244574.

16. Frazier, P.I., Cashore, J.M., Duan, N., Henderson, S.G., Janmohamed, A., Liu, B., Shmoys, D.B., Wan, J., and Zhang, Y. (2022). Modeling for COVID-19 college reopening decisions: cornell, a case study. Proc. Natl. Acad. Sci. USA *119*. e2112532119.

17. Queen, C.S., and Allen, J. (2021). 100 U.S. Colleges Will Require Vaccinations to Attend In-Person Classes in the Fall (The New York Times).

18. Karthikeyan, S., Nguyen, A., McDonald, D., Zong, Y., Ronquillo, N., Ren, J., Zou, J., Farmer, S., Humphrey, G., Henderson, D., et al. (2021). Rapid, large-scale wastewater surveillance and automated reporting system enable early detection of nearly 85% of COVID-19 cases on a university campus. mSystems *6*, e0079321.

19. Shah, M., Ferra, G., Fitzgerald, S., Barreira, P.J., Sabeti, P.C., and Colubri, A. (2022). Containing the spread of mumps on college campuses. R. Soc. Open Sci. *9*, 210948.

20. Sabeti, P., and Botti-Lodovico, Y. (2020). A Mumps Epidemic Has a Lot to Teach Colleges about Reopening Safely in the Time of Coronavirus. STAT. https://www.statnews.com/2020/10/08/a-mumps-epidemic-has-a-lot-to-teach-colleges-about-reopening-safely-in-the-time-of-coronavirus/.

21. Currie, D.W., Moreno, G.K., Delahoy, M.J., Pray, I.W., Jovaag, A., Braun, K.M., Cole, D., Shechter, T., Fajardo, G.C., Griggs, C., et al. (2021). Interventions to disrupt coronavirus disease transmission at a university, Wisconsin, USA, August-October 2020. Emerg. Infect. Dis. *27*, 2776–2785.

22. Matrajt, L., and Leung, T. (2020). Evaluating the effectiveness of social distancing interventions to delay or flatten the epidemic curve of coronavirus disease. Emerg. Infect. Dis. *26*, 1740–1748.

23. Kim, M.-C., Kweon, O.J., Lim, Y.K., Choi, S.H., Chung, J.W., and Lee, M.K. (2021). Impact of social distancing on the spread of common respiratory viruses during the coronavirus disease outbreak. PLoS One *16*, e0252963.

24. Glass, R.J., Glass, L.M., Beyeler, W.E., and Min, H.J. (2006). Targeted social distancing designs for pandemic influenza. Emerg. Infect. Dis. *12*, 1671–1681.

25. Tang, S., Xiao, Y., Yuan, L., Cheke, R.A., and Wu, J. (2012). Campus quarantine (Fengxiao) for curbing emergent infectious diseases: lessons from mitigating A/H1N1 in Xi'an, China. J. Theor. Biol. *295*, 47–58.

26. Harris-Lovett, S., Nelson, K.L., Beamer, P., Bischel, H.N., Bivins, A., Bruder, A., Butler, C., Camenisch, T.D., De Long, S.K., Karthikeyan, S., et al. (2021). Wastewater surveillance for SARS-CoV-2 on college campuses: initial efforts, lessons learned, and research needs. Int. J. Environ. Res. Publ. Health *18*, 4455.

27. Fielding-Miller, R., Karthikeyan, S., Gaines, T., Garfein, R.S., Salido, R.A., Cantu, V., Kohn, L., Wijaya, F.C., Flores, M., Omaleki, V., and Majnoonian, A. (2021). Wastewater and surface monitoring to detect COVID-19 in elementary school settings: the Safer at School Early Alert project. Preprint at medRxiv. https://doi.org/10.1101/2021.10.19.21265226.

28. Das Swain, V., Xie, J., Madan, M., Sargolzaei, S., Cai, J., De Choudhury, M., Abowd, G.D., Steimle, L.N., and Prakash, B.A. (2021). Empirical networks for localized COVID-19 interventions using WiFi infrastructure at university campuses. Preprint at bioRxiv. https://doi.org/10.1101/2021.03.16.21253662.

29. Malloy, M.L., Hartung, L., Wangen, S., and Banerjee, S. (2022). Network-side digital contact tracing on a large university campus. Preprint at arXiv. https://doi.org/10.48550/arXiv.2201.10641.

30. Anthes, E. (2021). The Future of Virus Tracking Can Be Found on This College Campus (The New York Times).

31. Specht, I., Sani, K., Botti-Lodovico, Y., Hughes, M., Heumann, K., Bronson, A., Marshall, J., Baron, E., Parrie, E., Glennon, O., et al. (2022). The case for altruism in institutional diagnostic testing. Sci. Rep. *12*, 1857.

32. Colorado Mesa University. (2020). Student Profiles (Colorado Mesa University). https://www.coloradomesa.edu/institutional-research/student-profiles/index.html.

33. Lei, H., Xu, X., Xiao, S., Wu, X., and Shu, Y. (2020). Household transmission of COVID-19-a systematic review and meta-analysis. J. Infect. *81*, 979–997.

34. O'Toole, Á., Scher, E., Underwood, A., Jackson, B., Hill, V., McCrone, J.T., Colquhoun, R., Ruis, C., Abu-Dahab, K., Taylor, B., and Yeats, C. (2021). Assignment of epidemiological lineages in an emerging pandemic using the pangolin tool. Virus Evol *7*, veab064.

35. B.1.429 Lineage Report. Karthik Gangavarapu, Alaa Abdel Latif, Julia Mullen, Manar Alkuzweny, Emory Hufbauer, Ginger Tsueng,

Emily Haag, Mark Zeller, Christine M. Aceves, Karina Zaiets, Marco Cano, Jerry Zhou, Zhongchao Qian, Rachel Sattler, Nathaniel L Matteson, Joshua I. Levy, Raphael TC Lee, Lucas Freitas, Sebastian Maurer-Stroh, GISAID core and curation team, Marc A. Suchard, Chunlei Wu, Andrew I. Su, Kristian G. Andersen, Laura D. Hughes, and the Center for Viral Systems Biology. outbreak.info, (available at https://outbreak.info/situation-reports?pango=B.1.429&loc=USA_US-NV&loc=USA_US-CA&selected=USA_US-NV). Accessed 29 September 2022.

36. Zhang, J., Litvinova, M., Wang, W., Wang, Y., Deng, X., Chen, X., Li, M., Zheng, W., Yi, L., Chen, X., et al. (2020). Evolving epidemiology and transmission dynamics of coronavirus disease 2019 outside Hubei province, China: a descriptive and modelling study. Lancet Infect. Dis. *20*, 793–802.

37. Meyerowitz, E.A., Richterman, A., Gandhi, R.T., and Sax, P.E. (2021). Transmission of SARS-CoV-2: a review of viral, host, and environmental factors. Ann. Intern. Med. *174*, 69–79.

38. Bi, Q., Wu, Y., Mei, S., Ye, C., Zou, X., Zhang, Z., Liu, X., Wei, L., Truelove, S.A., Zhang, T., et al. (2020). Epidemiology and transmission of COVID-19 in 391 cases and 1286 of their close contacts in Shenzhen, China: a retrospective cohort study. Lancet Infect. Dis. *20*, 911–919.

39. Freyja. Depth-Weighted De-mixing. (Github). https://github.com/andersen-lab/Freyja.

40. Karthikeyan, S., Levy, J.I., De Hoff, P., Humphrey, G., Birmingham, A., Jepsen, K., Farmer, S., Tubb, H.M., Valles, T., Tribelhorn, C.E., et al. (2021). Wastewater sequencing uncovers early, cryptic SARS-CoV-2 variant transmission. Preprint at medRxiv. https://doi.org/10.1101/2021.12.21.21268143.

41. Grubaugh, N.D., Gangavarapu, K., Quick, J., Matteson, N.L., De Jesus, J.G., Main, B.J., Tan, A.L., Paul, L.M., Brackney, D.E., Grewal, S., et al. (2019). An amplicon-based sequencing framework for accurately measuring intrahost virus diversity using PrimalSeq and iVar. Genome Biol. *20*, 8.

42. Deng, X., Garcia-Knight, M.A., Khalid, M.M., Servellita, V., Wang, C., Morris, M.K., Sotomayor-González, A., Glasner, D.R., Reyes, K.R., Gliwa, A.S., et al. (2021). Transmission, infectivity, and neutralization of a spike L452R SARS-CoV-2 variant. Cell *184*, 3426–3437.e8.

43. Hodcroft, E.B., Domman, D.B., Snyder, D.J., Oguntuyo, K.Y., Van Diest, M., Densmore, K.H., Schwalm, K.C., Femling, J., Carroll, J.L., Scott, R.S., and Whyte, M.M. (2021). Emergence in late 2020 of multiple lineages of SARS-CoV-2 Spike protein variants affecting amino acid position 677. Preprint at medRxiv. https://doi.org/10.1101/2021.02.12.21251658.

44. Wohl, S., Giles, J.R., and Lessler, J. (2021). Sample size calculation for phylogenetic case linkage. PLoS Comput. Biol. *17*, e1009182.

45. Office of the Commissioner. FDA Takes Key Action in Fight against COVID-19 by Issuing Emergency Use Authorization for First COVID-19 Vaccine. U.S. Food and Drug Administration. https://www.fda.gov/news-events/press-announcements/fda-takes-key-action-fight-against-covid-19-issuing-emergency-use-authorization-first-covid-19.

46. Center for Devices & Radiological Health. At-home OTC COVID-19 diagnostic tests. U.S. Food and Drug Administration. https://www.fda.gov/medical-devices/coronavirus-covid-19-and-medical-devices/home-otc-covid-19-diagnostic-tests.

47. Reeves, K., Liebig, J., Feula, A., Saldi, T., Lasda, E., Johnson, W., Lilienfeld, J., Maggi, J., Pulley, K., Wilkerson, P.J., and Real, B. (2021). High-resolution within-sewer SARS-CoV-2 surveillance facilitates informed intervention. Preprint at bioRxiv. https://doi.org/10.1101/2021.05.24.21257632.

48. van Elden, L.J.R., van Loon, A.M., van Alphen, F., Hendriksen, K.A.W., Hoepelman, A.I.M., van Kraaij, M.G.J., Oosterheert, J.J., Schipper, P., Schuurman, R., and Nijhuis, M. (2004). Frequent detection of human coronaviruses in clinical specimens from patients with respiratory tract infection by use of a novel real-time reverse-transcriptase polymerase chain reaction. J. Infect. Dis. *189*, 652–657.

49. Quick, J., Grubaugh, N.D., Pullan, S.T., Claro, I.M., Smith, A.D., Gangavarapu, K., Oliveira, G., Robles-Sikisaka, R., Rogers, T.F., Beutler, N.A., et al. (2017). Multiplex PCR method for MinION and Illumina sequencing of Zika and other virus genomes directly from clinical samples. Nat. Protoc. *12*, 1261–1276.

50. Betteridge, E., Park, N., James, K., Durham, J. & Quick, J. COVID-19 ARTIC v3 Illumina library construction and sequencing protocol - short amplicons (275bp) v1. protocols.io doi:10.17504/protocols.io.bh4zj8x6.

51. Tyson, J.R., James, P., Stoddart, D., Sparks, N., Wickenhagen, A., Hall, G., Choi, J.H., Lapointe, H., Kamelian, K., Smith, A.D., and Prystajecky, N. (2020). Improvements to the ARTIC multiplex PCR method for SARS-CoV-2 genome sequencing using nanopore. Preprint at bioRxiv. https://doi.org/10.1101/2020.09.04.283077.

52. Yurkovetskiy, L., Wang, X., Pascal, K.E., Tomkins-Tinch, C., Nyalile, T.P., Wang, Y., Baum, A., Diehl, W.E., Dauphin, A., Carbone, C., et al. (2020). Structural and functional analysis of the D614G SARS-CoV-2 spike protein variant. Cell *183*, 739–751.e8.

53. Katz, D., Baptista, J., Azen, S.P., and Pike, M.C. (1978). Obtaining confidence intervals for the risk ratio in cohort studies. Biometrics *34*, 469–474.

54. Newman, M.E.J. (2002). Assortative mixing in networks. Phys. Rev. Lett. *89*, 208701.

55. Proceedings of the python in science conference (SciPy): exploring network structure, dynamics, and function using NetworkX. http://conference.scipy.org/proceedings/SciPy2008/paper_2/.

56. Savitzky, A., and Golay, M.J.E. (1964). Smoothing and differentiation of data by simplified least squares procedures. Anal. Chem. *36*, 1627–1639.

57. Dijkstra, E.W. (1959). A note on two problems in connexion with graphs. Numer. Math. *1*, 269–271.

58. Auspice v2.38.0. https://nextstrain.org/groups/spheres/ncov/colorado. 2022.

59. Rambaut, A., Holmes, E.C., O'Toole, Á., Hill, V., McCrone, J.T., Ruis, C., du Plessis, L., and Pybus, O.G. (2020). A dynamic nomenclature proposal for SARS-CoV-2 lineages to assist genomic epidemiology. Nat. Microbiol. *5*, 1403–1407.

60. Hadfield, J., Megill, C., Bell, S.M., Huddleston, J., Potter, B., Callender, C., Sagulenko, P., Bedford, T., and Neher, R.A. (2018). Nextstrain: real-time tracking of pathogen evolution. Bioinformatics *34*, 4121–4123.

61. Sagulenko, P., Puller, V., and Neher, R.A. (2018). TreeTime: maximum-likelihood phylodynamic analysis. Virus Evol. *4*, vex042.

62. Minh, B.Q., Schmidt, H.A., Chernomor, O., Schrempf, D., Woodhams, M.D., von Haeseler, A., and Lanfear, R. (2020). IQ-TREE 2: new models and efficient methods for phylogenetic inference in the genomic era. Mol. Biol. Evol. *37*, 1530–1534.

63. Bedford, T., and Neher, R.. Overview of remote nCoV files (intermediate build assets) — SARS-CoV-2 Workflow documentation. https://docs.nextstrain.org/projects/ncov/en/latest/reference/remote_inputs.html?highlight=open.

64. Dudas, G. Baltic: Baltic - Backronymed Adaptable Lightweight Tree Import Code for Molecular Phylogeny Manipulation, Analysis and Visualisation. *Development is back on the evogytis/baltic branch (i.e. here).* (Github).

65. ProblematicSites_SARS-CoV2. (Github). https://github.com/W-L/ProblematicSites_SARS-CoV2. 2020.

66. Siddle, K.J., Krasilnikova, L.A., Moreno, G.K., Schaffner, S.F., Vostok, J., Fitzgerald, N.A., Lemieux, J.E., Barkas, N., Loreth, C., Specht, I., et al. (2022). Transmission from vaccinated individuals in a large SARS-CoV-2 Delta variant outbreak. Cell *185*, 485–492.e10.

STAR★METHODS

KEY RESOURCES TABLE

REAGENT or RESOURCE	SOURCE	IDENTIFIER
Antibodies		
SARS-CoV-2 (COVID-19) Spike S1 antibody [HL1]	GeneTex	#GTX635656; RRID: AB_2888549
SARS-CoV/SARS-CoV-2 (COVID-19) spike antibody [1A9]	GeneTex	#GTX632604; RRID: AB_2864418
Bacterial and virus strains		
Bovilis Coronavirus Calf Vaccine	Merk Animal Health	#16445
Biological samples		
Saliva specimens	CMU	N/A
Wastewater specimens	CMU	N/A
Chemicals, peptides, and recombinant proteins		
GlutaMAX Supplement	ThermoFisher Scientific	#35050061
TrypLE Express Enzyme (1X), no phenol red	ThermoFisher Scientific	#12604013
Nano-Glo Endurazine Live Cell Substrates	ProMega	#N2570
Critical commercial assays		
MycoAlert Mycoplasma Detection kit	Lonza	#LT07-318
QIAamp Viral RNA Mini Kit	Qiagen	#52904
QIAcuity One-Step Viral RT-PCR Kit	Qiagen	#1123145
MagMAX Viral RNA Isolation kit	ThermoFisher Scientific	#AM1939
NEBNext ARTIC SARS-CoV-2 FS Library Prep Kit	New England BioLabs	#E7658L
NovaSeq 6000 SP Reagent Kit v1.5 (300 cycles)	Illumina	#20028400
NovaSeq XP 2-Lane Kit v1.5	Illumina	#20043130
Illumina DNA Prep	Illumina	#20027213, #20027214, #20027216
NextSeq 500/550 Mid Output Kit v2.5 (300 Cycles)	Illumina	#20024905
TransIT-LT1 Transfection Reagent	Mirus	#MIR2304
Steady-Glo Luciferase Assay System	Promega	#E2510
Deposited data		
RNA sequencing data	NCBI SRA	BioProjects GenBank: PRJNA715749 and GenBank: PRJNA622837
GISAID consensus genomes	GISAID	
Genbank consensus genomes	NCBI Genbank	BioProjects GenBank: PRJNA715749 and GenBank: PRJNA622837
Plasmids	AddGene	Jeremy Luban Lab
Experimental models: Cell lines		
HEK 293T/17	ATCC	#CRL-11268
Oligonucleotides		
CDC SARS-CoV-2 primers (N1, N2, RP)	CDC	https://www.cdc.gov/coronavirus/2019-ncov/lab/rt-pcr-panel-primer-probes.html
BCoV forward and reverse primers (NOC43-1, NOC43-2)	Integrated DNA Technologies	https://doi.org/10.1086/381207
BCoV probe (NOC43-p) with HEX fluorophore and OQA quencher	Sigma-Aldrich	https://doi.org/10.1086/381207
ARTIC Network n-CoV-19 V3 primers	ARTIC Network	https://github.com/artic-network/artic-ncov2019/tree/master/primer_schemes/nCoV-2019/V3
Recombinant DNA		
HIV-1 pNL4-3ΔenvΔvpr luciferase reporter plasmid (pNL4-3.Luc.R-E-)	NIH AIDS Reagent Program	#3418
pcDNA3.1 SARS-CoV-2 S Epsilon Q677P	Addgene	#190463
pcDNA3.1 SARS-CoV-2 S Epsilon Q677H	Addgene	#190462
pcDNA3.1 SARS-CoV-2 S Epsilon	Addgene	#190461

(*Continued on next page*)

Continued		
REAGENT or RESOURCE	SOURCE	IDENTIFIER
pcDNA3.1-hACE2	Addgene	#145033
pcDNA3.1-TMPRSS2	Addgene	#190276
pscALPs LgBit	Addgene	#190277
pscALPs HiBit-FLuc	Addgene	#190278
Software and algorithms		
Study-specific analyses	GitHub (Broad Institute)	https://github.com/broadinstitute/sc2-cmu-study
LoFreq version 2.1.5	https://doi.org/10.1093/nar/gks918	https://csb5.github.io/lofreq/
viral-ngs 2.1.28	Broad Institute	dockstore.org/organizations/BroadInstitute/collections/pgs
Freyja v1.3.4	GitHub (Andersen Lab)	https://github.com/andersen-lab/Freyja
outbreaker2 (version 1.1.2)	https://doi.org/10.1186/s12859-018-2330-z	https://cran.r-project.org/web/packages/outbreaker2/index.html
Nextstrain	https://doi.org/10.1093/bioinformatics/bty407	github.com/nextstrain/ncov
MAFFT v7.471	https://doi.org/10.1093/bioinformatics/bty121	https://github.com/GSLBiotech/mafft
IQ-Tree	https://doi.org/10.1093/molbev/msu300	http://www.iqtree.org
TreeTime	https://doi.org/10.1093/ve/vex042	https://github.com/neherlab/treetime
baltic	Gytis Dudas	https://github.com/evogytis/baltic
TransPhylo	https://doi.org/10.1093/molbev/msw275	https://github.com/xavierdidelot/TransPhylo
Other		
4–20% Mini-PROTEAN TGX Precast Protein Gels, 15-well	BioRad	#4561096

RESOURCE AVAILABILITY

Lead contact

Further information and requests for resources and reagents should be directed to and will be fulfilled by the lead contact, Christopher Tomkins-Tinch (tomkinsc@broadinstitute.org).

Materials availability

This study did not generate new unique reagents.

Data and code availability

- All clinical viral genomic sequences were deposited in NCBI as part of BioProject GenBank: PRJNA715749 or GenBank: PRJNA622837. Wastewater environmental reads were deposited into the Sequence Read Archive (SRA). Accession numbers are listed in Data S1. Metadata associated with student groups are provided in Tables S1, S2, and S3 of Data S1. The single-subject, line-level data reported in this study cannot be deposited in a public repository because it is identifiable. However, summary statistics describing these data are described in the text, figures, and Data S1. Plasmids generated in this study have been deposited to Addgene: https://www.addgene.org/Jeremy_Luban/. Additional Supplemental Items are available at Mendeley Data: https://doi.org/10.17632/xsfy4p87pg.1.
- All original code has been deposited to GitHub and is publicly available as of the date of publication. DOIs are listed in the key resources table.
- Any additional information required to reanalyze the data reported in this paper is available from the lead contact upon request.

EXPERIMENTAL MODEL AND SUBJECT DETAILS

Ethics statement

The study was conducted at the Broad Institute with approval from the MIT Institutional Review Board under Protocol #1612793224 and from the WCG IRB under

Protocol #20210166: Viral Emergence and Spread in Community Settings. A templated Institutional Review Board protocol is provided in Methods S1.

Cell culture

Female human 293T/17 [HEK 293T/17] (ATCC CRL-11268) cells were obtained from and authenticated by the American Type Culture Collection (https://www.atcc.org). Cells were tested for mycoplasma contamination using the Mycoplasma Detection kit (Lonza LT07-318). Cells were cultured in humidified incubators with 5% CO_2 at 37°C in DMEM supplemented with 10% heat-inactivated FBS, 1 mM sodium pyruvate, 20 mM GlutaMAX, 1× MEM non-essential amino acids, and 25 mM HEPES, pH 7.2.

METHOD DETAILS

Wastewater collection and quantification

Installation and operation of wastewater samplers. Five on-campus sewage sites were monitored in Fall 2020, with six additional sites added in Spring 2021. The effluent collected at all sites originated from only on-campus sources. Of the five original sites, three were downstream of specific non-isolation dormitories, a fourth contained the wastewater from a dorm housing COVID-positive individuals in isolation, and a fifth was located at the confluence of two dormitories and the waste stream that began at the isolation dorm. The six sites added in Spring included four dormitory sampling locations (two of which were downstream of academic buildings), and two sites near academic buildings but upstream of residential buildings (Figure S3D).

Automatic wastewater samplers were custom built based off Reeves et al.[47] Each sample was a composite from a 24-h period. Samples were collected twice weekly in the fall, and three times weekly in the spring (Figure S3C). Automatic samplers pumped water continuously at a rate of approximately 4 gallons per 24 h. Samplers consisted of stainless-steel strainers deployed into the sanitary sewer. Silicone flexible tubing connected the strainer to a five-gallon high-density polyethylene (HDPE) jerrycan; water was displaced via a peristaltic pump run by a portable battery. After the 24-h sample collection period, the jerrycans were gently mixed and three 40 mL samples were collected for processing at each site. Samples were collected with sterile serological pipette tips in an autopipetter and transferred to sterile 45 mL conical tubes. Samples were stored on ice or placed in a 4°C refrigerator overnight for a maximum of 18 h prior to processing.

After each sampling event, the strainer and silicone tubing were cleaned by pumping a 10% bleach solution through the system. These components were dried between sampling events by storing them in locked wooden sampling boxes anchored above each open manhole. Each jerrycan was first sanitized with 10% bleach thrice, and was subsequently cleaned thrice with dish soap and water and allowed to dry. Upon deployment, wastewater was pumped through the system and sent back into the sewer prior to sample collection to rinse the strainer and tubing.

Quantification of viral concentration. In the Fall semester, all samples were sent to GT Molecular for viral titer quantification. At the beginning of the Spring semester, samples were processed both at GT Molecular and on campus, with CMU validating its data against results received from GT Molecular. From Feb. 15, 2021 onwards, samples were processed solely at CMU. During Spring 2021, technical duplicates were processed for three or four sites (of the eleven total) during each sampling event, to serve as an internal validation of viral concentration.

CMU followed a standard procedure to calculate viral titer.[48] Each sample volume was adjusted the next day to 40 mL and spiked with 13.6 μL Bovilis Coronavirus Calf Vaccine (BCoV) (Merck Animal Health Cat. No.16445), reconstituted in 2 mL 0.01% Tween 20 in 1× PBS. BCoV was added to determine viral recovery yield of the concentration step during subsequent RT-qPCR. The samples were inverted three times to mix. 400 μL of 5% Tween 20 was added to each tube and samples were inverted three times to mix. The samples were centrifuged at 7000 × g at 4°C for 10 min. The supernatant was carefully transferred to a fresh 50 mL conical tube without disturbing the pellet. The supernatant was concentrated with the InnovaPrep concentrating pipette. Elution was done in 0.075% Tween 20/25 mM Tris. The concentrated samples were stored on ice until all samples were processed.

Virus RNA was extracted with the QIAamp Viral RNA Mini Kit (Qiagen) with minor changes to the manufacturer's protocol. The tubes were incubated for 15 min at room temperature upon pipetting 140 μL of the concentrated wastewater tubes with 560 μL of AVL buffer containing carrier RNA. During the AW2 wash, the spin column was centrifuged three times, first for 3 min at full speed, and the next two spins for 1 min each at full speed and with open lids. For each spin the old collection tube was replaced with a new collection tube. After the third spin, the spin columns were placed in microfuge tubes and incubated with open lids for 15 min at room temperature to allow any remaining ethanol to evaporate. For the elution of RNA, 60 μL of nuclease-free water was added to the membrane, incubated at room temperature for 1.5 min, and spun at 6000 g for 2 min. The extracted RNA was stored on ice briefly until it was used for digital PCR.

The digital PCRs were performed as twoplex assays with TaqMan hydrolysis probes, on the QIAcutyOne 2plex (Qiagen) platform. QIAcuty One-Step Viral RT-PCR Kit (Qiagen) was used to quantify the viral load. The duplex reactions were 40 μL and contained 24 μL of the purified RNA, 1X One-Step Viral RT-PCR Master Mix, 1X Multiplex Reverse Transcription Mix, SARS-CoV-2 and BCoV forward and reverse primers at 0.4 μM and probes at 0.2 μM. SARS-CoV-2 nCOV_N1 RUO primers and probe and BCoV primers (NOC43-1 and NOC43-2) were purchased from IDT. The BCoV probe (NOC43-p) labeled with HEX fluorophore and OQA quencher was purchased from Sigma-Aldrich.

The QIAcuity was programmed to 50°C for 40 min for reverse transcription, 95°C for 2 min for initial heat inactivation, and 40 cycles of denaturation at 95°C for 5 s and annealing/extension at 55°C for 30 s.

Flow-mediated mass balance correction. Proximity to dormitories was prioritized for placement of wastewater samplers. In a few cases, there were other dormitories or academic buildings that contributed sewage upstream of specific dormitories (Figure S3D). In these cases, additional samplers were placed upstream, and the background SARS-CoV-2 concentration for samples from upstream sites was subtracted from concentrations obtained from downstream sites, using a flow-mediated mass balance based on building-level portable water consumption to account for dilution.

Viral genomic sequencing

Members of the CMU community underwent diagnostic testing for SARS-CoV-2 infection using either saliva or nasal specimens, collected in response to random surveillance testing and reflexive testing. Residual material was only available for saliva

specimens, accounting for a fraction of known cases during the 2020–2021 school year (Figures 2A and S2B).

Saliva samples were collected from members of the campus community and sent to Warrior Diagnostics, Inc., for clinical diagnostic RT-qPCR testing. Excess material from specimens found to be positive for SARS-CoV-2 was inactivated and sent to the Broad Institute of MIT and Harvard for viral genomic sequencing. In initial sequencing rounds, samples were treated with 5 uL of proteinase K; we determined that excluding this step did not negatively impact sequencing quality, and did not include it in later sequencing. Total RNA was extracted from the samples using the Thermo Fisher MagMAX Viral RNA Isolation kit. Concentration of viral RNA was determined through RT-qPCR with primers and probes targeting the SARS-CoV-2 N gene. Illumina sequencing libraries were prepared from tiled amplicons amplified using the ARTIC v3 primer set.[49–51] The libraries were pooled and sequenced on Illumina NovaSeq and NextSeq instruments.

During the 6 epi-weeks from Sunday, Feb. 9 through Mar. 20, 2021, viral RNA from aliquots of 42 samples of excess extracted wastewater was sequenced via the same ARTIC v3 procedure. Samples were sequenced in three batches. The final batch of nine samples was sequenced with technical replicates obtained by splitting the cDNA produced from the RNA template prior to library construction.

Functional characterization of spike glycoprotein mutations

Lentivirus production. 24 h prior to transfection, 6×10^5 HEK-293T cells were plated per well in 6-well plates. All transfections used 2.49 μg plasmid DNA with 6.25 μL TransIT LT1 transfection reagent (Mirus, Madison, WI) in 250 μL Opti-MEM (Gibco). Single-cycle HIV-1 vectors pseudotyped with the indicated SARS-CoV-2 Spike constructs were produced by transfection of HIV-1 pNL4-3ΔenvΔvpr luciferase reporter plasmid (pNL4-3.Luc.R-E−; NIH AIDS Reagent Program, Division of AIDS, NIAID, NIH: from Dr. Nathaniel Landau; ARP Cat #3418) with the indicated Spike expression plasmid, at a ratio of 4:1.

Lentivirus infectivity assays. 16 h prior to transduction, HEK-293T cells stably expressing ACE2/TMPRRS2 as previously described[52] were plated at 3×10^4 per well. Cells were incubated in virus-containing media for 16 h at 37°C after which fresh media was added to cells. 48 h after transduction, cells were assessed for luciferase activity using the Promega Steady-Glo system (Promega Madison, WI).

Western blot analysis. Tissue culture media and cell lysate were collected 60 h after transfection to produce lentivectors. Supernatant containing Spike pseudotyped particles was layered on a 20% sucrose cushion in PBS and spun at 110,000 × g at 4°C for 2 h. The pellet was washed once with ice-cold PBS and resuspended in 15 uL of 2× SDS gel loading buffer. After removal of supernatant, transfected cells were lysed in 300 uL 2× SDS-PAGE loading buffer. Protein preps were boiled for 5 min and then separated by SDS-PAGE on a 10–20% Tris-Gycine gel (BioRad). Proteins were electro-transferred from gels to nitrocellulose membranes, which were blocked for an hour with Licor Blocking Buffer and detected with the indicated antibodies.

Cell fusion assay. ACE2/TMPRSS2 expressing cells were prepared by transfecting 293T cells with pcDNA3.1- ACE2 and pcDNA3.1-TMPRSS2 along with pscALPs LgBit. Spike expressing cells were prepared by transfecting 293T cells with pcDNA3.1- constructs expressing the specified codon optimized SARS-CoV-2 Spike proteins, in addition to the pscALPs HiBit-FLuc fusion expression vector. 24 h after transfection,

ACE2/TMPRSS2 and Spike expressing cells were lifted from plates with TrypLE and plated together in a 1:1 ratio for a total of 40,000 cells in 96-well white-walled tissue culture plates. Promega Endurazine substrate was added to cells according to the manufacturer's protocol 1 h after plating and fusion was analyzed 4 h later. Fusion signal of HiBit-LgBit interaction was normalized to Fluc signal to control for transfection efficiency. Background fusion was determined by using 293T cells transfected with pscALPs HiBit-Fluc alone with control pcDNA3.1- plasmid without Spike.

QUANTIFICATION AND STATISTICAL ANALYSIS

Epidemiological modeling

Relative risk analysis. For each risk factor of interest (Figure S1C), we calculated the relative risk and its confidence interval. The relative risk is defined as:

$$\frac{\left(\frac{\text{cases with risk factor}}{\text{students with risk factor}}\right)}{\left(\frac{\text{cases without risk factor}}{\text{students without risk factor}}\right)}.$$

The natural logarithm of relative risk is approximately normally distributed with squared standard error defined as:

$$\frac{\frac{\text{students with risk factor} - \text{cases with risk factor}}{\text{cases with risk factor}}}{\text{students with risk factor}} + \frac{\frac{\text{students without risk factor} - \text{cases without risk factor}}{\text{cases without risk factor}}}{\text{students without risk factor}},$$

enabling calculation of 95% confidence intervals.[53]

Each risk factor was studied individually; we could not assess the relationship between factors (*e.g.*, whether the increased risk for males is explained by the increased risk for athletes) as we do not have information on the number of individuals at the intersection of risk factors.

Chi-squared analysis. For categorical variables with multiple levels (*i.e.*, sports teams, sports contact levels, sports locations, residence halls, and class years), we assessed whether SARS-CoV-2 cases were distributed uniformly across levels. Specifically, we conducted chi-square goodness-of-fit tests where the expected number of cases in level i was:

$$expected_i = sum_j(\text{cases in category } j) * \frac{\text{students in category } i}{sum_j(\text{students in category } j)}$$

P-values were calculated using the chi-square distribution, with degrees of freedom equal to one less than the number of levels (Data S1).

The association between two quantitative variables (*i.e.*, sports team testing rates and incidence rates) was assessed using Pearson's correlation.

Regression model. We constructed a linear model of COVID-19 incidence rates (*i.e.*, case counts/number of residents) in a residence hall as a function of: number of students, percent occupied (number of available beds/number of students), number of floors, the presence of a meal plan requirement (''dining hall''), the presence of an in-unit bathroom (''private bath''), the number of resident advisors (RAs), square footage, ceiling height, and volume per person (*i.e.*, a proxy for air volume: floor area * ceiling height/number of residents). We assessed for multicollinearity among our predictive variables and identified many correlations (Figure S4A). Because of these relationships, our coefficients and their confidence intervals may not be robust; however, the model's predictive power and R^2 remain unaffected.

We evaluated 511 models, using all possible combinations of predictors (2^9–1 combinations). For each model, we calculated the AIC and the BIC and selected the model with the lowest AIC (−41.95) and BIC (−40.98). The model, with an adjusted R^2 of 0.95 (Appendix Figure 4B) is as follows:

$$incidence_i \; = \; \beta_1 * percent\_occupancy_i + \beta_2 * private\_bathroom_i$$

$$\beta_1 \; = \; 0.0015; \; \beta_2 \; = \; 0.0587$$

We evaluated the model via examination of the residual plot for heteroscedasticity (Figure S4C) and via leave-one-out cross-validation, *i.e.*, we fit the model using (*N*-1) data points and calculated the residual for the remaining data point to determine the root mean squared error (Figures S4D and S4E).

Time series data. We used 2019 US Census Bureau data to determine the population sizes of Mesa County and Colorado. Using the United States county-level COVID-19 data, we determined a per-day incidence of COVID-19 cases and deaths for Mesa County and for all of Colorado. We also determined a per-day incidence rate for COVID-19 cases at CMU. We plotted rolling sums of 7 daily COVID-19 incidence rates to determine weekly incidence rates (Figures 2C and S1A). We calculated Pearson correlation coefficients between CMU's and Mesa County's weekly incidence rates using the numpy.corrcoef function.

We determined test positivity rates per semester by dividing the total number of cases by the total number of tests.

Contact tracing analyses. A positive individual's close contacts were defined as individuals who were within 6 feet of each other for 15 min or more, in the 48-h time period prior to symptom onset or test date, regardless of whether masks were worn. Each individual who tested positive was asked to report their close contacts. This information was collated to include the number of contacts they reported, and an anonymized identifier for reported contacts who tested positive at any point throughout the academic year. We determined the number of individuals who tested positive within 7 days of being reported as a close contact of a positive individual.

For all reported pairs of two positive individuals where one was identified as a close contact of the other and where both had sequenced viral samples, we calculated the genomic distance (*i.e.*, the number of single-nucleotide mutations differing between their consensus-level viral genomes, Figure S2A).

WiFi analyses

Data acquisition and cleaning. Our partners at Degree Analytics, a behavioral analytics company, collected WiFi connectivity data for Colorado Mesa University from August 2020 through May 2021. Degree Analytics collected the date, starting time, and duration of a specific device's connectivity to WiFi access points (APs) that were distributed across all university-affiliated spaces, including academic buildings, residence halls, dining locations, administrative buildings, and athletic spaces. Degree Analytics then ran device-specific data through a proprietary algorithm to produce pairwise interactions, defined when two individuals simultaneously access the same (one or more) APs for at least 15 min, and unpaired connections, where one individual connects to (one or more) APs for at least 15 min. The proprietary algorithm accounts for some level of uncertainty; *e.g.*, if a device were to disconnect because it lost power and re-connect to the same AP a minute

later, the device would be considered present at that AP before, during, and after the brief lapse.

Importantly, we cannot confirm if two individuals were within a reasonable range of each other for COVID-19 transmission; most AP-based interactions imply that the devices are within 10 m of one another (but in the extreme cases, as much as 30 m apart), which can span a wall or a floor. However, we anticipate that interactions at a distance that could lead to COVID-19 transmission are present in our pairwise data. Additionally, social networks, regardless of transmission mitigation strategies (*e.g.*, mask-wearing), can be inferred from these data.

The data was provided in the format of a device identifier (subsequently cross-referenced with metadata, including COVID-19 testing results of the user), the date, the AP (or set of APs), the building location (*e.g.*, "Hall B" or "academic building"), the length of time the user connected to the AP, and the (possibly empty) list of other devices that were simultaneously connected to the same AP, each with the duration of overlap.

To analyze pairwise interactions, the data were de-duplicated and cleaned, as instructed by Degree Analytics. We limited users within the dataset to only WiFi-authenticated students, thus removing guests, faculty/staff, and stagnant devices on the network. Next, we removed student identifiers that were only ever present 3 or fewer times on campus over the entire year, as we assessed that these individuals were remote students who infrequently commuted to and participated in the on-campus CMU community or testing program. This cleaning removed 45,354 identifiers from the original 53,100 identifiers, producing a finalized dataset of 7,746 students.

To assess for differences in presence across buildings and semesters, we quantified the daily number of AP connections and the median duration of AP connections per building and per day of week, for each semester (Data S1; Figure S6). Due to the differences in connectivity patterns across semesters, we conducted all analyses on a per-semester and per-day basis.

Interaction metric comparisons. We examined the daily interaction patterns for users (*i.e.*, nodes in our network), dividing them into students who tested positive at some point over the semester ("positives") and students who did not test positive over the entire semester ("negatives"). Within our network, edges represent pairwise interactions. We quantified an individual student's daily interactions via: (1) number of unique contacts, (2) average exposure time per contact, and (3) number of days on campus (Data S1; Figures 3A, S5A, S5B, and S7B). We also determined the proportion of users (positive or negative) on campus each day, calculated the Pearson correlation between the positive and negative proportions, and compared these proportions via the Mann Whitney U test (Figures S5C and S5D).

To investigate WiFi-derived contacts during the isolation period, we calculated the median number of unique contacts across all positive individuals for each day in the 10 days prior to vs. after an individual's positive test (Figure S7A). The average of the daily medians for each 10-day period was then used to calculate the percent change between the two periods.

Next, we redefined positive users as those within the 10-day window before a positive test ("pre-positives"), and negative users as those who were not

currently within a 10-day window prior to testing positive (regardless of testing status before or after the 10-day window). We examined pairwise interaction patterns for pairs of users with: (1) two pre-positive users who were reported as contact tracing pairs (*i.e.*, CC positive pairs), (2) two pre-positive users who were not listed as pairs in contact tracing (*i.e.*, non-CC positive pairs) (3) a pre-positive and a negative user (mixed pairs), and (4) two negative users (negative pairs). We quantified daily pairwise interactions via the median and the total daily interaction duration and assessed for differences for both the Fall and Spring semesters (Data S1; Figures S7C and S7D).

For all comparisons, we used the Mann Whitney U test to produce uncorrected p values.

Attribute assortativity. The attribute assortativity (AA) coefficient is a metric that quantifies the tendency for users to interact within vs. across particular cohorts.[54] To compute this metric, we compared interactions either between positive and negative individuals, or pre-positive and negative individuals (as defined in the previous sections). The AA coefficient is bounded between −1 and 1, where −1 represents a network where individuals only interact across-group, 0 represents a perfectly mixed network, and 1 represents a network where individuals only interact within-group (Figure 3B).

We calculated the AA coefficient for sub-groups of individuals per day (using the NetworkX Python package),[55] and generated 95% confidence intervals (CI) by permuting attribute labels (40 times, with the lowest and second highest AA defining the bounds of the 95% CI) across individuals within each day's network (Figures 3C and S8A–S8C). We expect CIs to overlap with the per-day AAs for approximately 95% of the days if positive individuals were equally likely to interact with other positive individuals as they were with negative individuals. We ran this procedure three times for each semester (six times in total):

1. Defining positives as individuals who test positive for COVID-19 at some point during the semester (Figures S8A and S8B)
2. Defining positives as all individuals who are within 10 days of testing positive (*i.e.*, "pre-positives"; Figures 3C and S8C)
3. Defining pre-positives as individuals who are within 10 days of testing positive and are not listed in contact tracing data as having a pairwise association with another positive individual (*i.e.*, "non-CC pre-positives"; Figures S9A and S9B)

For the next analyses, we used the definition of pre-positives defined above as (2).

We assessed the relationship between pre-positive vs. negative attribute assortativity coefficients and case counts, with the hypothesis that social network structure may be predictive of future case counts. We plotted both raw and smoothed data (via the Savitzky-Golay filter; window length = 17, polynomial order = 4).[56] We determined the lag time, in days, that produced the maximal Pearson correlation between daily AA and case counts, for both the raw and smoothed data and for both the fall and spring semesters (Figures 3D, 3E and S8D–S8F).

WiFi analyses specific to the B.1.429.1 lineage. We compared the total exposure time and the median interaction duration (Data S1, Figures S12A and S12B) for pairs of B.1.429.1-positive cases, pairs of non-B.1.429.1-positive cases, and pairs of negative individuals.

We constructed a subgraph of the network where nodes represent individuals in the pre-positive 10-day window, and edges connect two pre-positive individuals with documented proximity. We defined two individuals as within the same social network if they could be connected by a path. We examined whether the distribution of the viral genome SNV distances for B.1.429.1 pairs within the same social network differed from the distribution of SNV distances for B.1.429.1 pairs in different social networks (Figure 6D) using the Mann Whitney U test.

To test the hypothesis that B.1.429.1 individuals clustered together in the network, we quantified the shortest path[57] between pairs of individuals, where: 1) both individuals had the B.1.429.1-lineage virus, or 2) one individual had B.1.429.1 and one had a non-B.1.429.1 virus, using the Mann Whitney U test.

Wastewater analyses

Comparison of viral titers and weekly case counts. We conducted two analyses to assess the relationship between viral titer and weekly case count. We first compared each individual wastewater sample against its corresponding weekly case count, across all collection sites except for Site 5 which collected effluent from isolated positive cases, using .Spearman's correlation coefficient.

Second, we calculated each hall's average wastewater viral titer (*i.e.*, the average of available samples from Sunday through Saturday) and each hall's total case count for each week. If a hall had no wastewater samples collected in a given week, it was removed. We then proceeded in a hall-wise fashion to determine the sign of the slope of the viral titer and case count (*i.e.*, to assess whether titer and case count rose or fell together from one week to the next). We created a contingency table of the sign of the slope of viral titer vs. the sign of the slope of case counts, and evaluated its significance via Fisher's exact test.

Viral sequencing analysis. We inspected sequence data for the presence of regional blindspots in the genome distinct to wastewater as a sample type. To assess whether specific regions of the genome were more susceptible to degradation in wastewater vs. in clinical samples, we normalized read depth per base for each sample and plotted the distribution of depth across all wastewater samples, alongside a corresponding plot of depth from all clinical samples (Figures S10B and S10C). We compared the median normalized depth per amplicon between wastewater and clinical samples by calculating the Pearson correlation (Figure S10E). Next, we compared amplicon read depth and Shannon entropy within the primer regions of wastewater sequences by calculating the Pearson correlation (Figure S10D). We used entropy data from a CDC-curated Nextstrain analysis focused on data from Colorado as of August 2021.[58]

Development of quality controls for identifying SNVs in wastewater. We evaluated three quality control filters to remove spurious SNVs identified in wastewater: minimum allele frequency (AF), minimum read depth (DP), and presence in each of two replicates from the same cDNA source (Reps) (Figure 5E). For both AF and DP, we independently toggled their threshold from the absolute minimum (AF = 0, DP = 0) to the absolute maximum (AF = 1, DP = 29903). Since replicates were only available for nine of the forty-two wastewater samples, analyses were limited to those nine samples.

We investigated which quality control mechanisms identified the greatest number of wastewater SNVs present in any Colorado clinical sample. For AF and DP thresholds, sensitivity and specificity were defined as follows:

WW = set of SNVs in wastewater samples
CO = set of SNVs in Colorado clinical samples

$$\textit{Sensitivity of AF threshold } x = \frac{cardinality(\ WW \cap CO \cap \{SNVs\ with\ AF \geq x\})}{cardinality(WW \cap CO)}$$

$$\textit{Sensitivity of DP threshold } x = \frac{cardinality(\ WW \cap CO \cap \{SNVs\ with\ DP \geq x\})}{cardinality(WW \cap CO)}$$

$$\textit{Specificity of AF threshold } x = \frac{cardinality((WW - (WW \cap CO)) \cap \{SNVs\ with\ AF < x\})}{cardinality((WW - (WW \cap CO)))}$$

$$\textit{Specificity of AF threshold } x = \frac{cardinality((WW - (WW \cap CO)) \cap \{SNVs\ with\ AF < x\})}{cardinality((WW - (WW \cap CO)))}$$

For the Reps filter, sensitivity and specificity were calculated for each of the nine samples, rather than for the entire subset of samples. Sensitivity and specificity were defined as follows:

$WW_{X,union}$ = set of SNVs found in either replicate of sample X.

$WW_{X,intersection}$ = set of SNVs found in both replicates of sample X

$$\textit{Sensitivity for sample } X = \frac{cardinality(\ WW_{X,intersection} \cap CO\ )}{cardinality(\ WW_{X,union} \cap CO\ )}$$

$$\textit{Specificity for sample } X = \frac{cardinality(\ WW_{X,union} - WW_{X,intersection} - ((\ WW_{X,union} - WW_{X,intersection}) \cap CO))}{cardinality(\ WW_{X,union} - (WW_{X,union} \cap CO))}$$

Analysis of the expected number of unique SNVs contributed by additional samples. We estimated the number of unique SNVs present within a given number of CMU wastewater or clinical samples (Figures S11B and S11C). For clinical samples, we bootstrapped 100 times over each possible subset size (*i.e.*, from 1 sample to all samples) to curate a set of clinical samples. We calculated the total number of unique consensus-level SNVs across each set of *n* samples, then found the average number of unique consensus-level SNVs that we could expect *n* samples to contribute.

For wastewater samples, we also bootstrapped 100 times over each possible subset size (*i.e.*, from 1 sample to all 42 samples) to curate a unique set of wastewater samples. We then calculated 1) the average number of SNVs across sets of *n* samples, and 2) the average number of SNVs of AF greater than or equal to 25% across sets of *n* samples. We repeated this process with the wastewater samples with technical replicates, again bootstrapping 100 times over each possible subset size (i.e. from 1 sample to all 9 samples) to calculate the average number of replicate-confirmed SNVs that we could expect from a set of *n* samples.

Finally, we calculated the smoothed first derivative (*i.e.*, change in SNV count as a function of the number of samples) using a window size of 5 (Figures S11D and S11E).

Lineage identification in wastewater. To detect lineages across our wastewater samples, we called SNVs using LoFreq with default parameters. We estimated the relative abundance of constituent lineages using Freyja v1.3.4.[39] We limited analyses to samples with at least 30% genome coverage, and lineages that were detected with 95% confidence (per Freyja's built-in bootstrapping capabilities, 5000 replicates) with at least 3% abundance. Lineages were assigned using Freyja with a global UShER tree downloaded on March 14, 2022.

Viral genomic analyses

Viral genome assembly. Using the viral-ngs v2.1.28.0 pipeline, reads from sequenced pools were demultiplexed, filtered to remove adapter and contaminant sequences, depleted of reads mapping to the human genome, and assembled by alignment to the reference sequence NC_045512.2. A total of 184 samples (of 278 received) from clinical diagnostic tests were successfully sequenced to yield viral genomes with median assembly length of 29,827 bases (Figure S2C). Assembled viral genomes with at least 24000 unambiguous bases were deposited in NCBI GenBank as part of BioProjects GenBank: PRJNA715749 or GenBank: PRJNA622837; accession numbers are listed in Data S1.

Lineage assignment. Lineages were assigned to viral genomes using Pango v4.0.6 with pango-data v1.9.[34,59]

Phylogenetic analysis. CMU genomes were aligned to the reference sequence NC_045512.2 with MAFFT v7.471 using the "–addfragments" and "–keeplength" arguments. These parameters, widely used in SARS-CoV-2 genome alignment, can produce alignments which omit insertions. This limitation was deemed acceptable due to the rarity of known insertions in the SARS-CoV-2 genome at the time of sampling.

Using the Nextstrain augur pipeline, a maximum likelihood (ML) tree was created via IQ-Tree using a GTR mutation model, as well as a time-resolved tree via TreeTime, both rooted to the ancestral reference genome, NC_045512.2.[60–62] A filter was specified for TreeTime to exclude outlier sequences >4 interquartile distances from the root-to-tip vs. time (*i.e.*, molecular clock mean mutation rate) regression. Internal tree nodes were assigned their marginally most likely dates. CMU samples were placed in the context of viral genomes from state, national, and global datasets, weighted toward those collected in the US Mountain West or of short genetic distance from CMU viral genomes. The contextual genomes were obtained as part of the open dataset of pre-aligned sequences curated by Nextstrain.[63] Default augur quality thresholds were applied to input sequences.

To identify introductions to the campus community, ancestral state reconstruction was performed using TreeTime to produce a binary value indicating whether each viral genome or internal ancestral tree node was university-associated or not. A state change from not-associated to university-associated descendant cases was considered a putative introduction. Sub-trees for each introduction event were extracted and plotted using the *baltic* Python library,[64] for those where the confidence of the inferred state was >0.8.

The number of intermediate hosts in a cluster noted to span semesters was estimated using Trans-Phylo, and generation time distribution parameters reported previously (shape = 3.63, scale = 1.408).[36]

Overdispersion analyses. The distribution of offspring per cluster was calculated as the total number of individuals in the cluster minus one (*i.e.*, the introduction case itself). A negative binomial distribution was fit to the data using '*fitdistplus*' in R 4.1.2.

The manual contact tracing identified individuals in contact with an index case for more than 15 min at less than 6 feet within the prior 48 h of the earliest of their positive test date or their symptom onset date. A negative binomial distribution was fit to the number of contacts reported per positive case.

The number of contacts of each positive user, as inferred from the WiFi proximity dataset in the 48 h prior to the earlier date of either positive test or symptom onset, was quantified, and a negative binomial distribution was fit to the resulting data.

Transmission network reconstruction. To reconstruct transmission networks, we first excluded sequences with >7% ambiguous bases. The remaining sequences were aligned to the reference genome NC_045512.2 using MAFFT v7.471 with the parameters "–addfragments" and "–keeplength". Positions identified as prone to sequencing error or homoplasy were masked with ambiguous bases using the positions previously documented.[65] The 5′ AND-3′ untranslated regions of the genome were also masked over the reference sequence positions 1–265 and 29558–29903. Any sequences with >10% ambiguity or >7% gaps across the genome were excluded.

Three forms of contact data were included in the transmission network model: 1) contact tracing data from university tracing efforts; and contacts assumed from shared proximity to Wi-Fi access points for individuals in contact within 2) 2 days or 3) 10 days of both case dates. We also developed models with 4) solely genomic and 5) solely contact tracing data for comparison. Case dates were the earlier of the date of symptom onset, when known, and the date of diagnostic test.

The probability of direct transmission between cases bearing B.1.429.1-lineage virus was estimated from case dates, viral genomes, and contact data using outbreaker2 with parameters previously described[66] and a single chain of 40,000 iterations (of which the first 10% were discarded). Visualizations include all transmission events with a probability greater than or equal to 25%. For the three networks generated with a combination of genomic and contact data, we compared clusters of 2 or more individuals via the Jaccard distance.

Med, Volume *3*

Supplemental information

Multimodal surveillance of SARS-CoV-2 at a university enables development of a robust outbreak response framework

Brittany A. Petros, Jillian S. Paull, Christopher H. Tomkins-Tinch, Bryn C. Loftness, Katherine C. DeRuff, Parvathy Nair, Gabrielle L. Gionet, Aaron Benz, Taylor Brock-Fisher, Michael Hughes, Leonid Yurkovetskiy, Shandukani Mulaudzi, Emma Leenerman, Thomas Nyalile, Gage K. Moreno, Ivan Specht, Kian Sani, Gordon Adams, Simone V. Babet, Emily Baron, Jesse T. Blank, Chloe Boehm, Yolanda Botti-Lodovico, Jeremy Brown, Adam R. Buisker, Timothy Burcham, Lily Chylek, Paul Cronan, Ann Dauphin, Valentine Desreumaux, Megan Doss, Belinda Flynn, Adrianne Gladden-Young, Olivia Glennon, Hunter D. Harmon, Thomas V. Hook, Anton Kary, Clay King, Christine Loreth, Libby Marrs, Kyle J. McQuade, Thorsen T. Milton, Jada M. Mulford, Kyle Oba, Leah Pearlman, Mark Schifferli, Madelyn J. Schmidt, Grace M. Tandus, Andy Tyler, Megan E. Vodzak, Kelly Krohn Bevill, Andres Colubri, Bronwyn L. MacInnis, A. Zeynep Ozsoy, Eric Parrie, Kari Sholtes, Katherine J. Siddle, Ben Fry, Jeremy Luban, Daniel J. Park, John Marshall, Amy Bronson, Stephen F. Schaffner, and Pardis C. Sabeti

Supplemental Items

Figure S1. Incidence rates at Colorado Mesa University (CMU), with surrounding areas for context, and epidemiological risk factors for SARS-CoV-2 positivity at CMU. Related to Figure 2.

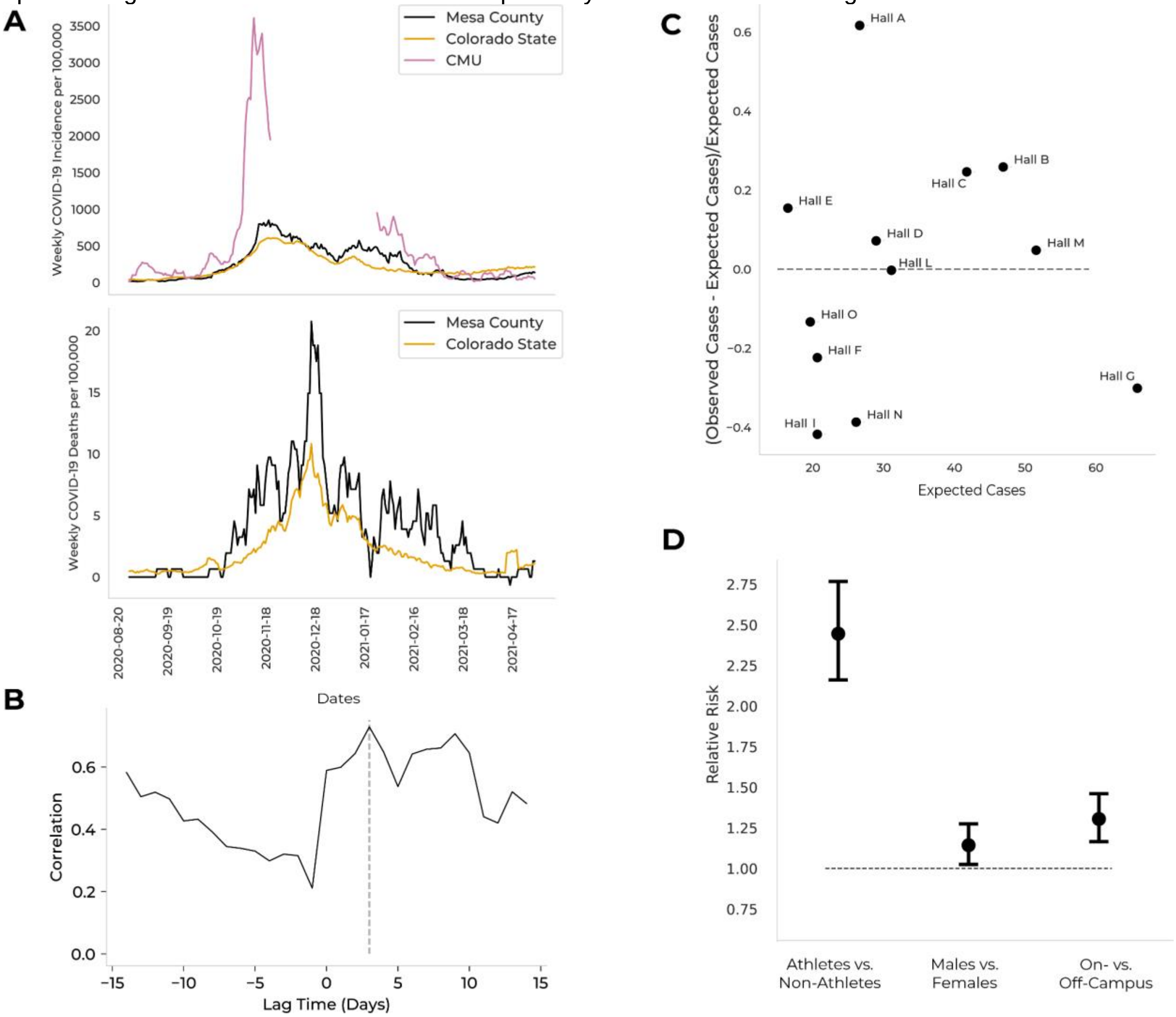

A. Weekly COVID-19 incidence at Colorado Mesa University (CMU; pink), in Mesa County (black), and in Colorado (orange) over the 2020–2021 academic year (upper). Weekly COVID-19 death rate in Mesa County (black) and in Colorado (orange) over the 2020–2021 academic year (lower). Data from CMU were not analyzed from November 21–January 18 due to winter recess. The correlations between CMU incidence rates and Mesa County incidence rates (correlation = 0.73, lag = 3 days) and Mesa County death rates (correlation = 0.48, lag = 8 days) were tested with lag times between -14 days (0 days for deaths) and 14 days, and the maximum correlations and corresponding lag times are reported. **B.** The correlation between CMU incidence rates and Mesa County incidence rates *vs.* the lag time. The dashed line at $y = 3$ indicates the lag time that maximizes the cross-correlation. **C.** The difference between the number of cases observed and the number of cases expected (given residence hall population size and scaled by number of cases expected) *vs.* the number of cases expected, per residence hall. The dashed line at $y = 0$ separates halls with more (above) or fewer (below) cases observed than expected. **D.** Relative risk (RR) of testing positive for COVID-19 given sports team membership (athletes *vs.* non-athletes), sex (males *vs.* females), or residential status (on- *vs.* off-campus). Circles represent the relative risk, with whiskers extending to the upper and lower bounds of the 95% confidence interval. The dashed line at RR = 1 represents the null

hypothesis (*i.e.*, that there is no association between the risk factor and test positivity). An RR greater than 1 implies that the first group listed (*i.e.*, athletes, males, or on-campus students) had a greater risk of testing positive than the second group listed (*i.e.*, non-athletes, females, or off-campus students). Athletes were tested 1.55 times as often as non-athletes and on-campus students were tested 1.80 times as often as off-campus students; thus, sports participation was associated with increased risk of SARS-CoV-2 positivity, while residential living was not.

<u>Figure S2.</u> Detection of outbreak groups using viral genomic sequencing of cases. Related to Figure 4.

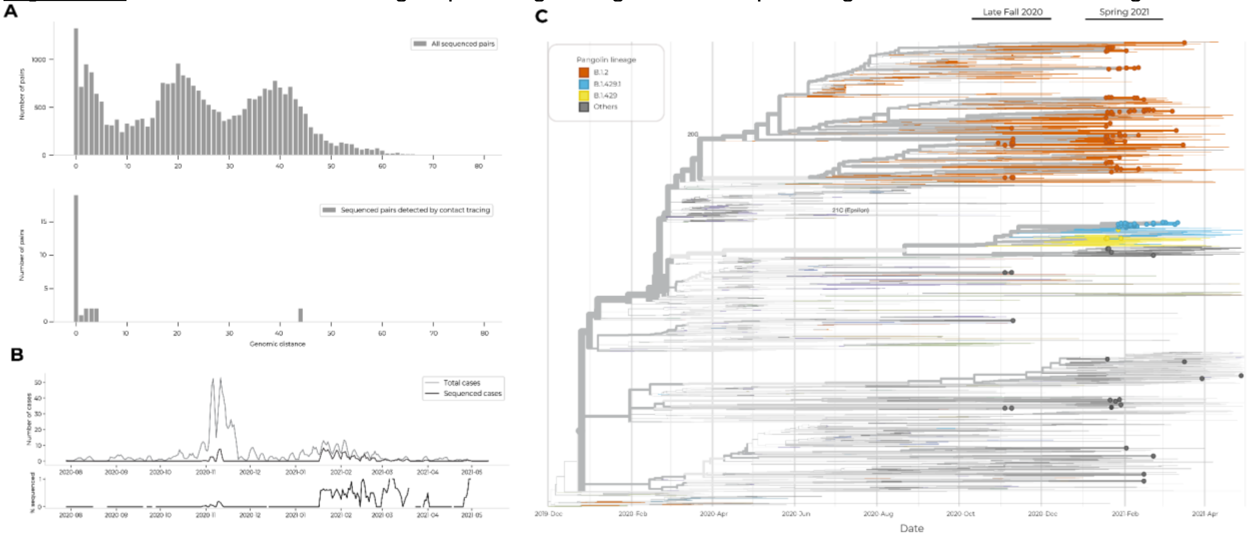

A. Distributions of genetic distance for all pairs of viral genomes (upper) and for pairs of viral genomes from positive individuals sharing interactions reported during contact tracing (lower). Two outlier pairs (lower) had a genetic distance of 44 SNVs. In each of these pairs, an individual testing positive at the end of January 2021 had virus of the B.1.2 lineage and indicated close contact with an individual who tested positive in early February (5-6 days later) with virus of the B.1.429.1 lineage. **B.** Sequencing of positive cases via genomic surveillance across both semesters. Sequencing began October 2020, capturing cases associated with a Halloween-related outbreak. Shown are the total number of cases (top, thin line), total number of sequenced cases (top, bolded line), and percent of positive cases that were sequenced (bottom). **C.** Phylogenetic placement and temporal sampling of viral genomes sequenced from CMU clinical diagnostic tests. CMU nodes are depicted as dots among a global tree of contextual sequences, weighted toward sequences collected from Colorado and surrounding states and genomes close in genetic distance to CMU sequences. Interactive version available at https://auspice.broadinstitute.org.

Figure S3. Summary of wastewater surveillance implemented at Colorado Mesa University. Related to Figure 5.

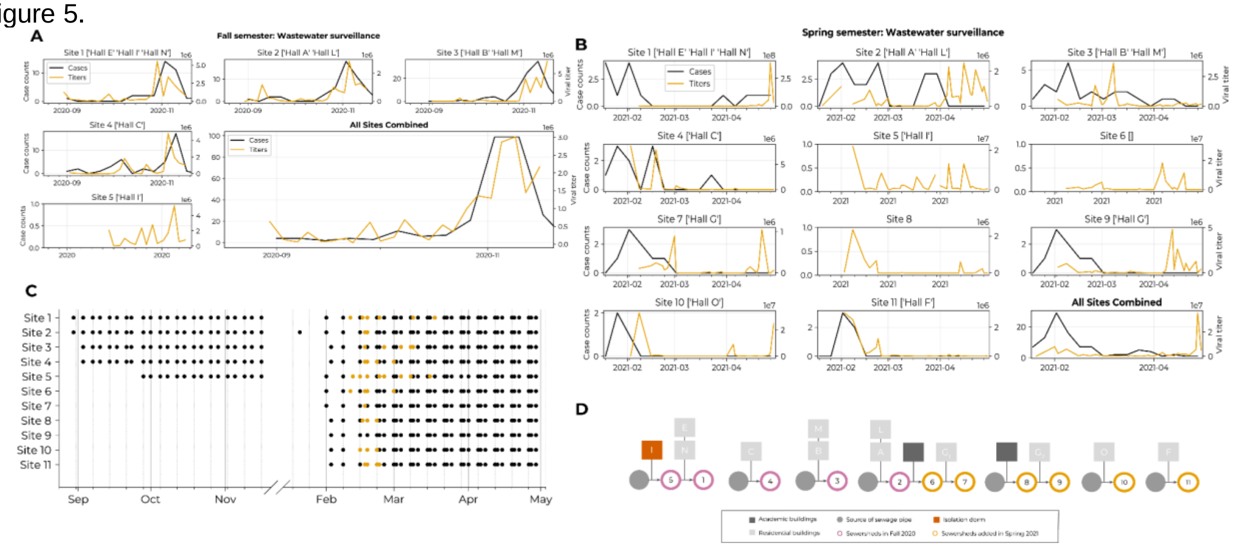

A-B. Comparison of wastewater viral titer (orange) and weekly case count (black) for each wastewater site in the Fall semester (**A**) and Spring semester (**B**). **C.** Sample collection frequency for wastewater surveillance in the 2020–2021 academic year. Wastewater samples were collected twice weekly in the Fall across 5 sites, and three times weekly in the Spring across 11 sites. Each dot represents an independent collection event, with dots shown in orange representing sequenced wastewater samples. **D.** Schematic showing the residential or academic buildings (squares) that contributed effluent to each monitored wastewater site (circles). Residence hall I (red square) housed a small number of students who had tested positive and were in isolation. Wastewater sites are represented by circles, with filled-in gray circles representing sites used to collect baseline measurements (*i.e.*, with no upstream contributors to the particular sewage system). Sites shown in pink were added in Fall 2020 and remained in use into Spring 2021, while sites shown in orange were added in Spring 2021.

Figure S4. Predictors of COVID-19 incidence rates in Colorado Mesa University residence halls *via* a linear regression model. Related to Figure 2.

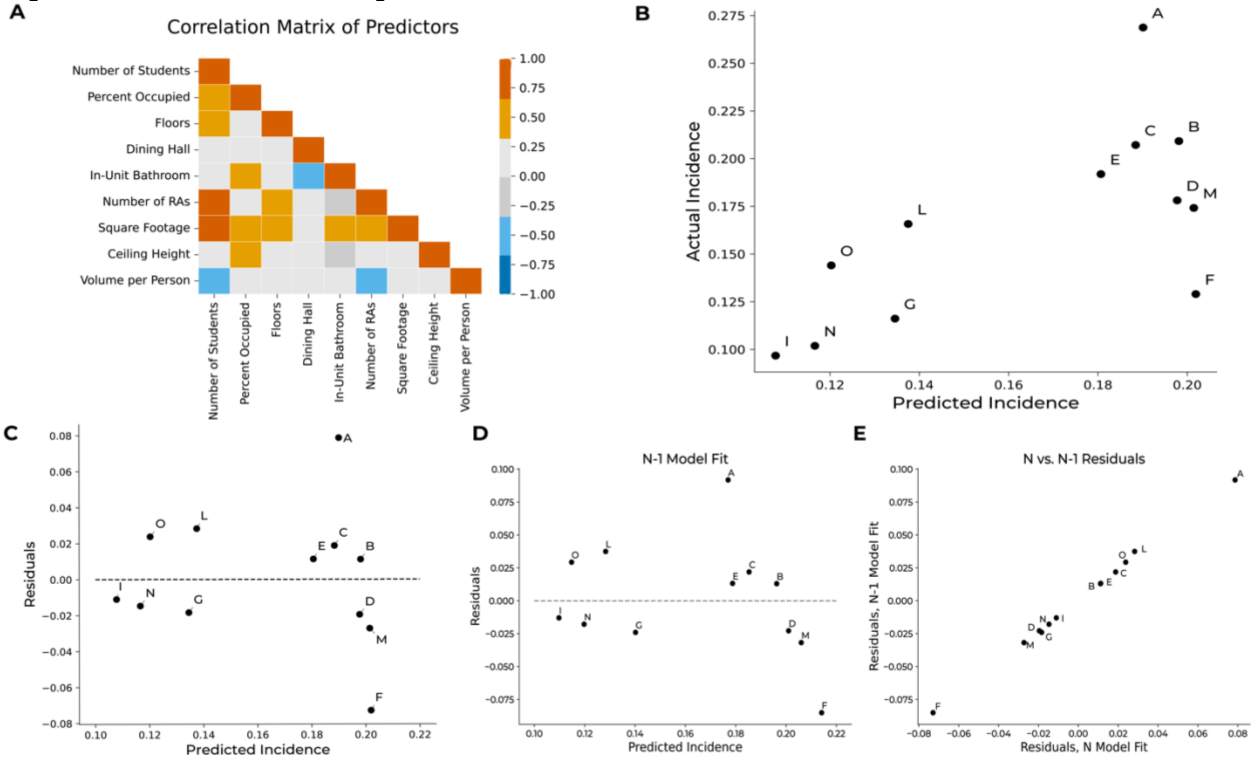

A. Descriptive attributes of residence halls, including number of students, percent occupied (number of available beds / number of students), number of floors, the presence of a meal plan requirement (“dining hall”), the presence of an in-unit bathroom (“private bath”), the number of resident advisors (RAs), square footage, ceiling height, and volume per person, were used as predictors in the regression model. Non-zero correlation coefficients between pairs of predictive variables indicate multicollinearity. **B.** Observed *vs.* predicted incidence rates, by residence hall. Predictions come from the model that minimized the Akaike and Bayesian Information Criteria. **C.** Residuals *vs.* predicted incidence rates, by residence hall. Root-mean-square-error = 3.6%. Halls A and F contribute to heteroscedasticity. **D.** Residuals *vs.* predicted incidence rates, from leave-one-out cross-validation, by residence hall. Leave-one-out cross-validation (*i.e.*, *N*-1 model fit, where *N* = number of residence halls) was conducted such that each hall’s incidence rate was predicted *via* a linear model whose coefficients were determined with training data from all other halls. Root-mean-square-error = 4.2%. **E.** Residuals from the leave-one-out cross-validation (*N*-1 model fit) *vs.* residuals from the full model (*N* model fit).

Figure S5. On-campus presence, as inferred by WiFi proximity data, by semester and by user category. Related to Figure 3.

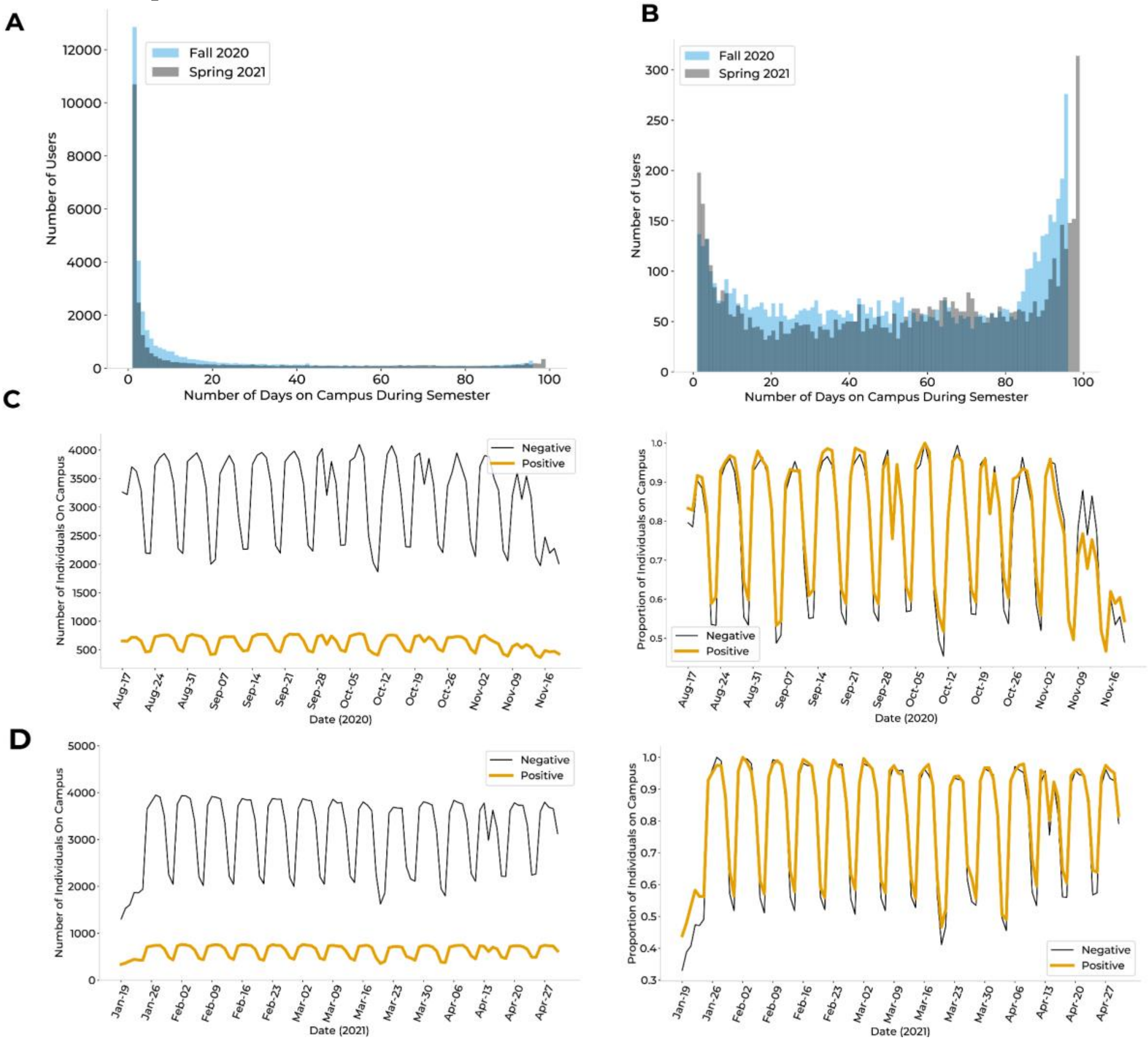

A. The number of days present on campus for all users found in the WiFi network, colored by semester. **B.** The number of days present on campus for all users found in the WiFi network after removal of unauthenticated users, non-students, and infrequent users, colored by semester. This cleaned WiFi proximity network was used for all future analyses. **C.** Number (left) and proportion (right) of positive students (orange) and negative students (black) on campus by day for Fall 2020. There was no significant difference in the distributions ($p = 0.282$), which correlated with one another (Pearson correlation: 0.976, $p < 0.001$). **D.** Number (left) and proportion (right) of positive students (orange) and negative students (black) on campus by day for Spring. There was no significant difference in the distributions ($p = 0.248$), which correlated with one another (Pearson correlation: 0.99, $p < 0.001$).

Figure S6. WiFi network access patterns by building and by semester. Related to Figure 3.

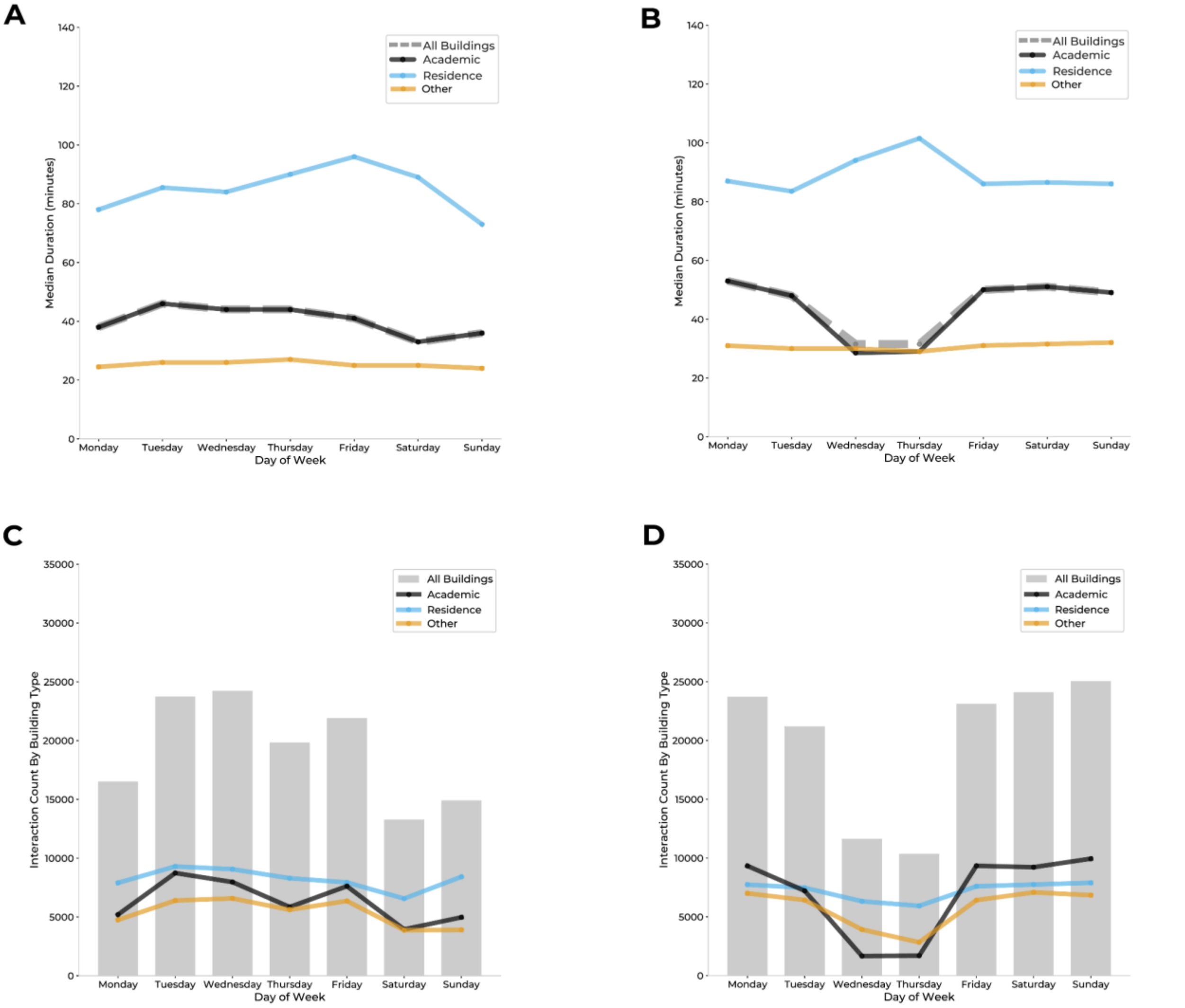

A-B. Median duration of access point (AP) connections in minutes, per day of the week and by building type (academic, residential, other, or all buildings) for Fall 2020 (**A**) and Spring 2021 (**B**). **C-D.** Daily number of AP connections, per day of the week and by building type (academic, residential, other, or all buildings) for Fall 2020 (**C**) and Spring 2021 (**D**).

Figure S7. Interaction patterns of individuals and pairs of individuals by testing status. Related to Figure 3.

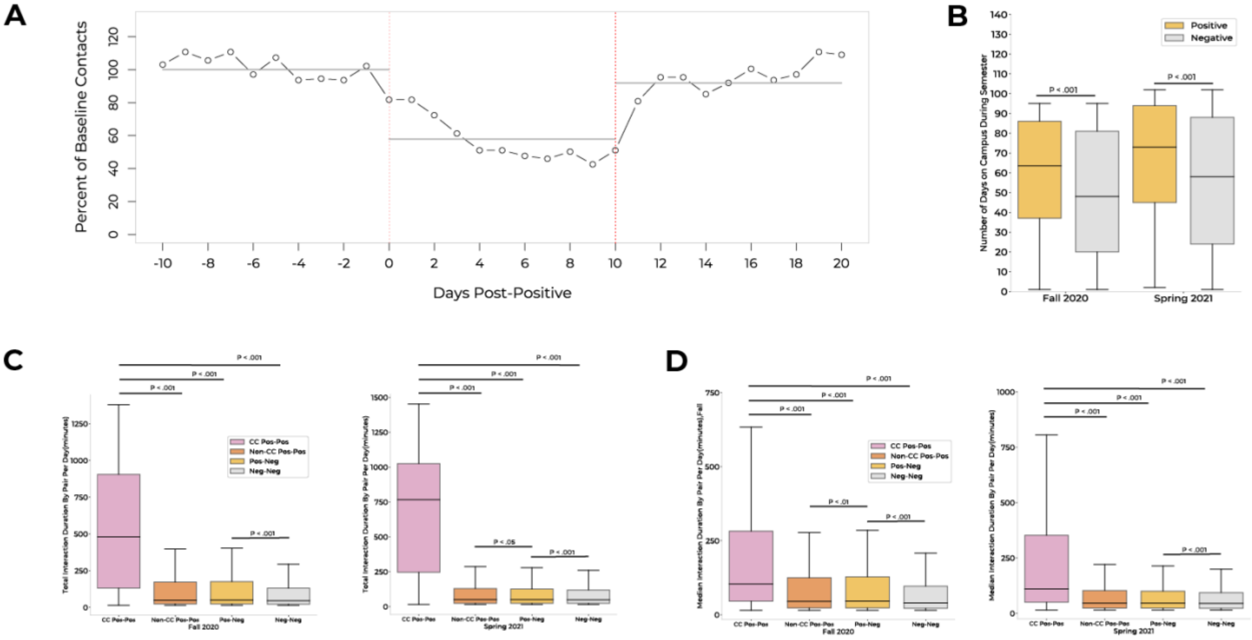

A. Percent of baseline WiFi contacts per day for the 30-day period surrounding the isolation period of positive cases. Day 0 was calculated as the earliest of symptom onset date and test date. Black lines indicate the median percent of baseline contacts (*y*) per individual for every day across the period (*x*). Gray bars indicate averages for three 10-day periods: pre-isolation (*i.e.*, baseline), isolation, and post-isolation. **B.** The number of days on campus for positive (orange) *vs.* negative (gray) individuals. **C.** Distributions of total pairwise daily interaction duration, for pairings categorized by testing status: CC Pos-Pos, pairs of pre-positive individuals with an association reported in manual contact tracing; Non-CC Pos-Pos, pairs of pre-positive individuals without an association reported in manual contact tracing; Pos-Neg, pairs of one pre-positive and one negative individual; Neg-Neg, pairs of two negative individuals. Pre-positive individuals were defined as students in the 10-day window prior to testing positive. **D.** Distributions of median interaction duration by pair per day, for pairings categorized by test status.

Figure S8. Attribute assortativity within the WiFi proximity network predicts daily case counts. Related to Figure 3.

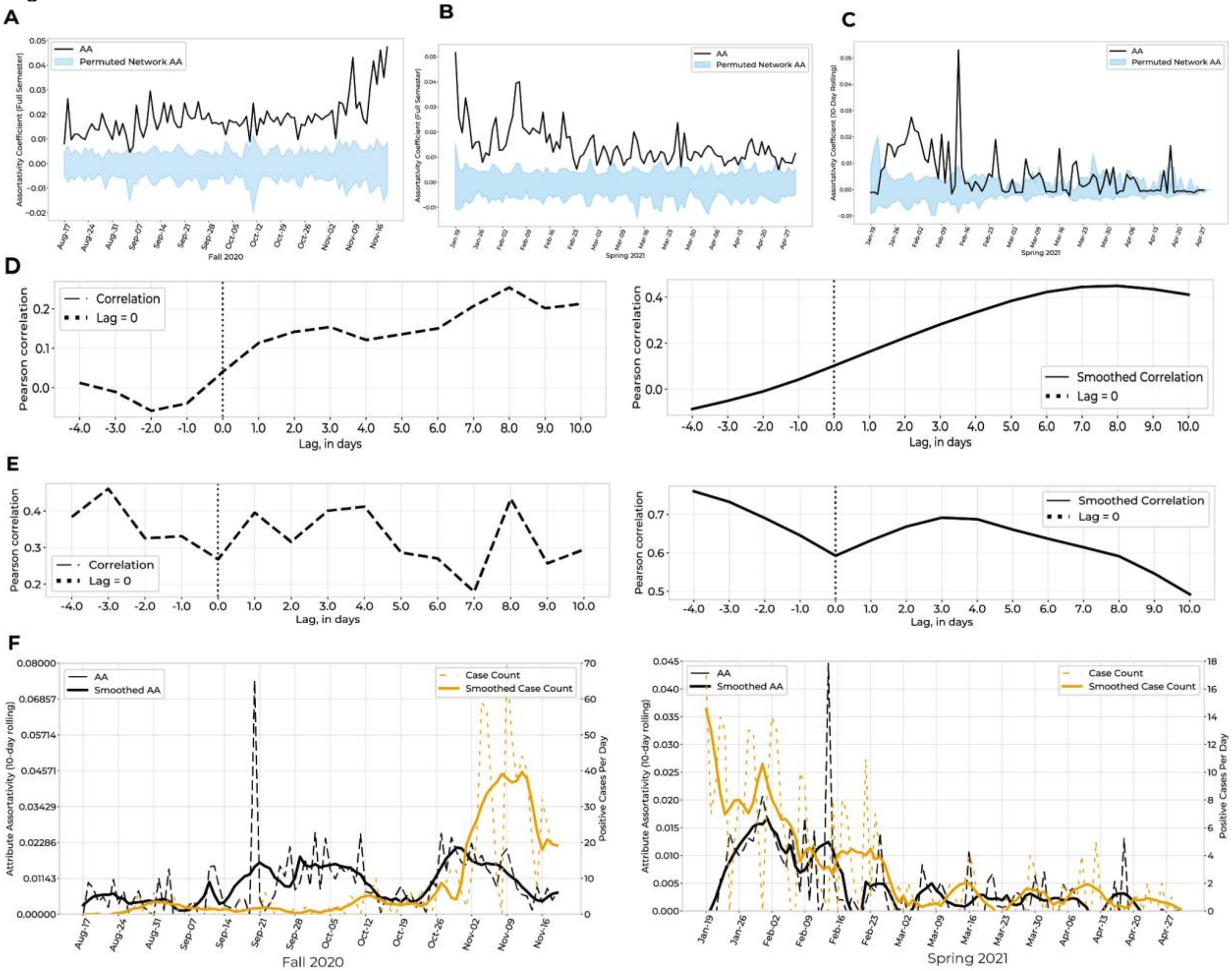

A-B. Comparison of attribute assortativity (AA) for positives *vs*. negatives, for Fall 2020 (**A**) and Spring 2021 (**B**). 95% Confidence Intervals (CI; blue) were calculated by permuting positive and negative labels for individuals within the proximity network each day. The AA of the proximity network (black) was above the upper bound of the CI for 98.9% (94/95 days) in the Fall 2020 semester and 98% (100/102 days) in the Spring 2021 semester, indicating significance with $p < 0.025$. **C.** We calculated the same metric with altered groupings: pre-positives (all individuals within a 10-day window prior to their positive test) *vs.* negatives (individuals not within a 10-day window prior to a positive test, regardless of overall semester testing status). The AA of the proximity network (black) was above the upper bound of the CI for 37.2% (38/102 days) in the Spring 2021 semester, indicating significance with $p < 0.025$. **D.** Correlations between daily AA (pre-positives *vs*. negatives) and daily case count with varying lag times, where both AA and case count are non-smoothed (top) or smoothed using Savitzky Golay filter (bottom), for Fall 2020. The highest correlation occurs with a lag time such that AA preceded the case count by 8 days (Pearson correlation = 0.253 for non-smoothed, 0.449 for smoothed). **E.** Cross-correlations between daily AA (pre-positives *vs*. negatives) and daily case count with varying lag times, where both AA and case count are non-smoothed (top) or smoothed using Savitzky Golay filter (bottom), for Spring 2021. The highest correlation occurs with lag time such that AA preceded the case count by 4 days (Pearson correlation=0.411 for non-smoothed; 3 days for smoothed, with correlation = 0.691). **F.** Unsmoothed case counts and unsmoothed AA *vs.* smoothed case counts and smoothed AA for Fall 2020 (left) and Spring 2021 (right).

<u>Figure S9.</u> Attribute assortativity within the WiFi proximity network when defining the “pre-positive” attribute as positive individuals missed by manual contact tracing. Related to Figure 3.

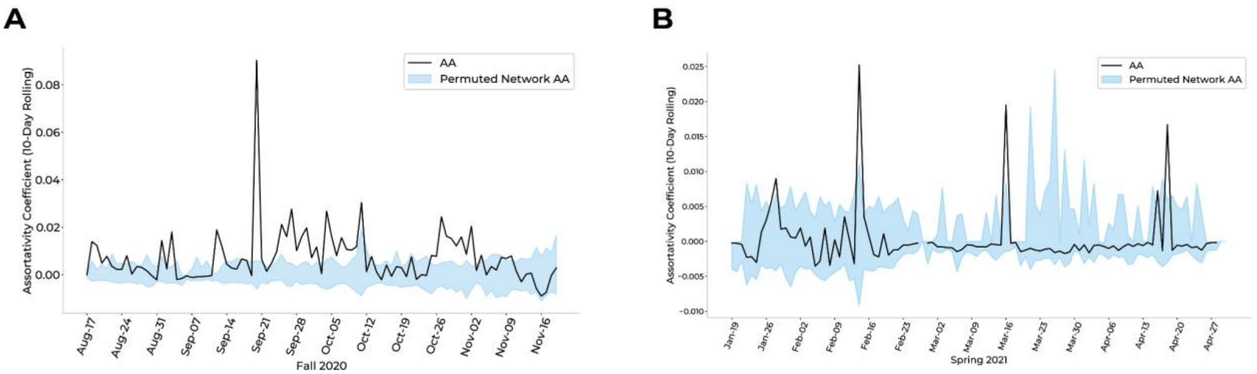

A-B. Comparison of attribute assortativity (AA) for pre-positives *vs*. negatives, for Fall 2020 (**A**) and Spring 2021 (**B**). 95% Confidence Intervals (CI; blue) were calculated by permuting pre-positive and negative labels for individuals within the proximity network each day. We calculated the same metric with pre-positives defined as individuals in the 10 days prior to their positive test missed by manual contact tracing, and negatives defined as individuals not in the 10 days prior to a positive test, regardless of overall semester testing status. The AA of the proximity network (black) was above the upper bound of the CI for 45.2% (43/95 days) of the Fall 2020 semester (**A**) and 8.8% (9/102 days) of the Spring 2021 semester (**B**), indicating significance with $p < 0.025$.

Figure S10. Sequenced wastewater samples are subject to increased degradation relative to clinical samples, but do not display wastewater-specific biases in coverage across the SARS-CoV-2 genome. Related to Figure 5.

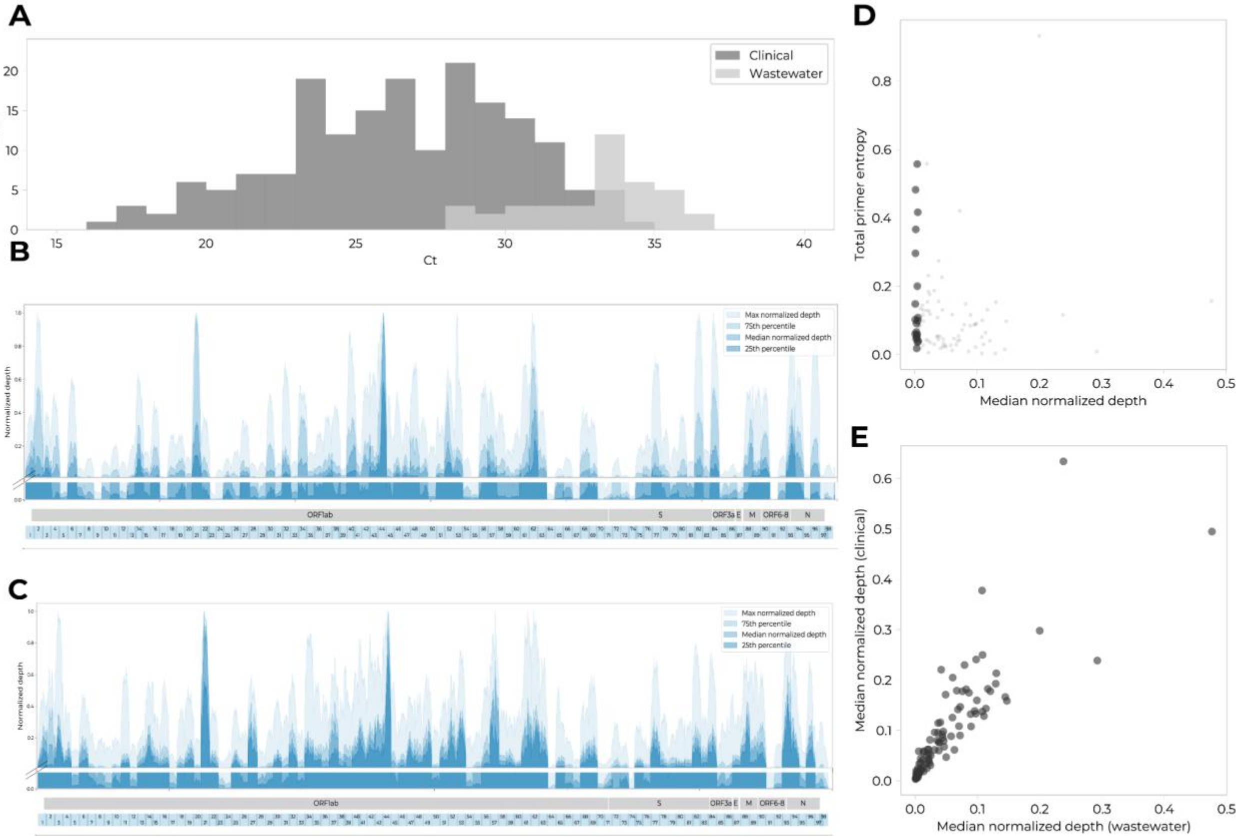

A. Cycle thresholds (Ct) for sequenced wastewater and clinical samples from Colorado Mesa University. **B-C.** Median normalized read depth per base, across all wastewater (**B**) and clinical (**C**) samples. The coloring indicates quartiles across all wastewater or clinical samples. Regions of lower depth in wastewater sequencing are of similarly low depth in clinical sequencing. **D.** Comparison of average amplicon read depth (*x* axis) and average amplicon primer entropy (*y* axis) for wastewater samples. We found no correlation between amplicon read depth and primer entropy (Pearson correlation = 0.02, p = 0.81). The 20 amplicons with lowest median normalized depth are shown as larger circles. **E.** Comparison of median normalized depth per amplicon between wastewater (*x* axis) and clinical (*y* axis) samples. There is a linear correlation between corresponding amplicons (Pearson correlation coefficient = 0.86; p < 0.001).

Figure S11. Presence of previously undetected mutations in sequenced wastewater samples suggests sequencing error. Related to Figure 5.

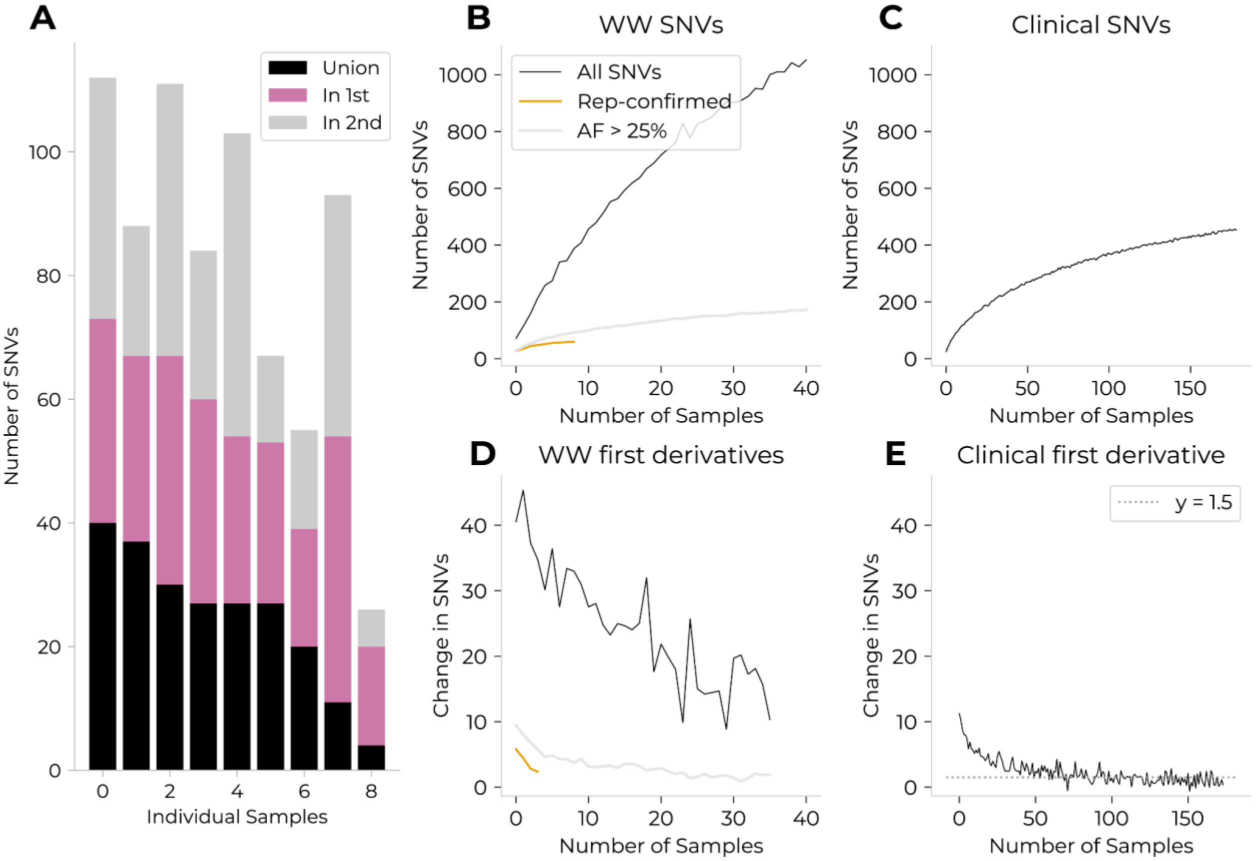

A. Distribution of single-nucleotide variants (SNVs) that were present in only one technical duplicate (pink and gray) or in both replicates (black). **B.** The number of unique SNVs expected for a given number of wastewater samples (see Methods). We additionally calculated the number of unique SNVs expected to pass an allele frequency quality control threshold of 25% (gray) or a technical replicate quality control (orange) for a given number of samples. **C**. The number of unique consensus-level SNVs expected for a given number of clinical samples. **D.** Smoothed (window=5) first derivative of unique wastewater SNVs per sample. Legend is the same as in panel (**B**). While the first derivatives of both replicate-confirmed SNVs (orange) and SNVs with an allele frequency greater than 25% (gray) approaches a rate of 1-3 new SNVs per sample, the first derivative for all SNVs of any allele frequency (black) approaches a rate of >10 new SNVs per sample. **E.** Smoothed (window=5) first derivative of unique clinical consensus-level SNVs per sample. The first derivative of all consensus-level SNVs seen in clinical samples approaches a rate of 1.5 new SNVs per sample. The comparison between (**D**) and (**E**) lends support to the hypothesis that most SNVs seen in wastewater samples are spurious, and that either confirming SNVs via technical replicates or a high allele frequency threshold is sufficient to remove the majority of spurious SNVs.

Figure S12. Social proximity patterns, transmission reconstruction networks, and experimental viral phenotypes associated with B.1.429.1. Related to Figure 6.

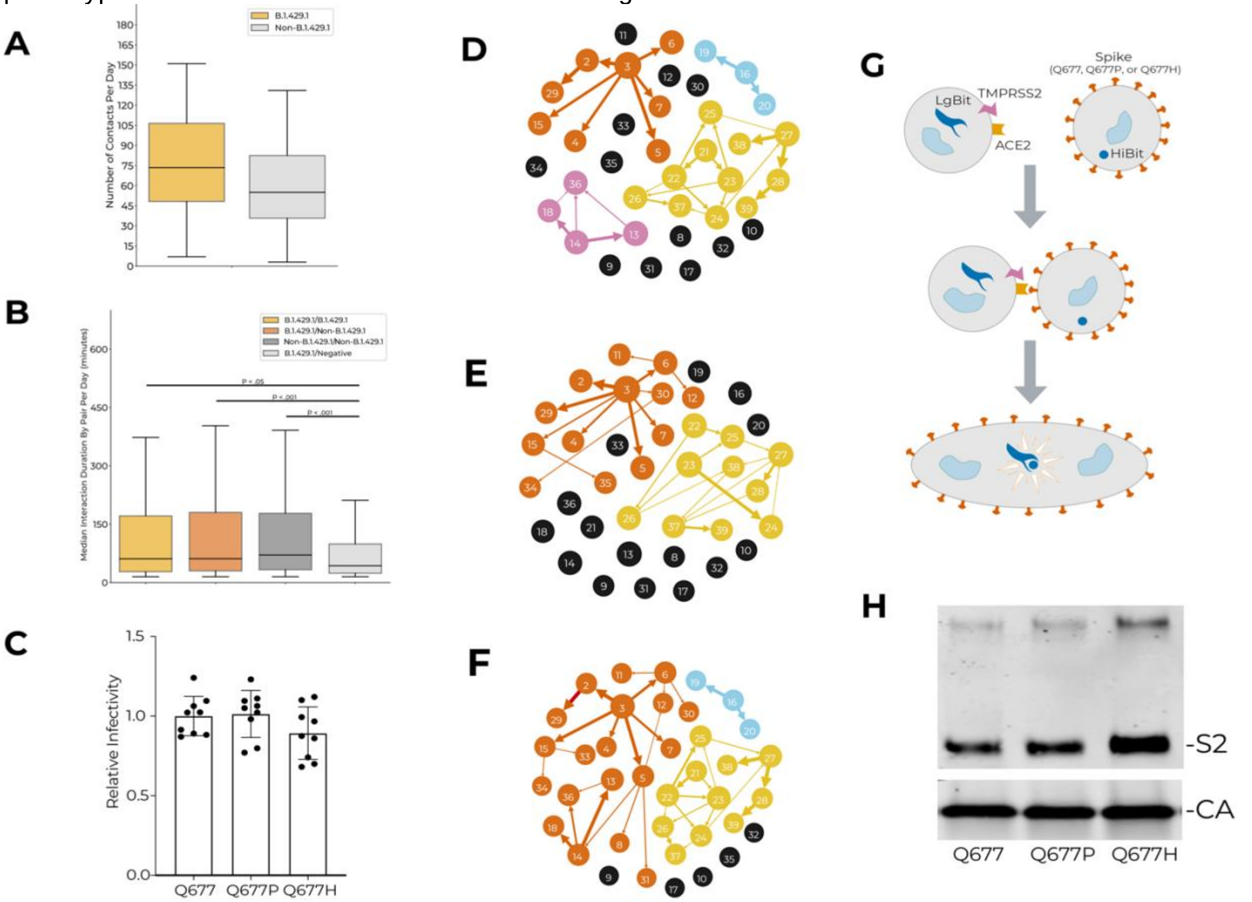

A. Average number of daily contacts per person in the 10 days prior to testing positive, for B.1.429.1-positive individuals *vs.* individuals contemporaneously infected with other lineages. p = 0.07. **B.** Pairwise median daily interaction duration for pairs of individuals with different viral lineages (see legend). Pairs of positive individuals interacted with one another in the 10 days prior to testing positive for longer durations than pairs of one B.1.429.1-positive and one negative individual. There were no other significant differences in pairwise interaction durations. **C.** Infectivity of viral pseudotypes with the S:Q677P or S:Q677H amino acid changes, relative to the ancestral allele. **D-F.** Transmission reconstruction networks for B.1.429.1 cases created with solely genomic information (**D**), solely manual contact tracing data (**E**), and both genomic information and WiFi-inferred 2-day contact data (**F**). **G.** The experimental strategy for assessing the fusogenicity of the S:Q677H or S:Q677P mutations relative to the ancestral residue, using two populations of cells: one expressing the modified Spike protein on the cell surface, and a second expressing the ACE2/TMPRSS2 receptors. The two populations were combined and fusion signal was assessed via HiBit-LgBit luminescent reporter. **H.** Western blot illustrating successful creation of viral pseudotypes bearing Spike protein with S:677Q, S:Q677H, and S:Q677P.
